# Transformation from passive health to proactive prevention: research progress on glucose-lowering components and its mechanism of food and medicine homology resources

**DOI:** 10.3389/fnut.2025.1681916

**Published:** 2026-01-05

**Authors:** Tianhao Li, Jie Li, Yinfei Sun, Dongqi Liu, Juntao Liu, Jing Han, Xiaoyu Chen, Wenyi Kang

**Affiliations:** 1 National R&D Center for Edible Fungus Processing Technology, Henan University, Kaifeng, China; 2 College of Agriculture, Henan University, Kaifeng, China; 3 School of Life Sciences, Henan University, Kaifeng, China; 4 Joint International Research Laboratory of Food & Medicine Resource Function, Kaifeng, Henan, China

**Keywords:** food and medicine homology resources, hypoglycemic effect, gut microbiota, hypoglycemic food, structure–activity relationship

## Abstract

Diabetes and its complications pose a threat to global human health. In the modern society, effectively preventing diabetes is a crucial means of safeguarding public well-being. Mounting evidence indicates that medicine-food homology foods possess significant medicinal and dietary value. However, these materials’ active compounds, and their structure–activity relationships on hypoglycemic function are unclear, this hinders the comprehensive utilization and development. In this review, 64 materials from 106 medicine-food homology foods claimed by the official possess a powerful hypoglycemic effects, according to statistics at the Web of Science, PubMed, and China National Knowledge Infrastructure (CNKI) databases. Current research indicates that these medicine-food homology foods contain *Astrogali radius, Persicae semen, Menthae haplocalycis herba, Houttuyniae herba*, *etc*. Terpenoids, flavonoids, alkaloids, phenylpropanoids, iridoids, and polysaccharides are regarded as their key bioactive compounds. This paper investigated and summried that the structure–activity relationships between these hypoglycemic constituents (triterpenoids, flavonoids, alkaloids, phenylpropanoid, iridoid and polysaccharide) and diabetes-related targets. In addition, this paper also reviews glucose-lowering mechanism of active compounds from medicine-food homology foods, including modulating digestive enzymes, regulating glucose metabolism (glucose absorption, gluconeogenesis and glycolysis), promoting insulin secretion, increasing insulin sensitivity, improving oxidative stress, reducing inflammatory response, promoting GLP-1 secretion and regulating gut microbiota. At the same time, the potential side effects of food with the same origin as medicine and food were discussed. Finally, the applications of medicine-food homology resources in glycemic management functional foods were reviewed. This provides a basis for the development of hypoglycemic functional food in the future, suggesting a shift in human health philosophy from treating existing diseases to preventing them, particularly for chronic metabolic diseases like diabetes.

## 1 Introduction

Diabetes is an endocrine disorder characterized by hyperglycemia, including Type 1 Diabetes Mellitus (T1DM), Type 2 Diabetes Mellitus (T2DM), and Gestational Diabetes Mellitus (1). According to reports from the International Diabetes Federation, by 2025, the global population with diabetes will reach 589 million. T2DM accounts for over 90% of all diabetes cases and is characterized by the presence of both insulin resistance and *β*-cell dysfunction. Chronic hyperglycemia and metabolic dysregulation can lead to systemic damage to tissues and organs, such as the liver, kidneys, cardiovascular system, and nervous system, endangering human health and life (2). The high incidence and increasing prevalence of T2DM impose a significant global economic burden, drawing worldwide attention. Many side effects of hypoglycemic medicine have occurred, although oral agents like biguanides, acarbose and thiazolidinediones exhibits a control blood glucose effect. For instance, acarbose can cause gastrointestinal disturbances (3), glibenclamide may lead to agranulocytosis and hypoglycemia (4), and metformin can cause lactic acidosis and gastrointestinal side effects (5). However, oral hypoglycemic drugs cannot prevent diabetes onset nor effectively improve its complications (such as nephropathy and cardiovascular disease). As an effective strategy, subcutaneous insulin injection carries risks of hypoglycemia and injection site reactions (6). Therefore, the development safe and effective functional food for preventing diabetes is extremely urgent.

Traditional Chinese Medicine (TCM) has a history of thousands of years in China and plays a vital role in healthcare. Compared to modern chemical drugs, TCM formulations not only lower blood glucose and effectively prevent or delay multiple diabetic complications, but also exhibit lower toxicity and fewer adverse reactions (7). The term “medicine-food homology foods material” refers to traditional Chinese medicinal materials that serve dual functions as both food and medicine, and are included in the Catalog of Substances That Are Both Food and Traditional Chinese Medicine (8, 9). According to Tradition published by the Ministry of Health, as of 2024, based on regulations such as the Food Safety Law of the People’s Republic of China and following safety evaluations, relevant national authorities have included a total of 106 materials in this catalog. From these, 64 medicine-food homology foods with blood glucose-lowering properties have been listed in the Supplementary Table S1 (Supplementary Table S1: A list of medicine-food homology foods with hypoglycemic effects is provided, including their medicinal materials names, plant sources, images, and geographical distribution. It covers a variety of common ingredients such as *Astrogali radius*, *Lablab semen album*, *Lilii bulbus*, and *Allii macrostemonis bulbus*). These materials are widely used in the functional food, include mulberry yogurt, blueberry Chinese yam dishes, spina date seed fruit wine, *poria cocos* pastries, and kudzu root vinegar. Intake of bioactive compounds from medicine-food homology foods helps reduce the risk of obesity, T2DM, cardiovascular diseases, and certain cancers (10, 11).

Current research on medicinal food homologous substances primarily focuses on the extraction of bioactive compounds, structural elucidation, efficacy evaluation, and their applications in food and health products (12, 13). Research indicates that triterpenoids and their glycosides, flavonoids, alkaloids, phenylpropanoids, iridoids, and polysaccharides play critical roles in regulating blood glucose. A total of 324 compounds isolated from these materials can modulate glucose metabolism by altering key proteins and enzymes involved in metabolic processes (such as (adenosine 5′-monophosphate (AMP)-activated protein kinase) AMPK and (phosphatidylinositol 3-kinase) PI3K) and by regulating gut microbiota homeostasis. Furthermore, the structures of these compounds and their hypoglycemic mechanisms are yet elucidated, this limits their application. Therefore, this review summarizes the characteristics (species, structures, etc.) of medicinal food homologous resources with glucose-regulating properties and analyzes their blood glucose-lowering mechanisms and the structure–activity relationships between these hypoglycemic constituents and diabetes-related targets. It also examines their current applications in food field. The aim is to provide a theoretical foundation for advancing the discovery and identification of novel hypoglycemic active compounds and to support the development of functional foods with blood glucose-lowering activity.

## 2 Hypoglycemic components from medicine-food homology foods materials

According to a literature search conducted in PubMed, CNKI, and Web of Science databases using the keywords “blood glucose” and “traditional Chinese medicine,” as well as “hyperglycemia” and “traditional Chinese medicine,” a total of 64 medicine-food homology foods materials were identified. This list includes 16 herbs with a frequency of use ≥20 times (The higher the utilization frequency of medicinal and food homologous substances, the more advanced their development, making them more suitable for creating functional foods). The results are shown in Supplementary Table S2 (Supplementary Table S2: The frequency of use of various medicine-food homology foods in regulating blood glucose has been statistically analyzed).

**Figure 1:**
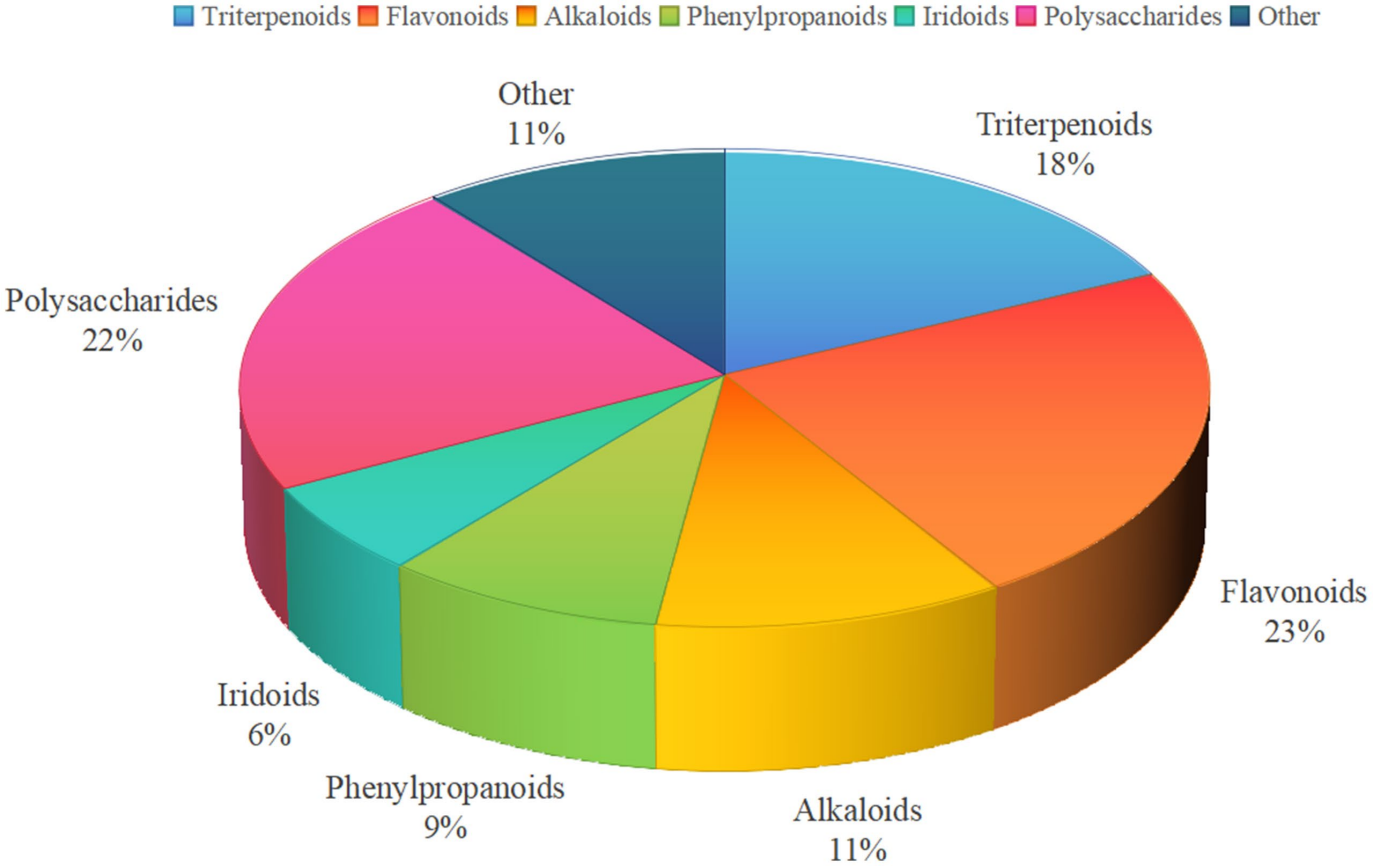
Structural types of hypoglycemic active ingredients from medicine-food homology foods.

Multiple bioactive components extracted from medicine-food homology foods—including triterpenoids, flavonoids, alkaloids, phenylpropanoids, iridoids, and polysaccharides—demonstrate significant blood glucose-regulating effects. A total of 324 compounds with hypoglycemic activity have been identified from 64 distinct medicine-food homology foods. As shown in Figure 1, analysis reveals that 75 flavonoids (23%), 58 triterpenoids (18%), 36 alkaloids (11%), 29 phenylpropanoids and related compounds (9%), 20 iridoids (6%), 71 polysaccharides (22%), and 35 other miscellaneous compounds (11%).

### 2.1 Triterpenes

Terpenoids constitute a significant proportion of naturally occurring compounds. They are defined as a class of compounds composed of isoprene structural units (C₅H₈)ₙ and their derivatives (14). Among them, triterpenoids constitute a major category of terpenoids and exhibit multiple pharmacological activities, including anticancer, immunomodulatory, and hypoglycemic effects (15–17).

Research indicates that triterpenoids can reduce blood glucose levels through multiple pathways. Maslinic acid from *Crataegus pinnatifida* Bge. (hawthorn) activates the AMPK/SIRT1 signaling pathway to enhance insulin sensitivity, thereby regulating blood glucose (18). Astragaloside IV from *Astragalus membranaceus* (Fisch.) Bge. modulates multiple signaling pathways (Including AMPK/SIRT1, PI3K/AKT, and JNK/Nrf2) to protect the liver and pancreas. It suppresses hepatic glycogenolysis by inhibiting glycogen phosphorylase activity and reduces hepatic glucose production by inhibiting glucose-6-phosphatase (G6Pase) activity. Furthermore, it lowers blood glucose levels through modulation of the gut microbiota and elevation of butyrate production (19–21). Glycyrrhetinic acid isolated from *Glycyrrhiza uralensis* Fisch. reduces insulin resistance by inhibiting the PI3K/Akt signaling pathway. Additionally, it restores normoglycemia through downregulation of G6pase and Pepck gene expression (22, 23). Ursolic acid isolated from *Cornus officinalis* Sieb. et Zucc. modulates multiple signaling pathways—including inhibition of the JNK/MAPK pathway and suppression of the AGEs-RAGE axis—while also restoring normoglycemia through inhibition of *α*-glucosidase and α-amylase enzymes. Additionally, reduction of oxidative stress levels constitutes a critical aspect of its blood glucose regulatory mechanisms (24–26). Malonylginsenoside Rb1 isolated from *Panax quinquefolium* L. activates the IRS1/PI3K/AKT signaling pathway, effectively upregulating GLUT4 and PPARγ protein expression to enhance insulin sensitivity, thereby reducing blood glucose levels in streptozotocin (STZ)-induced diabetic mice (27).

**Figure 2:**
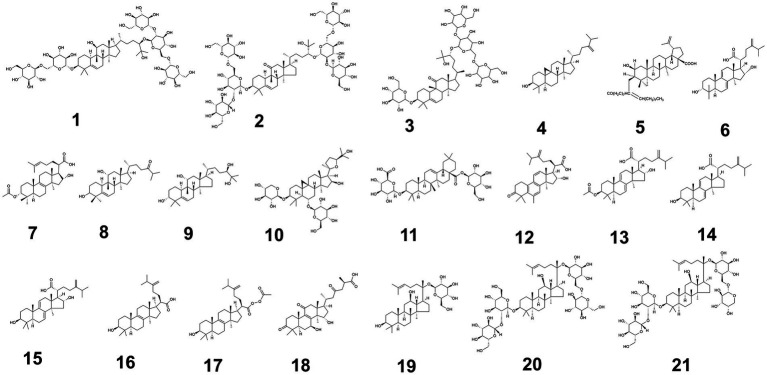
The structure of triterpenoids (1–21) with blood glucose-regulating effects.

In addition, Figure 2 shows the structure of triterpenoids (Figure 2 shows triterpenoid compounds 1–21, the others are provided in Supplementary Figure S1) with blood glucose-regulating effects, and Supplementary Table S3 shows glucose-regulating mechanisms of 58 triterpenoids isolated from medicine-food homology foods (Supplementary Table S3 provided the source, experimental models used, and hypoglycemic mechanisms of triterpenoids with hypoglycemic effects from medicine-food homology foods).

### 2.2 Flavonoids

Flavonoids are a class of polyphenolic natural products widely distributed in the plant kingdom, structurally characterized by the 2-phenylchromone backbone (28). They exhibit a spectrum of physiological and pharmacological activities, including antioxidant, antibacterial, antiviral, and hypoglycemic effects (29–33). Flavonoids extracted from medicinal-food homologous foods such as *A. membranaceus* (Fisch.) Bge., *Citrus reticulata* Blanco, *Glycyrrhiza uralensis* Fisch. and *Zanthoxylum bungeanum* Maxim. exhibit multiple mechanisms for blood glucose regulation.

Tangeretin ameliorates insulin resistance and promotes insulin secretion by inhibiting the MEK-ERK1/2 pathway and activating IR-AKT phosphorylation, ultimately restoring normal blood glucose levels (34). Flavonoids isolated from *Polygonatum kingianum* Coll. et Hemsl.—including nicotiflorin, narcissin, kaempferol-3-O-(2″-O-*β*-d-glucopyranosyl)-β-d-glucopyranoside, catechol, and kaempferol-3-O-*α*-(6″′-p-coumaroylglucosyl)-β-1,2-rhamnoside—regulate blood glucose through a dual mechanism inhibiting advanced glycation end-product (AGE) formation: source suppression and receptor coupling reduction. This process diminishes reactive oxygen species (ROS) generation and modulates the MAPK/NF-κB signaling pathway (35). Glabridin isolated from *Glycyrrhiza uralensis* Fisch. activates the PI3K/Akt pathway, thus modulating gluconeogenesis (36). Additionally, glabridin potentiates insulin secretion by restoring pancreatic *β*-cell function, thereby reestablishing glycemic homeostasis (37). Quercetin, isolated from plants such as *Crataegus pinnatifida* Bge. and *Sophora japonica* L., regulates glucose homeostasis not only through inhibition of *α*-glucosidase but also by activating the SIRT1/AMPK/NF-κB signaling pathway, thereby modulating inflammatory cytokines (38, 39).

**Figure 3:**
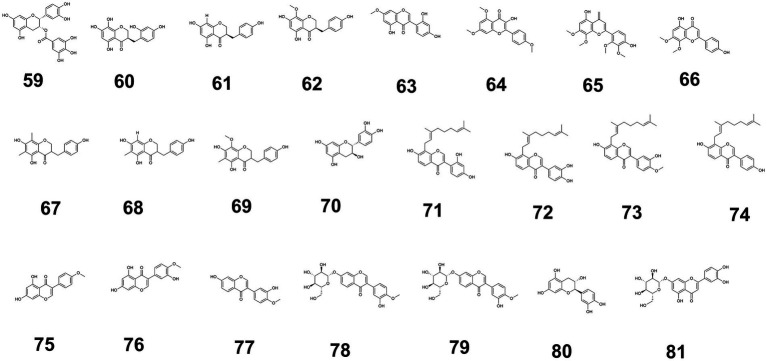
The structure of flavonoids (59–81) with blood glucose-regulating effects.

Additionally, the structures of flavonoids with blood glucose-regulating activity are illustrated in Figure 3 (Figure 3 shows flavonoids compounds 59–81, the others are provided in Supplementary Figure S2), while the glucose-modulating mechanisms of 75 flavonoids isolated from medicine-food homology foods are summarized in Supplementary Table S4 (Supplementary Table S4 provided the source, experimental models used, and hypoglycemic mechanisms of flavonoids with hypoglycemic effects from medicine-food homology foods).

### 2.3 Alkaloids

Alkaloids are nitrogen-containing organic compounds found in living organisms (40) and they possess anti-inflammatory, analgesic, and antioxidant properties, and other pharmacological effects (41). Alkaloids from medicine-food homology foods of traditional chinese medicine (TCM), exhibit anti-inflammatory, antimicrobial, vasodilatory, hypoglycemic, and anticancer properties, etc. (42). The *α*-glucosidase catalyzes the hydrolysis of α-glucose residues from the non-reducing end (43), playing a crucial role in regulating glucose metabolism in the body. Inhibiting its activity can delay glucose absorption, thereby reducing postprandial and fasting blood glucose levels.

Cepharadione B, 3-hydroxy-1,2-dimethoxy-5-methyl-5H-dibenzoindol-4-one, 4-hydroxy-1,2,3-trimethoxy-7H-dibenzoquinolin -7-one, and 7-oxodehydroasimilobine were isolated from *Houttuynia cordata* Thunb. They function as PTP1B inhibitors to exert hypoglycemic effects. (44). Higenamine 4′-O-*β*-d-glucoside isolated from *Nelumbo nucifera* Gaertn. activates the PI3K/AKT signaling pathway, upregulates GLUT4, and thereby promotes glucose uptake (45). N-cis-Feruloyloctopamine and four other alkaloids isolated from *Polygonatum odoratum* (Mill.) Druce exert hypoglycemic effects through inhibition of *α*-glucosidase activity (46). Neferine derived from *Nelumbo nucifera* Gaertn. exerts anti-inflammatory effects by elevating concentrations of LXA4 and LXB4 in streptozotocin (STZ)-induced diabetic mice, thereby alleviating insulin resistance (47).

**Figure 4:**
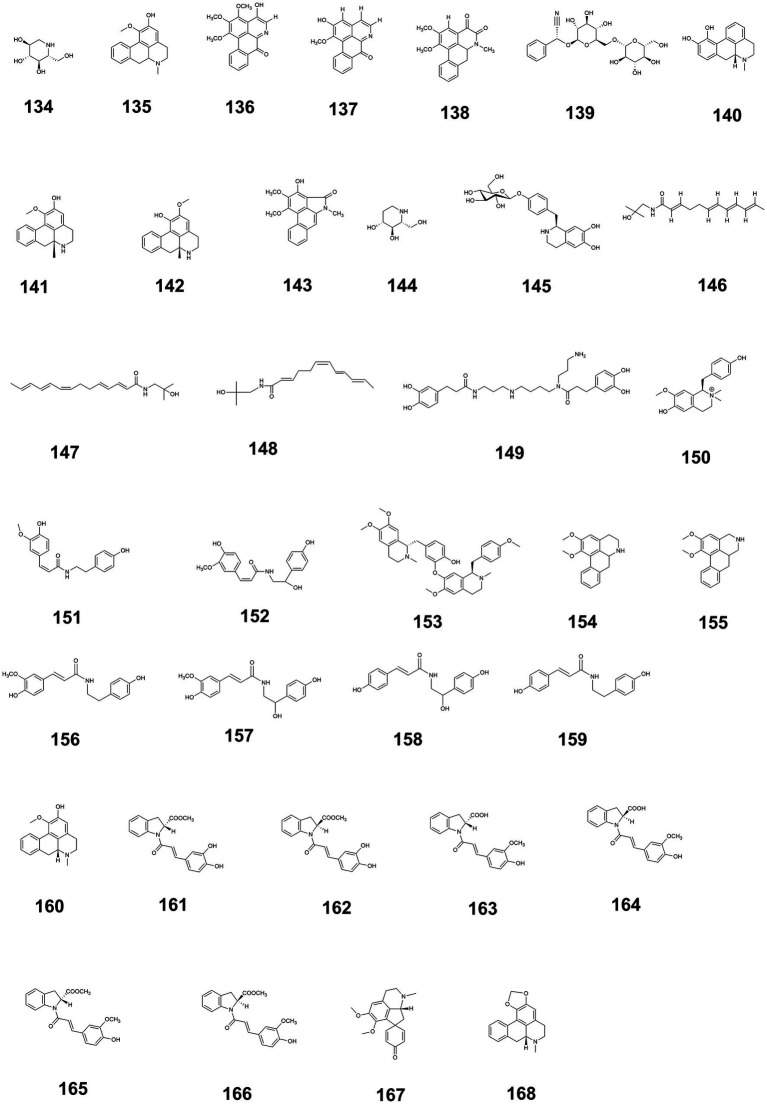
The structure of alkaloids with blood glucose-regulating effects.

Additionally, the structures of alkaloids exhibiting blood glucose-regulating activity are illustrated in Figure 4, while the glucose-modulating mechanisms of 36 alkaloids isolated from medicine-food homology foods are summarized in Supplementary Table S5 (Supplementary Table S5 provided the source, experimental models used, and hypoglycemic mechanisms of alkaloids with hypoglycemic effects from medicine-food homology foods).

### 2.4 Phenylpropanoids

Phenylpropanoids are a class of naturally occurring organic compounds widely distributed throughout the plant kingdom. They possess a fundamental structural skeleton derived from the aromatic amino acid phenylalanine, characteristically consisting of a benzene ring and a three-carbon side chain, collectively forming the characteristic C6-C3 skeleton (48). Phenylpropanoids, abundant in traditional Chinese medicine-food homology foods, demonstrate significant biological activities including anti-inflammatory, antioxidant, antibacterial, antiviral, and antitumor effects, as well as cardiovascular protective actions (e.g., vasodilation) (49–52). Notably, numerous studies demonstrate that phenylpropanoids—particularly caffeic acid, ferulic acid, and their derivatives (such as chlorogenic acid)—exhibit significant hypoglycemic potential (53, 54).

Phellopterin, isolated from *Angelica dahurica* Bentham et Hooker f., activates GPR119 to stimulate GLP-1 secretion (55). Eugenol from *Eugenia caryophyllata* Thunb. modulates hepatic glucose and lipid metabolism and suppresses insulin resistance by regulating the SHP/pFOXO1/PCREB/PEPCK/G6Pase signaling pathway. Additionally, it restores glycemic homeostasis in STZ-induced diabetic rats through modulation of key glycolytic enzymes—including hexokinase (HK), pyruvate kinase (PK), and glucose-6-phosphate dehydrogenase (G6Pase) (56, 57). Myristicin isolated from *Angelica dahurica* Bentham et Hooker f. activates the AMPK signaling pathway, upregulates GLUT4 expression, and promotes glucose uptake (58). P-Hydroxyphenyl butanone isolated from *Rubus chingii* Hu activates IRS-1 and SHP-1, thereby activating the AMPK/AKT signaling pathway to modulate glucose homeostasis (59).

**Figure 5:**
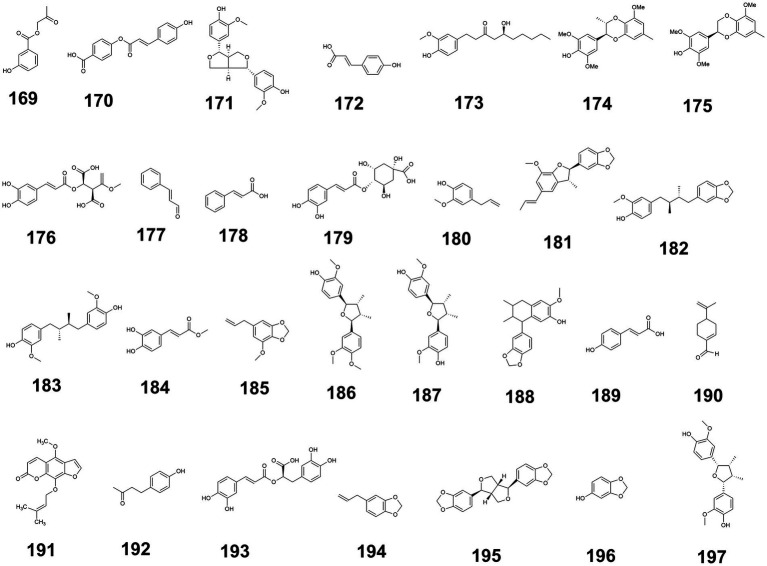
The structure of phenylpropanoids with blood glucose-regulating effects.

Additionally, the structures of phenylpropanoids exhibiting blood glucose-regulating activity are illustrated in Figure 5, while the glucose-modulating mechanisms of 29 phenylpropanoid compounds isolated from medicine-food homology foods are summarized in Supplementary Table S6 (Supplementary Table S6 provided the source, experimental models used, and hypoglycemic mechanisms of phenylpropanoids with hypoglycemic effects from medicine-food homology foods).

### 2.5 Iridoids

Iridoids are a class of monoterpenoid derivatives predominantly found in higher plants, notably dicotyledons. Their structural hallmark is a seco-iridoid skeleton featuring an oxygen-containing heterocycle (pyran or furan ring) fused to a cyclopentane moiety (60). These compounds are abundant in numerous traditional medicinal plants and exert broad-spectrum bioactivities, including significant anti-inflammatory, hepatoprotective, choleretic, antioxidant, antitumor, neuroprotective, and cardiovascular regulatory effects (61–63). Recently, accumulating studies have confirmed that iridoids possess well-documented hypoglycemic effects, establishing them as promising candidates for natural product research in diabetes prevention and management (64).

Geniposide from *Cornus officinalis* Sieb. et Zucc. regulates blood glucose through RBP4 suppression (reducing synthesis/secretion and modulating circulating levels), GLUT4 upregulation, AMPK-FoxO1-mediated hepatic gluconeogenesis inhibition, and *α*-glucosidase activity blockade (65, 66). Its aglycone genipin exerts complementary effects by: downregulating TNF-α/IL-6 expression to alleviate inflammation-driven insulin resistance; activating the IRS-1/PI3-K pathway to enhance GLUT4-dependent glucose uptake; and modulating JNK/AKT signaling to suppress hepatic oxidative stress and mitochondrial dysfunction in diabetic models (67–69). Notably, multiple iridoids from *Cornus officinalis* Sieb. et Zucc. consistently promote glucose uptake in insulin-resistant HepG2 cells, confirming their broad antidiabetic potential (70, 71).

**Figure 6:**
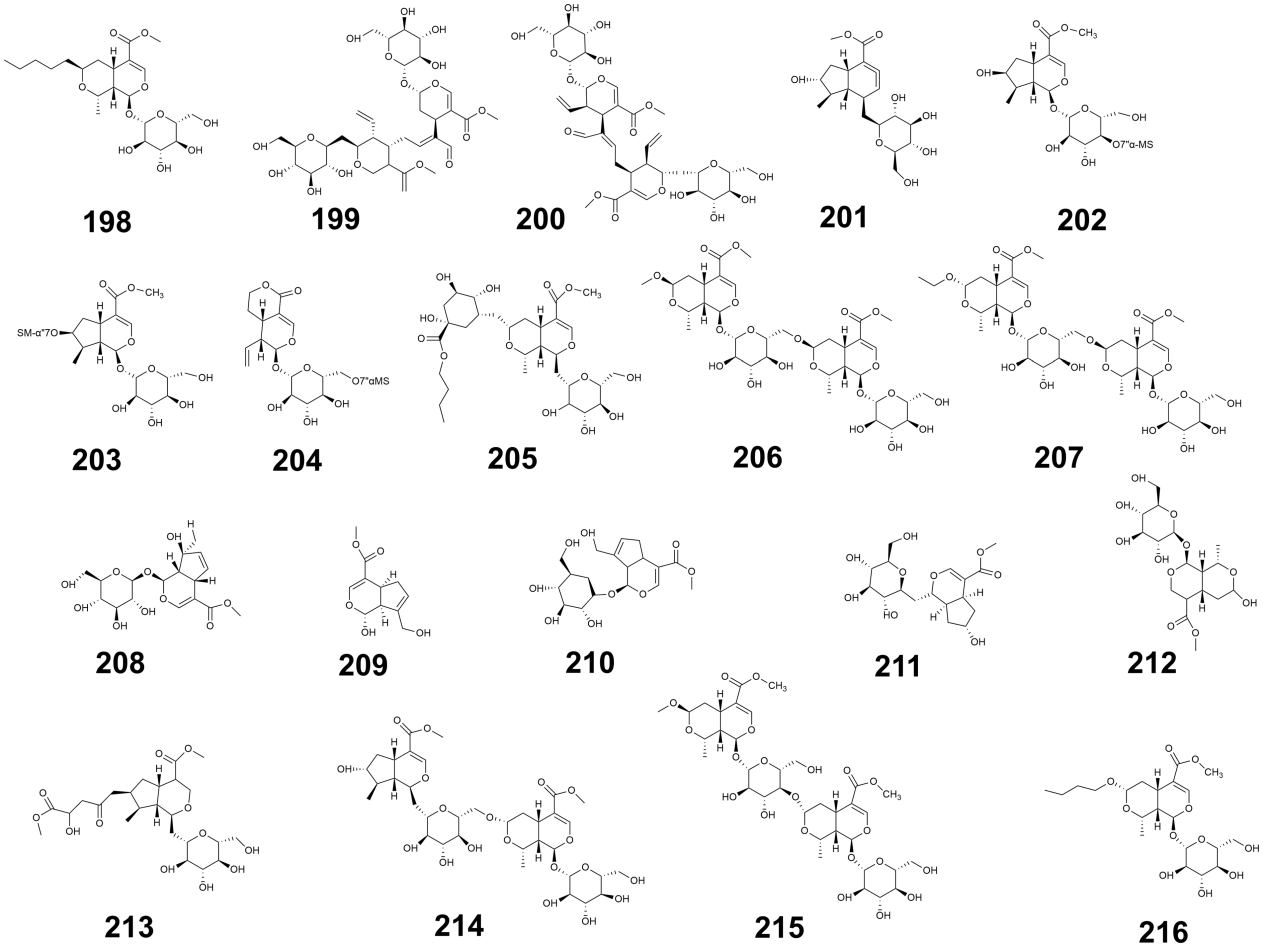
The structure of phenylpropanoids with blood glucose-regulating effects.

Additionally, the structures of iridoids with blood glucose-regulating activity are illustrated in Figure 6, while the glucose-modulating mechanisms of 20 iridoid compounds isolated from medicine-food homology foods are summarized in Supplementary Table S7 (Supplementary Table S7 provided the source, experimental models used, and hypoglycemic mechanisms of iridoids with hypoglycemic effects from medicine-food homology foods).

### 2.6 Polysaccharides

Polysaccharides, natural high-molecular-weight polymers with diverse bioactivities, are primarily formed through the condensation of ketoses, aldoses, or their derivatives (72, 73). In recent years, increasing numbers of plant-derived polysaccharides—including those from medicine-food homology foods—have been demonstrated to exhibit antibacterial, hypoglycemic, anti-inflammatory, and immunomodulatory functions (74–76).

Astragalus polysaccharides (APS), as primary bioactive constituents of *A. membranaceus* (Fisch.) Bge., demonstrate multifaceted glycemic regulation: APS activates AMPK signaling to stimulate glucose uptake (77), while AERP modulates gut microbiota to restore normoglycemia in db/db mice (78). The congeneric AMP achieves dual glycemic control through gut microbiota-mediated GLP-1 secretion enhancement and *α*-amylase inhibition, concurrently alleviating oxidative stress in diabetic models (79, 80). Finger citron polysaccharides exhibit complementary mechanisms—FCP-2 (from *Citrus medica* var. sarcodactylis) simultaneously inhibits α-glucosidase/α-amylase, suppresses AGEs formation, and activates PI3K/AKT signaling (81), while FCP40 demonstrates potent α-amylase inhibitory activity (82). *Lycium barbarum* polysaccharide (LBP) orchestrates glucose homeostasis through IRS/PI3K/Akt pathway activation with concurrent GLP-1 stimulation, coupled with NF-κB pathway inhibition to attenuate inflammation-driven insulin resistance (83, 84).

The glucose-modulating mechanisms of 71 polysaccharides isolated from medicine-food homology foods are summarized in Supplementary Table S8 (Supplementary Table S8 provided the source, experimental models used and hypoglycemic mechanisms of polysaccharides with hypoglycemic effects from medicine-food homology foods).

### 2.7 Other

Quinones, active peptides, and other medicine-food homology foods compounds demonstrate significant blood glucose-regulating properties. Three quinones isolated from *Cannabis sativa* L. exhibit potent *α*-glucosidase inhibition, as validated by PNPG assay (85). Six bioactive peptides from the same species not only effectively inhibit *α*-glucosidase but also alleviate insulin resistance in diabetic rats, thereby normalizing blood glucose levels (86). Ginseng Polypeptide from *Panax ginseng* C.A. Mey. activates both PI3K-Akt and MAPK signaling pathways in db/db mice, suppressing inflammation and oxidative damage to ameliorate diabetic pathology (87). Curcumin derived from *Curcuma longa* L. rhizomes modulates glycemic balance through multiple mechanisms: inhibiting the NF-κB pathway to reduce systemic inflammation (protecting pancreatic islets and liver), suppressing α-glucosidase activity, and activating PPAR-*γ* (88–91).

**Figure 7:**
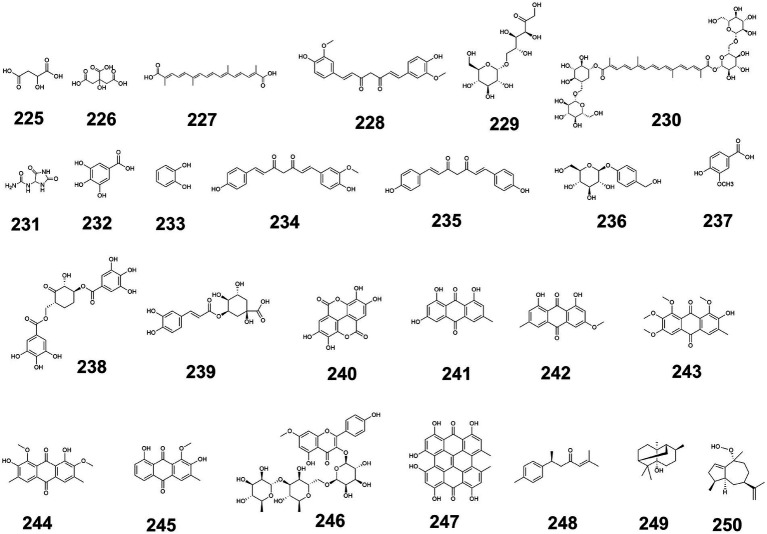
The structure of others from medicine-food homology foods with blood glucose-regulating effects.

Additionally, the structures of other bioactive compounds with blood glucose-regulating activity are illustrated in Figure 7, while the glucose-modulating mechanisms of 35 distinct compounds isolated from medicine-food homology foods are summarized in Supplementary Table S9 (Supplementary Table S9 provided the source, compound type, experimental models and their hypoglycemic mechanisms of other types of active compounds with hypoglycemic effects from medicine-food homology foods).

## 3 Structure–activity relationship of hypoglycemic components in medicine-food homology foods

The hypoglycemic capacity of medicine-food homology foods compounds fundamentally reflects the binding interactions between their molecular structures (e.g., shape, size, charge distribution, and functional groups) and specific biological targets (e.g., receptors, enzymes, ion channels) that regulate blood glucose homeostasis *in vivo*.

### 3.1 Triterpenoids

Triterpenoids’ hypoglycemic effect is closely related to their structure, the rigidity of the molecular skeleton, the position of substituents, and the stereochemical properties directly influence their binding affinity to targets and biological activity (92). Due to the rigidity of their cyclic structure, pentacyclic triterpenoids can bind more stably to the hydrophobic pockets of enzymes or receptors (93). Oleanolic acid with hypoglycemic effect possesses a rigid pentacyclic system formed by its A and E rings. The carboxyl group at the C-28 position can bind to PPARγ through electrostatic interactions, further activating its receptor to enhance glucose uptake in adipocytes (94). The *β*-orientation of the C-3 hydroxyl group strengthens hydrogen bonding with the active site of *α*-glucosidase, thereby inhibiting the breakdown of carbohydrates in the intestines and reducing postprandial blood glucose levels (95). The carboxyl group at the C-17 position of ursolic acid directly forms hydrogen bonds with Arginine132 (ARG132) and Aspartic Acid128 (ASP128) of AMPK, thereby activating the AMPK signaling pathway to enhance glucose transport in skeletal muscle. Additionally, the moderate polarity of ursolic acid balances its target affinity and cellular penetration efficiency (96, 97).

### 3.2 Flavonoids

Flavonoids, characterized by a 2-phenylchromone core structure with a C6-C3-C6 carbon skeleton. The hydroxyl group at the C-6 position of the A-ring is identified as an essential functional group for their hypoglycemic activity. Hydroxyl substitutions at C-5, C-6, and C-7 positions are associated with hypoglycemic activity, and introduction of any substituent at C-8 significantly may diminish their glucose-lowering efficacy (98). Specifically, the C-6 hydroxyl group of isoliquiritigenin can interact with Asp202 and Arg400 residues of *α*-glucosidase to form hydrogen bonds, stabilizing the enzyme-inhibitor complex through additional hydrophobic interactions (99). The hydroxyl group at the C-3 position of the B-ring and the glycosyl group at the C-3 position of the C-ring play critical roles in binding stability (100). The C2-C3 double bond of flavonoids can stabilize hydroxyl groups, further activating AMPK and inhibit α-glucosidase by flavonoids, thereby reducing blood glucose levels. In addition, the C2-C3 double bond of glycitin, cynaroside possess a powerful antioxidant capacity, this facilitates AMPK pathway activation to regulate glucose homeostasis (101, 102).

### 3.3 Alkaloids

The steric conformation of alkaloids can directly affect their binding ability to hypoglycemia-related targets. The orientation of polar groups such as hydroxyl groups and amino groups in the conformation can be complementary with the amino acid residues in the active center of the enzyme, competitively inhibiting the decomposition of carbohydrates (103). The planar fused-ring conformation of alkaloids may bind to the *α* subunit of AMPK through hydrophobic interactions, further promoting the translocation of GLUT4 and enhancing glucose uptake (103, 104). The conformational changes of the flexible side chains may affect the binding to nuclear receptors, regulating insulin sensitivity (105).

### 3.4 Phenylpropanoid

The hypoglycemic activity of phenylpropanoids, encompassing simple phenylpropanoids, coumarins, and lignans, exhibits strong structural dependency. Core (Structure–activity relationship)SAR principles include: In phenylpropanoids, the number and position of phenolic hydroxyl groups are defining characteristics. Compounds possessing ortho-dihydroxy structures (catechol moieties), due to their potent antioxidant capacity, can effectively enhance insulin sensitivity and modulate glucose-regulating pathways (e.g., AMPK). Generally, increased hydroxylation (particularly at ortho/para positions) correlates with enhanced activity (106). In phenylpropionic/cinnamic acid derivatives, the *α*, *β*-unsaturated carbonyl system at the terminal of the propenoic side chain functions as an electrophilic Michael acceptor pharmacophore. This moiety mediates hypoglycemic effects by targeting key enzymes and signaling molecules (e.g., *α*-glucosidase, PTP1B, Keap1/Nrf2), where conjugation is essential for bioactivity. For coumarins, oxygenated substitutions at C6/C7—especially 6,7-dihydroxy configurations significantly enhance *α*-glucosidase inhibition rate, antioxidant potency, and glucose uptake (107, 108). Lignan activity depends critically on complex scaffold diversity (e.g., dibenzylbutyrolactone, tetrahydrofuran, dibenzocyclooctadiene) and precise substitution patterns (hydroxy, methoxy, methylenedioxy). In addition, as potent PPARγ agonists, dibenzocyclooctadiene-type lignans can improve insulin sensitivity (109).

### 3.5 Iridoid

The hypoglycemic efficacy of iridoids exhibits significant structural dependence with 3 key SAR principles. (1) The epoxy-containing cyclopentane scaffold (C1-C9 bond) is essential for maintaining activity, ring-opening or its reduction dramatically diminishes efficacy (69, 71, 110). (2) Substituents at C4 directionally modulate biological actions, methyl substitution enhances hepatic glycogen synthesis, while carboxyl groups (e.g., in geniposide) facilitate target protein binding (65). (3) In iridoids, phenolic hydroxyl groups and unsaturation patterns play a critical role in glucose-lowering effects through a synergistic mechanism. The C7-C8 double bond and phenolic hydroxyls cooperatively improve insulin resistance via PI3K/AKT pathway activation and antioxidant potentiation (69).

### 3.6 Polysaccharides

The hypoglycemic activity of polysaccharides is influenced by their relative molecular weight, chain conformation, monosaccharide composition, and glycosidic bond types (111–113). For polysaccharides with hypoglycemic activity derived from medicine-food homology foods, their molecular weight typically ranges between 10 and 50 kDa (114, 115). Hypoglycemic-active polysaccharides often include arabinose, galactose, glucose, and xylose. Additionally, the presence of uronic acids (such as mannuronic acid) may enhance polysaccharides’ hypoglycemic activity (116, 117). Polysaccharides’ 1 → 3 and 1 → 4 glycosidic bonds play critical roles in lowering glucose, and this also supports their ability to regulate gut microbiota, thereby alleviating diabetes (118).

## 4 Blood glucose-regulating mechanism of medicine-food homology foods

### 4.1 Digestive enzyme

*α*-Amylase, α-glucosidase, sucrase, and maltase constitute key digestive enzymes in the human intestine, playing essential roles in energy metabolism (119, 120). Inhibiting these enzymes regulates the rate of carbohydrate breakdown and absorption, attenuates postprandial glycemic excursions, and achieves glycemic control—representing a critical hypoglycemic mechanism of medicine-food homology foods compounds. Specific inhibitors include: p-Coumaric acid and luteoforol from *Morus alba* L., potent sucrase and maltase inhibitors that effectively manage postprandial hyperglycemia (121). *α*-Amylase—a carbohydrate hydrolase cleaving α-1,4-glycosidic bonds in amylose/amylopectin—is inhibited by compounds like ganoderic acid [a triterpenoid from *Ganoderma lucidum* (Leyss. ex Fr.) Karst.], which suppresses salivary/pancreatic *α*-amylase activity to delay starch digestion (122). Additional α-amylase inhibitors include ursolic acid, 6″′-vanilloylspinosin, and amygdalin (100, 123). α-Glucosidase hydrolyzes residual oligo−/disaccharides into glucose, driving postprandial hyperglycemia; thus its inhibition is a key T2DM therapeutic strategy. Natural inhibitors from medicinal foods include five alkaloids form *Polygonatum odoratum* (Mill.) Druce (N-cis-Feruloyloctopamine, N-trans-p-Coumaroyloctopamine, N-trans-Feruloyloctopamine, N-trans-p-Coumaroyltyramine, N-trans-Feruloyltyramine) with potent α-glucosidase inhibition (46), further exemplified by (+)-pinoresinol (*Sesamum indicum*), loganin (*Cornus officinalis*), and polysaccharide FCP-2-1 (*Citrus medica* var. sarcodactylis) (26, 81, 124).

### 4.2 Glucose metabolism

#### 4.2.1 Glucose uptake

**Figure 8:**
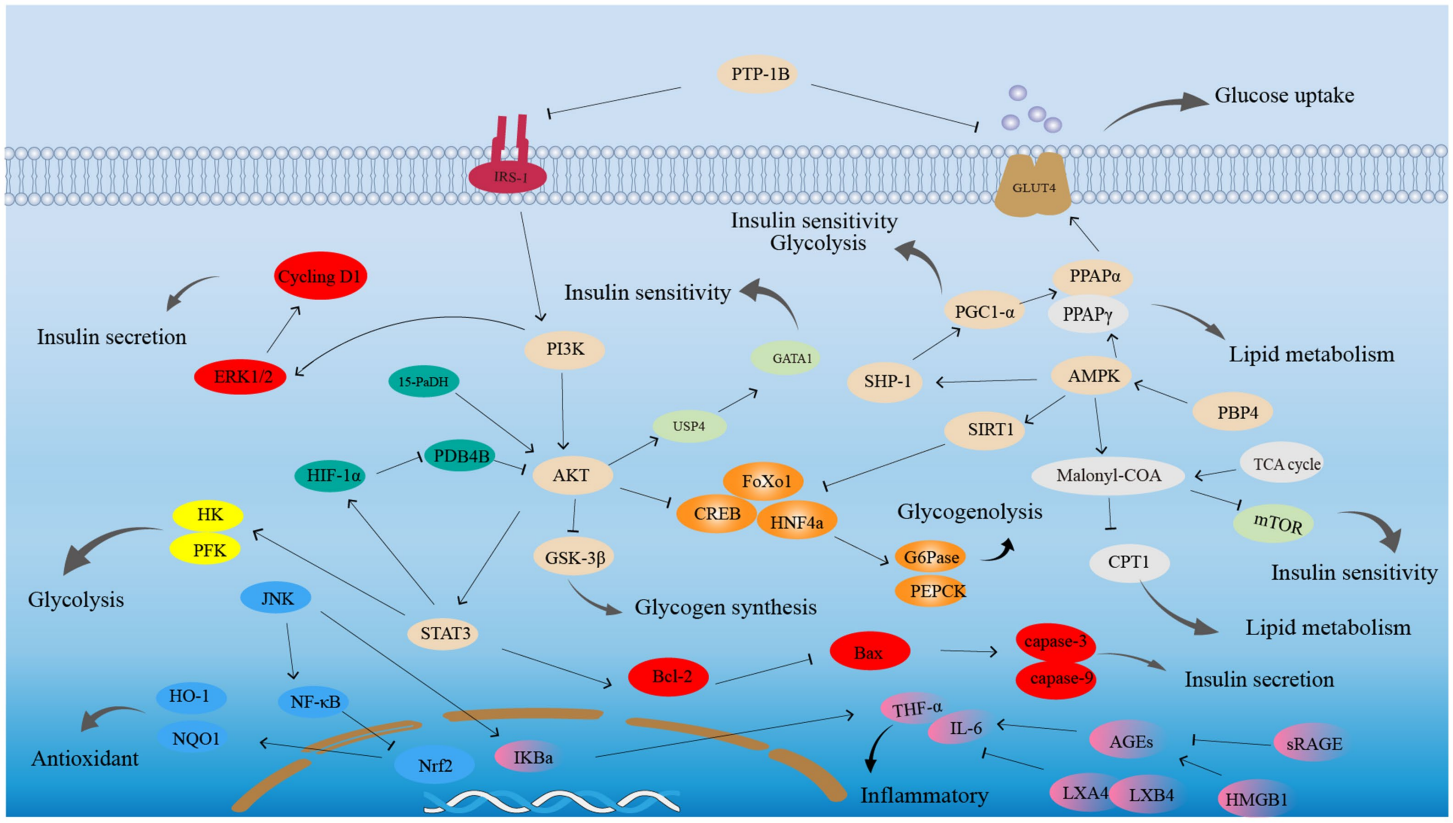
Hypoglycemic mechanisms of medicine-food homology foods bioactives. “→” Represents the normal metabolic pathway process and “T” represents the inhibition of that metabolic process.

Modulating glucose transporters to enhance glucose uptake constitutes an effective therapeutic strategy for diabetes. Glucose transporters comprise two categories: SGLTs (sodium-glucose cotransporters) utilizing secondary active transport, and GLUTs (glucose transporters) facilitating diffusion. Upregulation of GLUT2 and GLUT4 in hepatic and peripheral tissues (e.g., white adipose tissue, skeletal muscle) significantly enhances glucose uptake (125, 126). Twelve iridoids from *Cornus officinalis* Sieb. et Zucc. (cornuofficinaliside F, neocornuside A, neocornuside F, etc.) stimulate glucose uptake in insulin-resistant HepG2 cells, potentially through transporter modulation that delays glucose absorption and reduces postprandial hyperglycemia (70, 71, 127). Astragaloside IV activates IRS1/AKT signaling, upregulates GLUT4 expression, and stimulates glucose uptake in C2C12 myotubes to regulate glucose homeostasis (21), while protodioscin upregulates GLUT4 and enhances glucose uptake in diabetic mice (128). PPAR-*γ* regulates cellular glucose absorption by influencing GLUT4 expression and translocation: Ginsenoside Rg3 activates PPAR-γ to upregulate GLUT4 (129), daidzein enhances PPAR-γ activity (130), and dehydroglyasperin D with curcumin function as PPAR-γ activators (89, 131). Additional mechanisms include: ginsenoside CK upregulating GLUT2 in MIN6 pancreatic *β*-cells (128); polysaccharide F31 from Ganoderma lucidum activating AMPK signaling to upregulate GLUT4 (128); and LBP (*Lycium barbarum* polysaccharide) activating IRS/PI3K/Akt signaling to promote GLUT2-mediated uptake (84). Intestinal glucose absorption inhibitors include: five Polygonatum odoratum flavonoids inhibiting GLUT2/SGLT1 activity (126); p-coumaric acid and luteoforol suppressing GLUT2/SGLT1 expression (121); and ginsenoside Rh1 inhibiting SIRT1 (132). PTP1B inhibitors (kaempferol, ginnalin A, alaternin, APS) counteract PTP1B-mediated GLUT4 translocation inhibition, promoting glucose uptake (133–137). Figure 8 illustrates these hypoglycemic mechanisms of medicine-food homology foods bioactives.

#### 4.2.2 Glycogen synthesis

Glycogen synthesis as a form of energy storage. When blood glucose levels are elevated, the body recruits glucose and converts it into glycogen—a mechanism by which healthy organisms maintain glucose metabolic homeostasis (138). Glycogen synthase (GS) is the key enzyme in the glycogen synthesis pathway; increased GS activity enhances glycogen synthesis. Lily polysaccharide III promotes glycogen synthesis, restoring normoglycemia in STZ-induced diabetic mice (139). Hydroxy-*α*-sanshool activates the PI3K/Akt/GSK-3β/GS signaling pathway to increase glycogen synthesis and regulate glucose balance (140). Naringin inhibits GSK-3β, thereby modulating GS activity and glycogen metabolism to maintain normoglycemic concentrations (141). Glycyrrhetinic acid similarly activates the PI3K/Akt/GSK-3β pathway to promote glycogen synthesis (22). Ginsenoside Rb1 modulates the 15-PGDH/PGE2/EP4 pathway to enhance hepatic glycogen synthesis, reducing blood glucose concentration (142).

#### 4.2.3 Glucolysis

Glycolysis is the central metabolic pathway within cells for breaking down glucose, and its accelerated progression directly consumes glucose from the blood, thereby lowering blood sugar levels (143). Polysaccharide CERP1 isolated from *Codonopsis pilosula* (Franch.) Nannf. can effectively enhance the expression of HK (hexokinase) and PK (pyruvate kinase) to promote glycolysis (144). Tangeretin regulates hexokinase, pyruvate kinase, lactate dehydrogenase, and glucose-6-phosphatase, thereby inhibited gluconeogenesis and promoted glycolysis (145). Ginsenoside Rb1 activates the PI3K/AKT/STAT3 signaling pathway, enhances the expression of GCK (glucokinase), PFK (phosphofructokinase), and PKM (pyruvate kinase M), promoted glycolysis, and regulates blood glucose balance (146). Besides, polysaccharide GPS isolated from *Panax ginseng* C.A. Mey activates the PGC-1α pathway to promoted glycolysis (147).

#### 4.2.4 Gluconeogenesis

Gluconeogenesis is the process by which the body synthesizes glucose anew from non-carbohydrate precursors (such as lactate, glycerol, and glucogenic amino acids) in the liver and kidneys during starvation or prolonged fasting (148). Astragaloside IV inhibits gluconeogenesis by activating the AMPK-SIRT1 and PI3K/AKT signaling pathways, thereby suppressing the production of key enzymes (19, 149, 150). Ginsenoside CK suppresses gluconeogenesis and regulates blood glucose by upregulating the AMPK signaling pathway, inhibiting the expression of PGC-1α, HNF-4α, and FoxO1, and consequently inhibiting phosphoenolpyruvate carboxykinase (PEPCK) and glucose-6-phosphatase (G6Pase) (151, 152). Ginsenoside Rd. restores blood glucose balance by effectively inhibiting FoxO1 activity, thereby reducing the expression of gluconeogenic genes (153). Glycyrrhetinic Acid inhibits gluconeogenesis by suppressing HNF4α expression and subsequently inhibiting G6Pase and PEPCK (154). Glabridin can activate the PI3K/Akt pathway to modulate gluconeogenesis (36). Geniposide inhibits hepatic gluconeogenesis by regulating the AMPK-FoxO1 signaling pathway (65). Furthermore, polysaccharides WGPA isolated from *Panax ginseng* C.A. Mey and LBP isolated from *Lycium barbarum* L. also possess the ability to inhibit gluconeogenesis (84, 155).

### 4.3 Promote insulin secretion

Insufficient insulin secretion is a key factor in hyperglycemia, and protecting pancreatic islet cells is crucial for promoting insulin production (156). Mogroside V stimulates insulin secretion to regulate blood glucose balance (157). Chikusetsusaponin IVa repairs pancreatic *β*-cells, enhances insulin secretion, and alleviates insulin resistance (158). Similarly, Ginsenoside Re also repairs β-cells and mitigates insulin resistance (159). Jujuboside A protects islet cells and ameliorates insulin resistance by inhibiting the Bax/caspase-9 signaling pathway, thereby reducing apoptosis in pancreatic tissue (160). Nobiletin protects pancreatic β-cells by modulating the Bcl-2/Bax/Caspase-3 signaling pathway (161). Hyperoside suppresses islet cell damage in diabetic mice by downregulating the p65/NF-κB and ERK/MAPK signaling pathways (162). Licochalcone-A activates the IRS-2/PI3K/AKT pathway in diabetic mice, protecting β-cells and stimulating insulin secretion (163). Vanillic acid protects pancreatic β-cells by activating the ERK1/2 signaling pathway (164).

### 4.4 Increase insulin sensitivity

When insulin sensitivity declines, the body’s utilization of insulin decreases accordingly. Enhancing insulin sensitivity is therefore another crucial strategy for regulating blood glucose (165). Neferine possesses the ability to enhance insulin sensitivity (166). Similarly, 1-Deoxynojirimycin also improves insulin sensitivity (167). Maslinic acid activates the AMPK/SIRT1 signaling pathway in diabetic mice, enhancing insulin sensitivity and regulating blood glucose homeostasis (18). Tangeretin enhances insulin sensitivity by inhibiting the MEK-ERK1/2 pathway in hepatocytes of diabetic mice (34). Mitochondrial dysfunction is one cause of reduced insulin sensitivity. Ginsenoside Rg5 protects mitochondrial function and thereby promotes normal ATP production, restoring insulin sensitivity by activating the AMPK/SIRT1/PGC-1α signaling pathway (168). Jujuboside A also protects mitochondrial function and restores insulin sensitivity by activating the AMPK/mTOR signaling pathway (160). Genipin improves blood glucose levels by regulating the JNK/AKT signaling pathway in diabetic mice, inhibiting hepatic oxidative stress and mitochondrial dysfunction (68). Retinol-binding protein 4 (RBP4), a member of the hydrophobic retinol-binding protein (RBP) family, is negatively correlated with insulin sensitivity. Geniposide improves systemic insulin sensitivity and lowers blood glucose by inhibiting RBP4 synthesis and secretion, modulating circulating RBP4 levels, and thereby regulating the AMPK signaling pathway (66). Ubiquitin-specific peptidase 4 (USP4), a deubiquitinating enzyme, removes ubiquitin from the insulin receptor to inhibit its degradation, thereby maintaining cell surface insulin receptor levels. Gastrodin promotes USP4 expression by activating the PI3K/AKT pathway to facilitate GATA1 phosphorylation. This reduces ubiquitination and degradation of the insulin receptor, restoring insulin sensitivity (169).

### 4.5 Improve oxidative stress

Oxidative stress refers to an imbalance between oxidation and antioxidant defenses within the body, which can lead to insulin resistance, pancreatic *β*-cell dysfunction, and various diabetic complications (170). Reactive oxygen species (ROS), as intermediates of oxidative stress, can damage pancreatic β-cells, impair insulin secretion, and disrupt blood glucose homeostasis. Alleviating oxidative stress is therefore a crucial approach for blood glucose control. Astragaloside IV activates the JNK/Nrf2 signaling pathway to mitigate oxidative stress and restore cellular homeostasis (20). Ursolic acid scavenges ROS and attenuates diabetic damage (26), specifically by inhibiting the JNK signaling pathway to alleviate oxidative stress and protect islet cells (25). Naringin activates the Nrf2 signaling pathway to achieve antioxidant effects (141). YZ-2, a polysaccharide isolated from *Polygonatum odoratum* (Mill.) Druce, reduces oxidative stress and alleviates insulin resistance (171). FMP, a polysaccharide isolated from *Morus alba* L., suppresses oxidative stress levels by inhibiting the activation of the NF-κB pathway, thereby mitigating insulin resistance (172). GPS, a polysaccharide derived from *Panax ginseng* C.A. Mey, activates the PGC-1*α* pathway to improve the antioxidant defense system (147). Superoxide dismutase (SOD) and catalase (CAT) are key indicators of oxidative status; DTP enhances SOD and CAT activity, collectively strengthening the body’s antioxidant capacity (173).

### 4.6 Inflammation

The production of inflammatory cytokines (such as TNF-α and IL-6) can induce insulin resistance and worsen the condition of diabetic patients (170, 174). Genipin alleviates insulin resistance in diabetic rats by downregulating the gene expression of TNF-α and IL-6 (67). Ursolic acid inhibits the production of inflammatory cytokines and protects islet cells (175, 176). Ganoderic acid reduces the production of inflammatory factors in diabetic mice, leading to decreased blood glucose (122). Ginsenoside Rg5 suppresses the activation of the JNK/IRS-1 signaling pathway in diabetic mice, reduces inflammatory cytokine production, and consequently alleviates diabetic symptoms (177). Ginsenoside Rc protects islet cells from inflammatory cytokine damage by inhibiting the JNK/IKKβ/NF-κB signaling pathway. STAT3 is a downstream target of IL-6, and SHP-1 regulates STAT3 expression. Calycosin inhibits RNF38 expression, thereby upregulating SHP-1 activity and suppressing STAT3. This further reduces inflammatory factor production and restores islet cell function (178). LXA4 and LXB4 are endogenous anti-inflammatory mediators that antagonize inflammatory cytokines like IL-1, IL-6, IL-8, and TNF-*α*. Neferine increases the concentrations of LXA4 and LXB4, leading to decreased expression of inflammatory cytokines and alleviation of diabetes and its complications in diabetic rats (47). Furthermore, advanced glycation end products (AGEs) are byproducts of non-enzymatic glycation that accumulate in diabetic patients, inducing inflammatory responses. Four flavonoids isolated from Polygonatum kingianum Coll. et Hemsl. – Nicotiflorin, Narcissoside, kaempferol-3-O-(2″-O-*β*-d-glucopyranosyl)-β-d-glucopyranoside, and kaempferol-3-O-α-(6′″-p-coumaroylglucosyl-β-1,2-rhamnoside) – are potent AGEs inhibitors (35). Additionally, various active compounds including Morroniside, FCP-2-1, and Catechol also exhibit AGEs inhibitory effects (35, 81, 179). Glycyrrhizic acid blocks the production of the AGEs receptor HMGB1, thereby lowering AGEs concentration and exerting hypoglycemic effects (180). Conversely, Glycyrrhetinic Acid reduces AGEs activity and regulates blood glucose by increasing the concentration of sRAGE, a competitive binding protein for AGEs (23).

### 4.7 Lipid metabolism

Dysregulated lipid metabolism, such as elevated free fatty acids (FFAs), can induce insulin resistance and impede blood glucose reduction (181). Conversely, optimizing lipid metabolism—for instance, by promoting fatty acid oxidation or increasing adiponectin secretion—significantly improves insulin sensitivity and aids blood glucose control. Protodioscin modulates lipid metabolism and alleviates diabetes by increasing adiponectin concentration, activating the AMPK signaling pathway, and upregulating malonyl-CoA expression (182). Ganoderic acid modulates lipid metabolism by downregulating PPAR-*α* (122). Ginsenoside Rg3 activates the adiponectin pathway to regulate lipid metabolism, thereby achieving hypoglycemic effects (129). Pseudoginsenoside F11 activates PPAR-*γ*, increases adiponectin secretion, and modulates lipid metabolism (183). PECG, a polysaccharide derived from *Gallus gallus domesticus* Brisson, alleviates blood glucose issues in diabetic mice by reducing total cholesterol (TC) and triglyceride (TG) levels (184). Furthermore, polysaccharide POP from *Polygonatum odoratum* (Mill.) Druce modulates cellular lipid metabolism and regulates blood glucose balance by upregulating Nrf2-mediated HO-1 expression via the PI3K/AKT pathway (185).

### 4.8 Promote GLP-1 secretion

Glucagon-like peptide-1 (GLP-1) is a peptide hormone secreted primarily by intestinal cells located in the ileum and colon (186). It possesses the ability to promote insulin secretion from pancreatic *β*-cells and suppress appetite. Polysaccharide AMP from *Astragalus membranaceus* (Fisch.) Bge. achieves hypoglycemic effects by promoting GPCR43 expression via the gut microbiota, thereby stimulating GLP-1 secretion (79). 6-Gingerol activates the AMPK pathway, leading to increased GLP-1 expression and consequent improvement in blood glucose levels (187). Administration of phellopterin in db/db mice activates GPR119, resulting in GLP-1 secretion and blood glucose reduction (55). Puerarin activates the Wnt signaling pathway, thereby stimulating GLP-1 secretion and repairing pancreatic β-cells to promote insulin release (188). Ursolic acid promotes GLP-1 secretion by activating the bile acid receptor TGR5 (189). Ginsenoside Rg3 activates the sweet taste receptor (T1R2/T1R3), promoting GLP-1 secretion to achieve hypoglycemic effects (190). Ginsenoside CK stimulates GLP-1 secretion by inhibiting the ROCK signaling pathway (191).

### 4.9 Regulating intestinal microbiota

#### 4.9.1 Intestinal microbiota abundance

**Figure 9:**
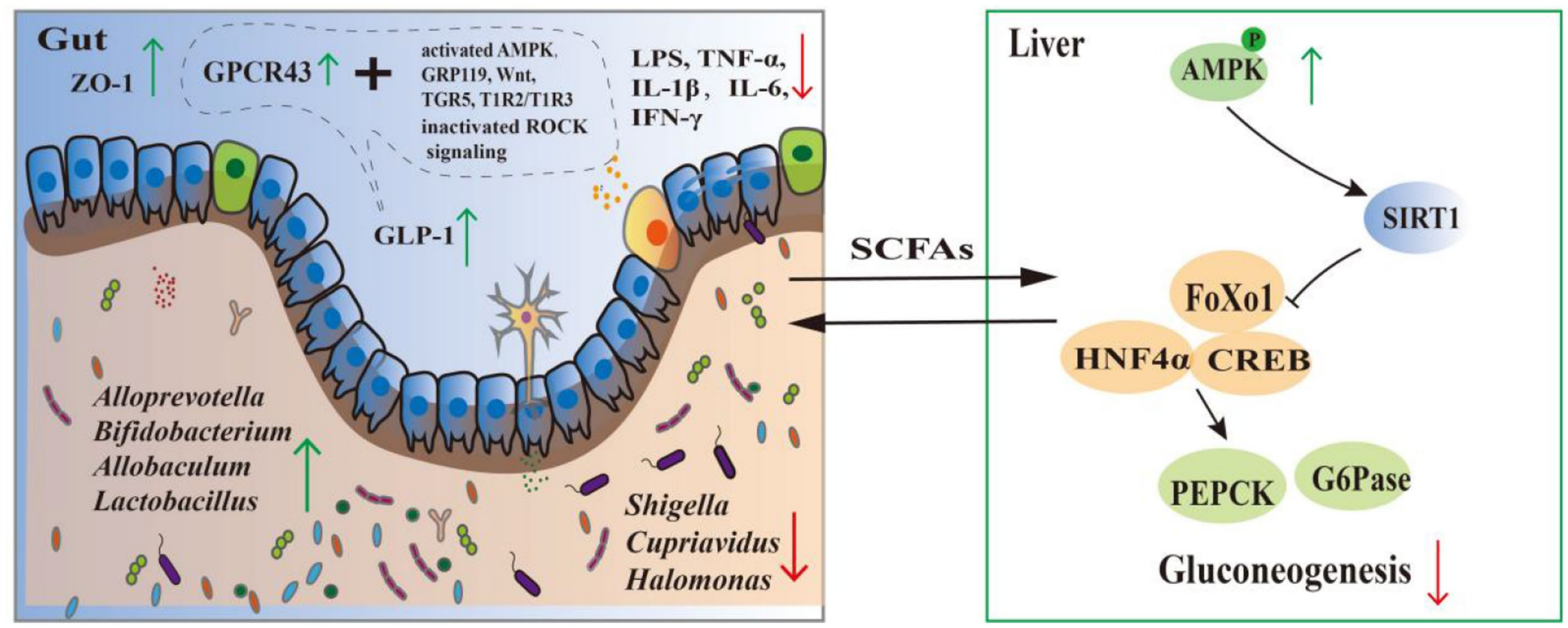
Intestinal hypoglycemic mechanism of medicine-food homology foods bioactives. “→” Represents the normal metabolic pathway process and “T” represents the inhibition of that metabolic process.

The gut microbiota plays a crucial role in maintaining microbial homeostasis and overall health (192). Bioactive substances derived from medicine-food homology foods can increase the diversity of gut microbiota and beneficial bacteria while reducing harmful bacterial populations in diabetic model mice, thereby regulating gut microecological balance and alleviating diabetes and its complications. Modulating the gut microbial community is also recognized as an effective strategy for improving glucose metabolic homeostasis. Ganoderic acid alters the abundance of various bacterial taxa, including *Bacteroidetes, Bifidobacteriales, Burkholderiales, Campylobacterales, Clostridiales, Coriobacteriales, Desulfovibrionales, Enterobacterales, Erysipelotrichales, Lactobacillales, Selenomonadales*, and *Verrucomicrobiales*. Ginsenoside Rg5 increases the abundance of *Clostridium clusters XIVa, XVIII*, and *IV*, which leads to decreased levels of inflammatory factors such as TNF-*α*, IL-6, and IL-1β, consequently alleviating diabetic symptoms (177). Nobiletin elevates the abundance of anti-diabetic probiotics such as *Alloprevotella, Desulfovibrio, Desulfovibrio piger, and Parabacteroides goldsteinii*, mitigating diabetic manifestations (161). GLP, a polysaccharide from Ganoderma lucidum (Leyss. ex Fr.) Karst., increases the abundance of beneficial anti-diabetic bacteria like *Lactococcus, Blautia,* and *Dehalobacterium*, while reducing the abundance of pathogenic bacteria such as *Ruminococcus, Coprococcus, and Aerococcus* (193). FMP, isolated from *Morus alba* L., increases the abundance of bacteria like *Allobaculum* and *Bifidobacterium*, which possess the ability to reduce gut-derived endotoxin permeation and alleviate plasma endotoxemia. This reduces endotoxin-induced intestinal mucosal inflammation, promotes mucosal repair, and ultimately alleviates systemic inflammation to regulate diabetes. Furthermore, FMP reduces the abundance of Shigella, thereby decreasing the production of intestinal inflammatory factors (172). APP significantly increases the abundance of *Allobaculum* and *Lactobacillus* while decreasing the abundance of *Cupriavidus, Halomonas,* and *Shigella*. *Lactobacillus* enhances the body’s antioxidant capacity and immune system function, improves intestinal motility, and antagonizes hyperglycemia (194). Figure 9 illustrates the glucose-lowering mechanisms of medicine-food homology foods substances on the gut.

#### 4.9.2 Metabolites

Short-chain fatty acids (SCFAs), core metabolites produced by gut microbiota through dietary fiber fermentation, play a pivotal role in regulating glucose homeostasis (195). MFP increases SCFA levels by elevating the abundance of *Prevotella, Bacteroides, Lactobacillus,* and *Bifidobacterium,* thereby lowering blood glucose in mice (196). Astragaloside IV not only modulates blood glucose through activating multiple pathways but also regulates the gut microbiota, increasing the abundance of SCFA-producing bacteria such as *Rikenella, Alistipes,* and *Odoribacter.* This subsequently activates the AMPK signaling pathway to suppress gluconeogenesis and ameliorate T2DM-related symptoms (19). WHBP modulates the composition of the gut microbiota and its metabolite SCFAs, thereby activating the HPA axis. This alleviates systemic oxidative stress and contributes to blood glucose regulation (197). LBP increases SCFA concentrations in the mouse intestine by boosting the abundance of SCFA-producing bacteria like *Allobaculum.* This upregulates ZO-1 expression and reduces levels of inflammatory factors such as LPS, TNF-*α*, IL-6, and IFN-*γ*, consequently inhibiting systemic inflammation and alleviating diabetes (198). CSP stimulates SCFA production by *Lactobacillus, Akkermansia, Bacteroides,* and *Bifidobacterium,* leading to increased IGF1 protein expression. This activates the PI3K/AKT signaling pathway to regulate glucose metabolism (199).

## 5 Potential side effects

As special resource with both nutritional and medicinal value, food and medicine homology resources exhibits high security compared to traditional therapeutic drugs. However, for natural products from food and medicine homology resources, some potential side effects were also observed as a result of high doses or long-term administration. A scientific safety threshold system needs to be established through systematic toxicology research.

Intravenous administration of Astroloside IVmore than 1 mg/kg may result in maternal toxicity; it over 0.5 mg/kg may cause fetal toxicity, pregnant women or lactating women should use it with caution (200). Ursolic acid can enhance the activity of thrombin and promote the formation of arterial plaques, it is not conducive to consumption by patients with cardiovascular diseases (201); Mild nausea, vomiting, diarrhea and abdominal distension, etc. were observed in animal experiments after consuming Ginsenoside Rb2 with high doses (≥50 mg/kg) (202). Formononetin can cause transient vomiting and vascular irritation (may recover on its own) with over 300 mg/kg · d, but it does not lead to death or severe organ damage (203). Kaempferol can inhibit platelet activity, and may increase the risk of bleeding when it was used combination with anticoagulants or antiplatelet drugs (204); After taking Puerarin, temporary bloating, stomach discomfort, and heartburn may occur (205). The potential side effects of medicine for homology materials compounds are shown in Supplementary Table S10 (Supplementary Table S10: Potential side effects of certain medicine-food homology foods or their components are listed, such as the risks of astragaloside IV for pregnant women and gastrointestinal discomfort caused by soy isoflavones, highlighting the need to pay attention to dosage and target populations).

## 6 Applications in glycemic management functional foods

**Figure 10:**
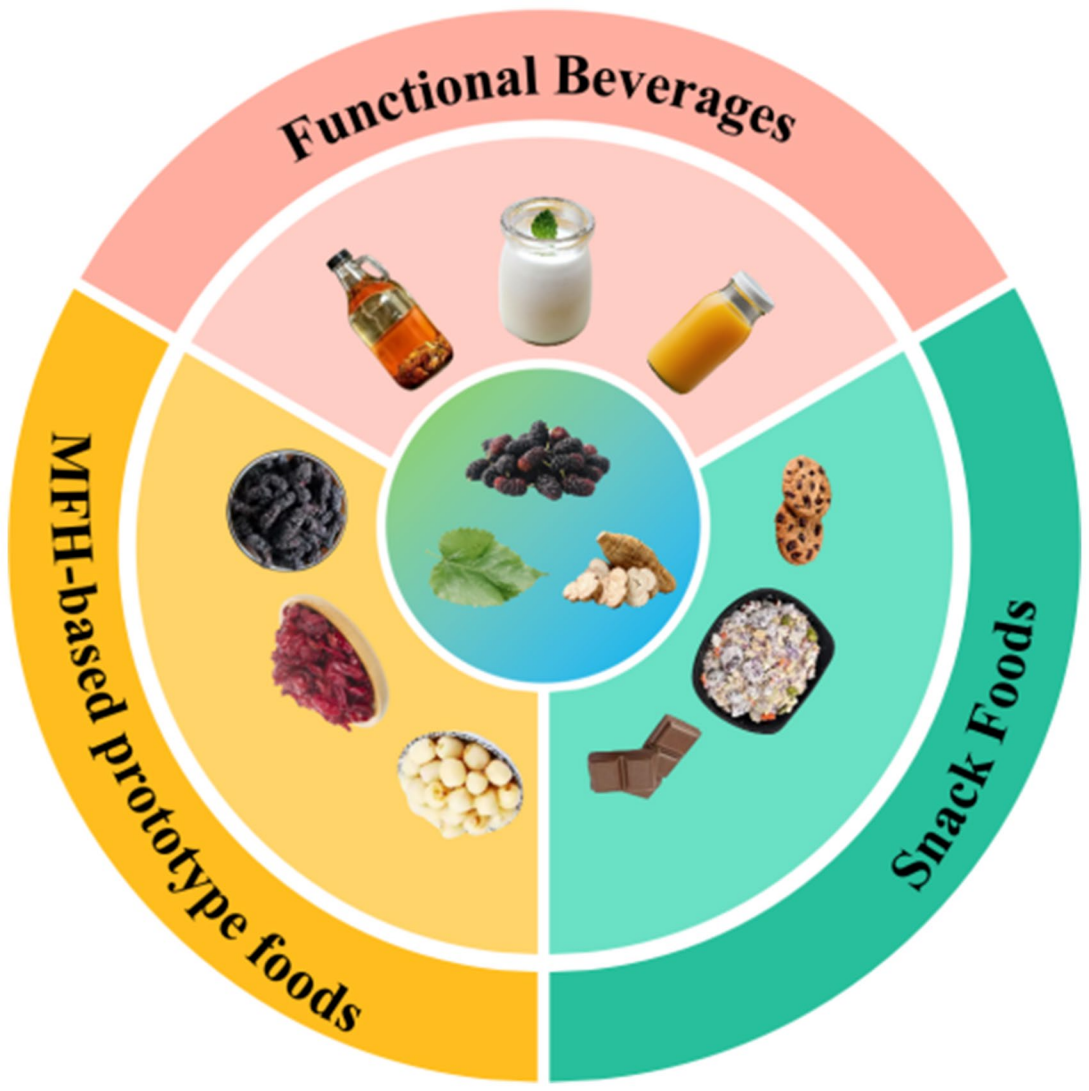
Classification of medicine-food homology foods products.

Medicine-food homology foods have been strategically leveraged to develop consumer products that combine distinctive sensory attributes with clinically validated anti-diabetic properties. These innovations are systematically categorized into three functional classes (206) (Figure 10): (1) medicine-food homology (MFH)-based prototype foods, bioactive compounds from materials were preserved through dehydration technologies, such as hawthorn chips (*Crataegus pinnatifida*), cornelian cherry preserves (*Cornus officinalis*), and dehydrated lotus seeds (*Nelumbo nucifera*); (2) functional beverages, synergistic formulations were employed, such as kombucha-fermented goji berry vinegar drinks (*Lycium barbarum*) and dandelion health tonics (*Taraxacum officinale*); and (3) snack foods, bioactive compounds were integrated into convenience foods, including coix seed milk (*Coix lacryma-jobi*), oilseed meal multigrain biscuits, *Astragalus membranaceus*-fortified dietary fiber crackers, and sugarcane bagasse fiber snacks. These also suggests that people’s increasingly strong awareness of active health for preventing diseases on chronic metabolic disorders, such as diabetes and obesity. Comprehensive product specifications are provided in Supplementary Table S11 (Supplementary Table S11: Glucose-lowering products developed from medicine-food homology foods, categorized into snack foods and functional beverages).

## 7 Conclusion and prospects

Diabetes mellitus, as a global public health challenge, has witnessed escalating prevention and treatment demands. Traditional medicine-food homology foods characterized by their dual nutritional and pharmacological functions, and low toxicity, have emerged as promising candidates for blood glucose regulation. Previous studies revealed that traditional herbs, such as *Astragalus membranaceus*, *Poria cocos*, *Glycyrrhiza uralensis*, and *Pueraria lobata,* contain abundant bioactive components including triterpenoids, flavonoids, alkaloids, phenylpropanoid, iridoid and polysaccharide (19, 79, 107, 207–210). Additionally, the C-3 and C-28 groups of triterpenoids and their glycosides enhance glucose uptake by binding to PPARγ (94, 95), while the C-17 group activates the AMPK pathway to exert hypoglycemic effects (96, 97). For flavonoids, the hydroxyl group at the C-6 position of the A-ring is recognized as a key functional group for hypoglycemic activity, and the C2-C3 double bond stabilizes hydroxyl conformations, acticating the AMPK pathway (98, 99). Alkaloids’ polar groups (hydroxyl and amino groups) and planar fused rings play pivotal roles in blood glucose reduction (103–105). The hypoglycemic activity of phenylpropanoids is primarily determined by the number and position of phenolic hydroxyl groups, with the ortho-dihydroxy structure activating the AMPK signaling pathway and the *α*, *β*-unsaturated carbonyl terminus of the propenoic side chain modulating enzymes like α-glucosidase and PTP1B, while oxygenated substitutions at C6/C7 are also key regulators (106–109); iridoids exert their hypoglycemic effects manifested in structural features including the epoxy-containing cyclopentane scaffold, substituents at C4, phenolic hydroxyl groups, and unsaturation status (69, 71, 110). Polysaccharides with hypoglycemic activity typically exhibit a relative molecular weight ranging from 10 to 50 kDa, consisted of arabinose, galactose, glucose and xylose, with 1 → 3 and 1 → 4 glycosidic bonds (111–118). These compounds exert significant antidiabetic effects through multiple mechanisms: enhancing insulin secretion, ameliorating oxidative stress, modulating glucose metabolism, and regulating gut microbiota. However, their structure–function relationships, molecular targets, and underlying mechanisms are still unclear. On the other hand, medicine-food homology foods can be utilized to develop MFH-based prototype foods, functional beverages, and snack foods. This allows for the incorporation of foods with hypoglycemic effects into daily life. This provide diverse and health dietary choices for diabetes prevention and management. These reveals that human’s notion transformation from passive health to proactive prevention in chronic metabolic diseases such as diabetes.

Although medicine-food homology foods possess rich dietary applications and pharmacological effects, however, their development of functional foods is still in the early stage. In the future, it is essential to strengthen the connection between fundamental research and the food industry to accelerate this process of relative food development. Based on the immature structure–activity relationships between active compounds from medicine-food homology foods and hypoglycemic targets, the exact targets and detailed molecular mechanisms of these components should be further explored and clarified at the cellular, animal, and clinical levels, with the aid of emerging technologies and methods. It seems that digging novel anti-diabetic targets using these components as tools is a future hot topic, this can accelerate the development of new drugs and promote the creation of functional foods on T2DM treatment and prevention. Additionally, accelerating the creation of palatable hypoglycemic foods using these resources or incorporating varieties with suboptimal flavor profiles as ingredients represents a vital approach to promoting medicinal-edible functional foods. Furthermore, compiling personalized data on diabetic patients’ etiology, symptoms, and dietary preferences to construct a medicinal-edible product database, enabling tailored functional food supplementation plans, this offers a novel strategy for diabetic patients in different periods, with significant differences in environmental factors and individual differences.
