# Supplementary material for: Transformation from passive health to proactive prevention: research progress on glucose-lowering components and its mechanism of food and medicine homology resources
Source: Front Nutr. 2026 Jan 5;12:1681916. doi: 10.3389/fnut.2025.1681916 (PMC12812749; doi:10.3389/fnut.2025.1681916)
Supplement: Supplementary file 1 [file Table_1.docx]

Supplementary Material

Table S1 food and medicine homology foods with hypoglycemic effect

| Name | Source | Distribution | Name | Source | Distribution |
| --- | --- | --- | --- | --- | --- |
| *Astrogali radix* | *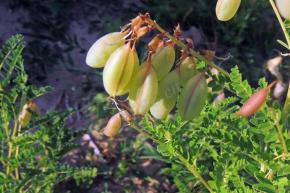*  *Astragalus membranaceus* (Fisch.) Bge. var. mongholicus (Bge.) Hsiao | China,Russia, North Korea, Mongolia, etc | *Lablab semen album* | *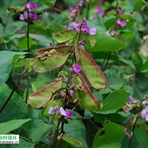*  *Dolichos lablab* L. | China, Japan, India, Indonesia, etc |
| *Allii macrostemonis bulbus* | *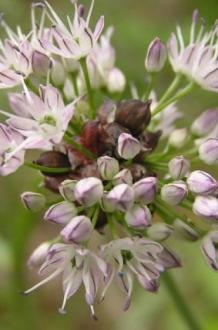*  *Allium chinense* G. Don | Russia, North Korea, Japan, etc | *Lilii bulbus* | *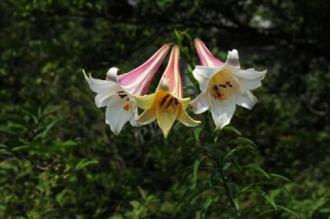*  *Lilium brownii* F. E. Brown ex Miellez | China, Japan , North Korea, etc |
| *Amomi fructus* | *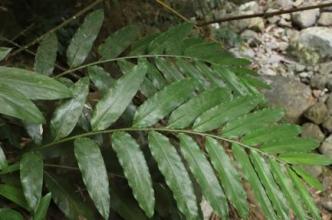*  *Amomum villosum* Lour. | China,Vietnam, Thailand, Myanmar, Indonesia, etc | *Lonicerae japonicae* | *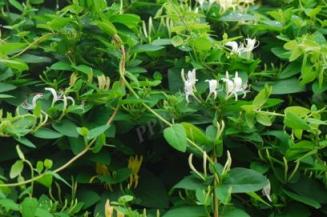*  *Lonicera japonica* Thunb. | China, Japan , North Korea, etc |
| *Angelicae dahuricae Radix* | *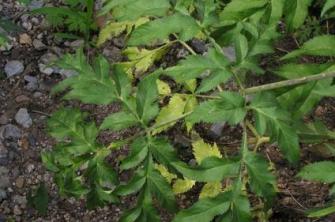*  *Angelica dahurica* Bentham et Hooker f. | China, Japan, North Korea,Russia, etc | *Lycii fructus* | *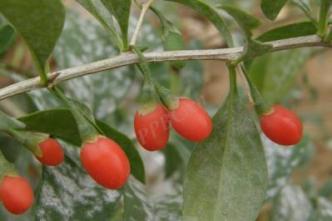*  *Lycium barbarum* L. | China, Japan, North Korea, European countries |
| *Angelicae Sinensis radix* | *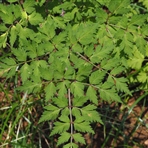*  Angelica sinensis (Oliv.) Diels | China, Japan, South Korea, etc | *Menthae haplocalycis herba* | *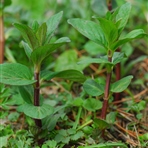*  *Mentha haplocalyx* Briq. | Asia and North America |
| *Cannabis fructus* | *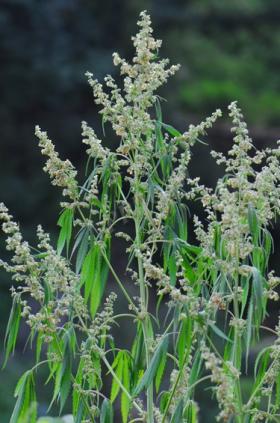*  *Cannabis sativa* L. | All countries have | *Mori folium* | *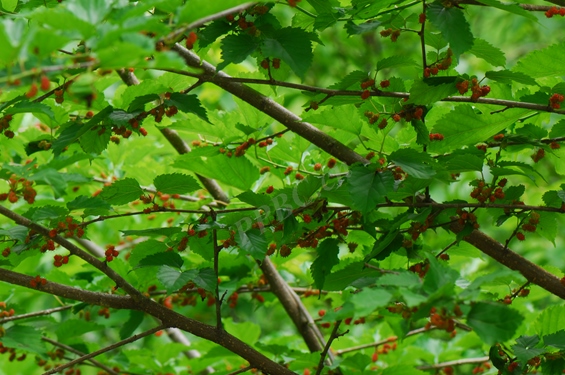*  *Morus alba* L. | China, Japan, Korean Peninsula, Russia, Central Asia, continental Europe, United States, Mexico, South Africa |
| *Caryophylli flos* | *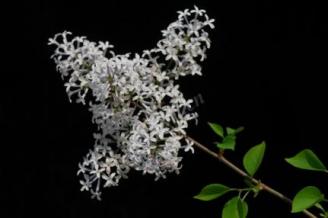*  *Eugenia caryophyllata* Thunb. | *China, Malaysia, Indonesia*, etc | *Mori fructus* | *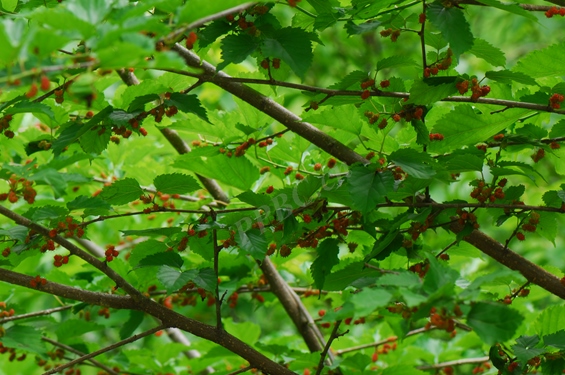*  *Morus alba* L. | China, Japan, Korean Peninsula, Russia, Central Asia, continental Europe, United States, Mexico, South Africa |
| *Cassiae semen* | *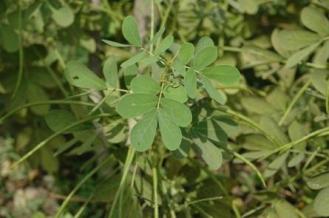*  *Cassia obtusifolia* L. | *China, North America*, etc | *Mume fructus* | *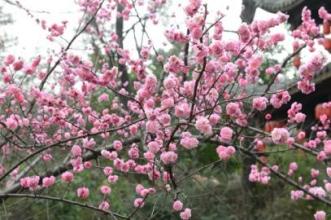*  *Prunus mume* (Sieh.) Sieb. et Zucc. | China, Japan,Korea, etc |
| *Chrysanthemi flos* | *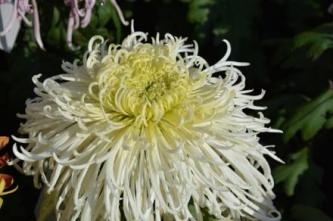*  *Chrysanthemum indicum* L. | *China, Japan*, etc | *Myristicae semen* | *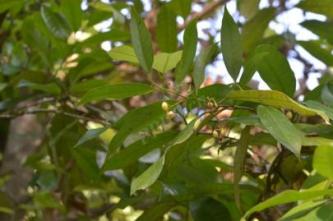*  *Myristica fragrans* Houtt. | East Asia, Southeast Asia, Oceanian countries |
| *Cinnamomi cortex* | *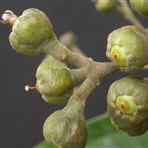*  *Cinnamomum cassia* (L.) D. Don | China, Vietnam, Malaysia, etc | *Nelumbinis semen* | *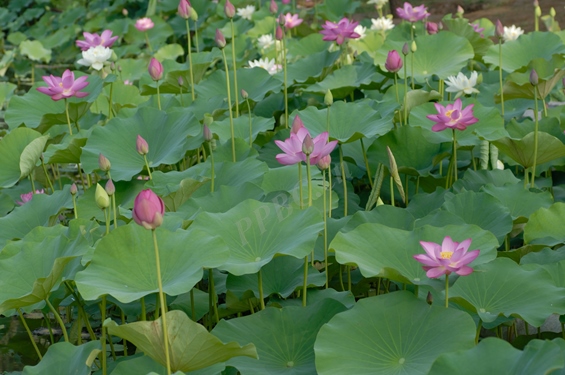*  *Nelumbo nucifera* Gaertn. | Central Asia, West Asia, North America, India, China, Japan, etc |
| *Cirsii herba* | *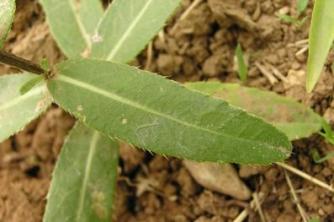*  *Cirsium setosum* (Willd.) MB. | China, Russia, Ukraine, Mongolia, etc | *Panacis quinquefolii radix* | *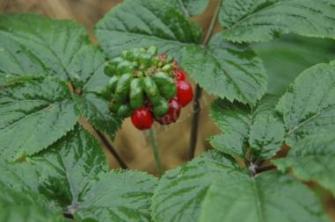*  *Panax quinquefolium* L. | China, North American countries, etc |
| *Cistanche deserticola ma* | *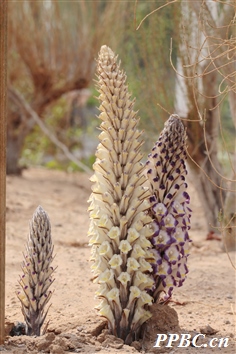*  *Cistanche deserticola* Ma | China, Russia, Ukraine, Mongolia, etc | *Perillae fructus* | *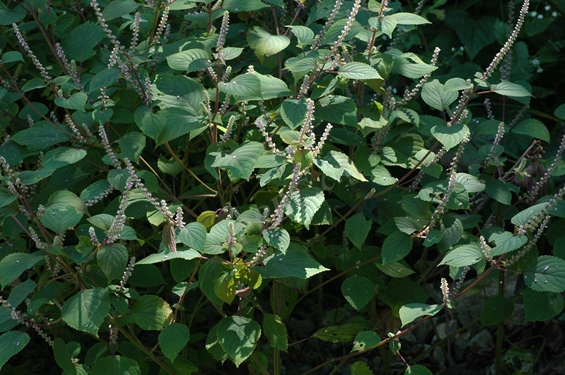*  *Perilla frutescens*(L.) Britt. | China, Bhutan, India, Vietnam, Indonesia, Japan, North Korea, etc |
| *Citri reticulatae pericarpium* | *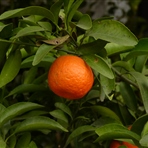*  *Citrus reticulata* Blanco | All countries have | *Persicae semen* | *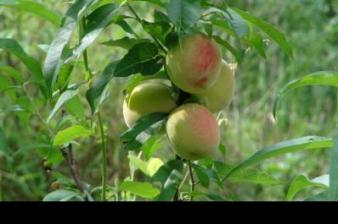*  *Prunus persica* (L.) Batsch | China, Spain, Italy, Turkey, Iran, Greece, etc |
| *Citri sarcodactylis fructus* | *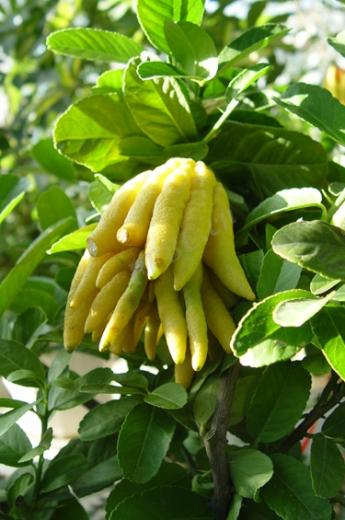*  *Citrus medica* L. var. sarcodactylis Swingle | China, Japan, India, etc | *Perilla frutescens* | *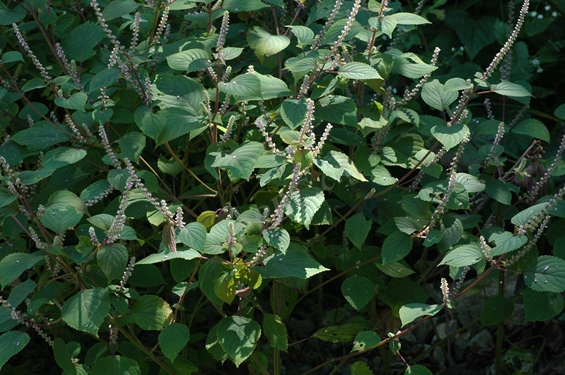*  *Perilla frutescens*(L.) Britt. | China, Bhutan, India, Vietnam, Indonesia, Japan, North Korea, etc |
| *Codonopsis radix* | *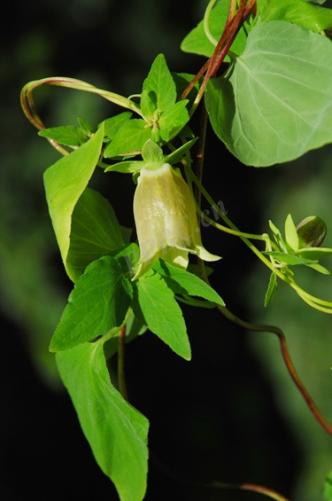*  *Codonopsis pilosula* (Franch.) Nannf. | China, Russia, Mongolia, etc | *Platycodonis radix* | *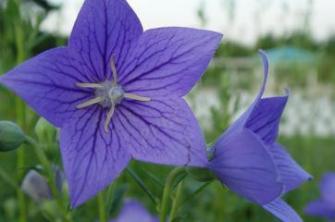*  *Platycodon grandiflorum*（Jacq.）A.DC. | China, Japan, South Korea, Russia, North Korea, Mongolia, Bhutan, Vietnam, etc |
| *Coicis semen* | *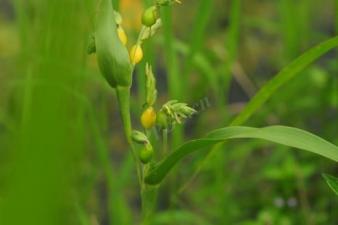*  *Coix lacryma-jobi* L.var. ma-yuen (Roman.) Stapf | China, Japan, North Korea, South Korea, etc | *Pogostemonis herba* | *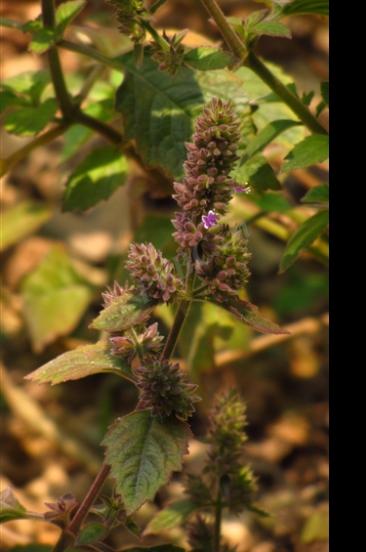*  *Pogostemon cablin* (Blanco) Benth. | China, India, Sri Lanka, Malaysia, Indonesia, Philippines, etc |
| *Cornifructus* | *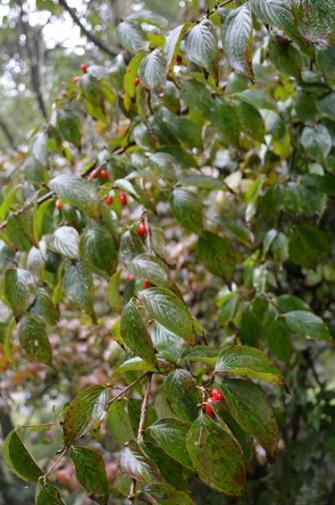*  *Cornus officinalis* Sieb.et Zucc. | China, Japan, North Korea, South Korea, etc | *Polygonati odorati rhizoma* | *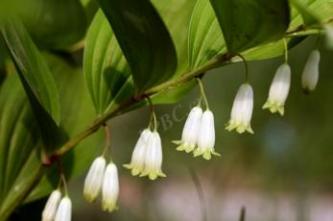*  *Polygonatum odoratum* (Mill.) Druce | China, North Korea, Mongolia, Russia, etc |
| *Crataegi fructus* | *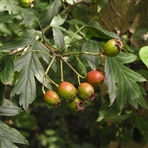*  *Crataegus pinnatifida* Bge. | All countries have | *Polygonati rhizoma* | *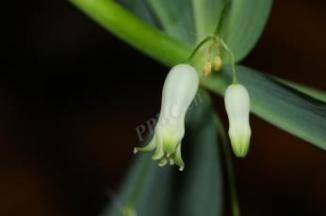*  *Polygonatum kingianum* Coll. et Hemsl. | Temperate region of Eurasia |
| *Curcumae longae rhizoma* | *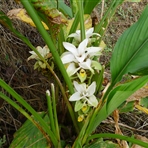*  *Curcuma longa* L. rhizomes | China, India, Vietnam, etc | *Poria cocos* | *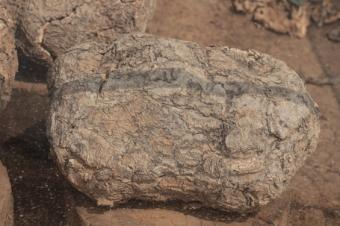*  *Poria cocos* (Schw.) Wolf. | China, Japan, Australia, the Americas and Southeast Asian countries |
| *Dioscoreae rhizoma* | *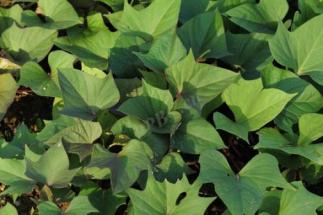*  *Dioscorea opposita* Thunb. | All countries have | *Portulacae herba* | *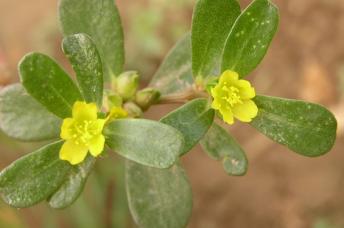*  *Portulaca oleracea* L. | China, Germany, Spain, Sweden, Netherlands, etc |
| *Euryales semen* | *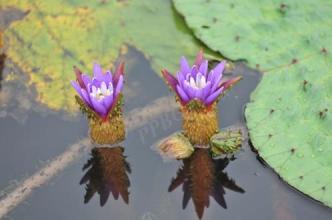*  *Euryale ferox* salisb. | China, Vietnam, Russia, Japan, India, North Korea, etc | *Puerariae lobatae radix* | *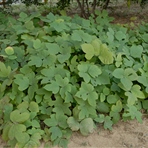*  *Pueraria lobata*（Willd.）Ohwi | China, Vietnam, Laos, Japan, Australia, etc |
| *Folium nelumbinis* | *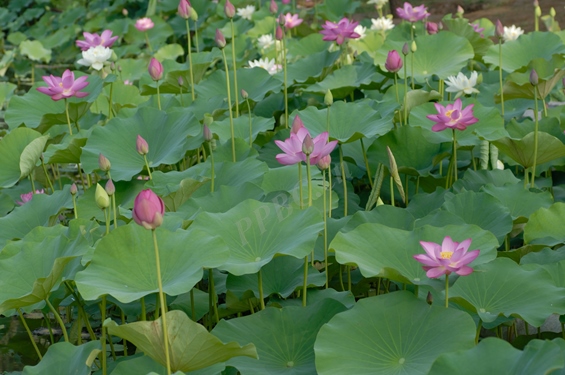*  *Nelumbo nucifera* Gaertn. | Central Asia, West Asia, North America, India, China, Japan, etc | *Rubi fructus* | *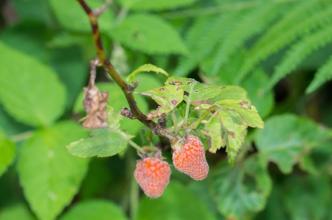*  *Rubus chingii* Hu | Asia, North America, Europe |
| *Fructus jujubae* | *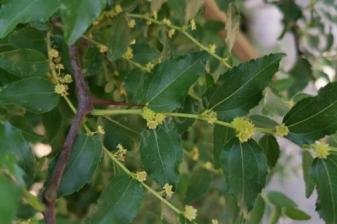*  *Ziziphus jujuba* Mill. var. spinosa (Bunge) Hu ex H. F. Chou | China, Japan, Central Asia, etc | *Semen armeniacae amarum* | *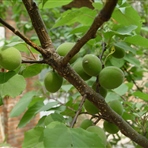*  *Semen armeniacae* Amarum. | Asian and Mediterranean Countries |
| *Flos sophorae immaturus* | *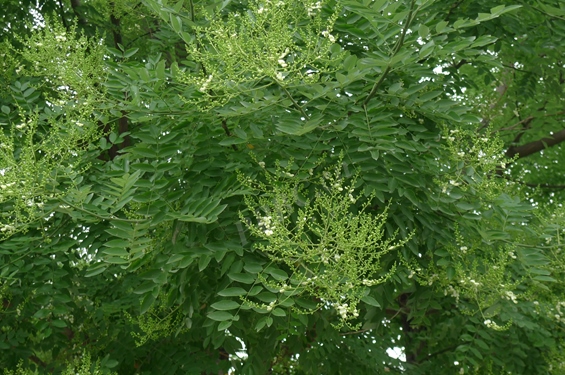*  *Sophora japonica* L. | China, Vietnam, Japan, North Korea and European and American countries, etc | *Sesami semen nigrum* | *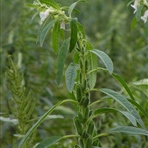*  *Sesamum indicum* L. | Asia, Africa, central and South America |
| *Galli Gigerii Endothelium Coreneum* | *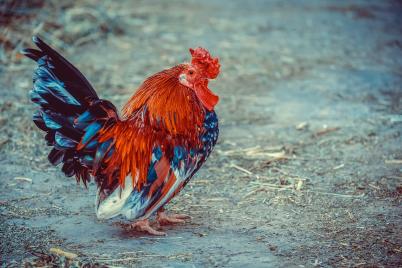*  *Gallus gallus domesticus* Brisson | All countries have | *Siraitiae fructus* | *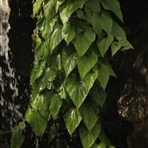*  *Siraitia grosvenorii* (Swingle) C. Jeffrey ex Lu et Z. Y. Zhang | China, Brazil, Congo, etc |
| *Ganoderma* | *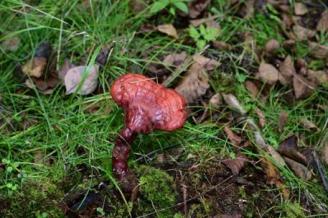*  *Ganoderma lucidum* (Leyss.ex Fr.) Karst. | China, South Korea, Japan and other Asian countries | *Sojae semen praeparatum* | *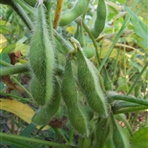*  *Glycine max* (L.) Merr. | South America, North America and Asia |
| *Gardeniae fructus* | *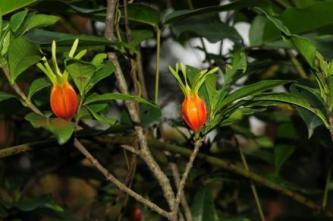*  *Gardenia jasminoides* ellis | China, Japan, North Korea, Vietnam, Laos, Cambodia, India, Nepal, Pakistan, Pacific Islands and Northern America | *Sophorae flos* | *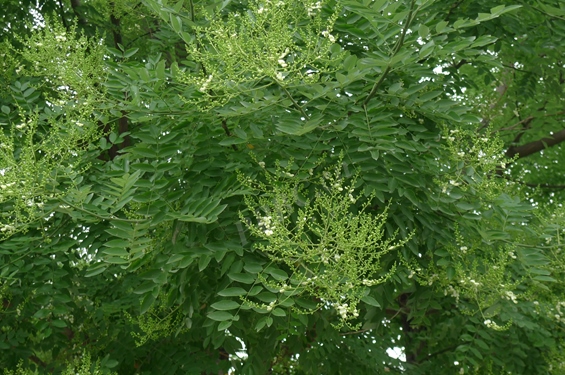*  *Sophora japonica* L. | China, Vietnam, Japan, North Korea and European and American countries, etc |
| *Gastrodiae rhizoma* | *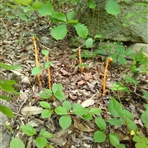*  *Gastrodia elata* Bl. | Asia and Africa | *Taraxaci herba* | *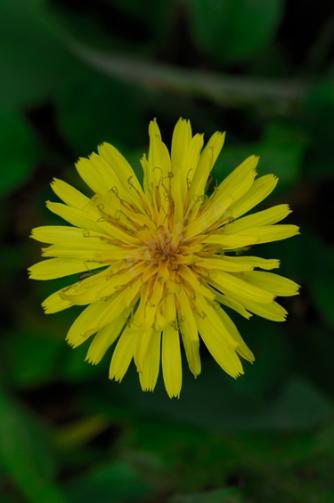*  *Taraxacum mongolicum* Hand. -Mazz. | China, Vietnam, Japan, North Korea, etc |
| *Ginseng radix et rhizoma* | *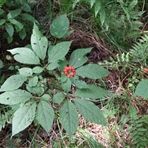*  *Panax ginseng* C.A. Mey | China, North Korea, South Korea, Japan, Russia, etc | *Tsaoko fructus* | *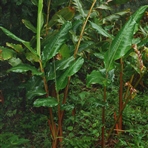*  *Amomum villosum* Lour. | China, Vietnam, Laos, etc |
| Glycyrrhizae radix et rhizom | *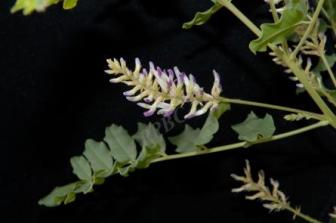*  *Glycyrrhiza uralensis* Fisch | Northern China, Mongolia, Russia, Kazakhstan, Pakistan, etc | *Zanthoxyli pericarpium* | *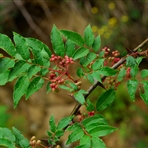*  *Zanthoxylum bungeanum* Maxim. | China, Korea, Japan, etc |
| *Hordei fructus germinatus* | *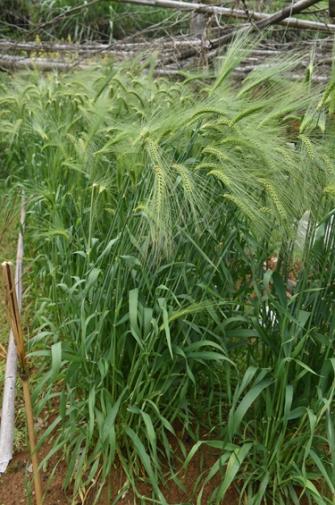*  *Hordeum vulgare* L. | All countries have | *Zingiberis rhizoma recens* | *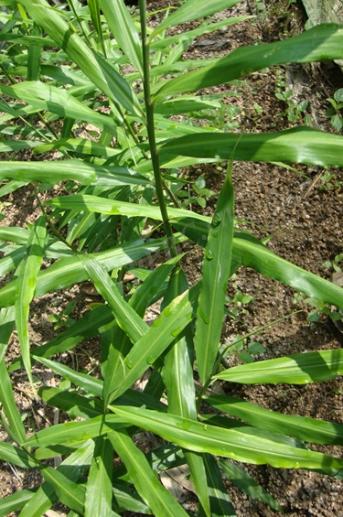*  *Zingiber officinale* (Willd.) Rosc. | Asia and Africa |
| *Houttuyniae herba* | *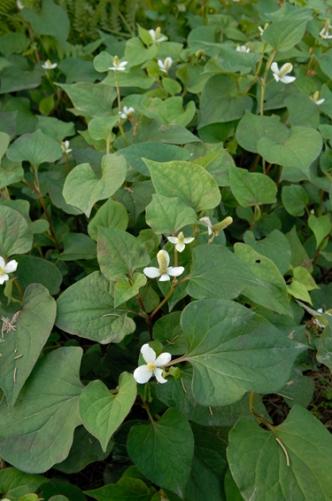*  *Houttuynia cordata* Thunb. | China, Japan, South Korea, Vietnam, etc | *Ziziphi spinosae semen* | *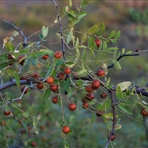*  *Ziziphus jujuba* Mill. var. spinosa (Bunge) Hu ex H. F. Chou | China, North Korea,Russia, etc |

Note: For table 1, pictures were obtained from iPlant and Baidu Baike.

Plant reprinted with permission from PPBC (Plant Photo Bank of China),licensed under CC BY-NC-ND, <http://ppbc.iplant.cn.>

*Galli Gigerii Endothelium Coreneum* reprinted with permission from pixabay by Couleur,licensed under CC0 (Public Domain Dedication),https://pixabay.com/zh/photos/chicken-animal-poultry-farm-3741129/

Table S2 The frequency of use of food and medicine homology foods on regulating blood glucose

| Materials | Frequency | Materials | Frequency |
| --- | --- | --- | --- |
| *Astragali radix* | 236 | *Pogostemonis herba* | 6 |
| Poria cocos | 154 | *Gastrodiae rhizoma* | 6 |
| *Glycyrrhizae radix et rhizom* | 128 | *Semen armeniacae amarum* | 5 |
| *Puerariae lobatae radix* | 110 | *Ziziphi spinosae semen* | 5 |
| *Dioscoreae rhizoma* | 97 | *Curcumae longae rhizoma* | 5 |
| *Angelicae sinensis radix* | 89 | *Panacis quinquefolii radix* | 5 |
| *Citri reticulatae Pericarpium* | 63 | *Nelumbinis semen* | 4 |
| *Codonopsis radix* | 51 | *Chrysanthemi flos* | 4 |
| *Zingiberis rhizoma recens* | 49 | *Allii macrostemonis bulbus* | 3 |
| *Crataegi fructus* | 45 | *Portulacae herba* | 3 |
| *Cornifructus* | 43 | *Citri sarcodactylis fructus* | 3 |
| *Lycii fructus* | 39 | *Zanthoxyli pericarpium* | 3 |
| *Polygonati rhizoma* | 37 | *Fructus alpinae oxyphyllae* | 3 |
| *Coicis semen* | 26 | *Cirsii herba* | 2 |
| *Folium nelumbinis* | 24 | *Cannabis fructus* | 2 |
| *Fructus jujubae* | 21 | *Lilii bulbus* | 2 |
| *Cinnamomi cortex* | 19 | *Myristicae semen* | 2 |
| *Polygonati odorati rhizoma* | 16 | *Mori fructus* | 2 |
| *Cassiae semen* | 15 | *Lophatheri herba* | 2 |
| *Mori folium* | 15 | *Perilla frutescens* | 2 |
| *Euryales semen* | 12 | *Tsaoko fructus* | 2 |
| *Amomi fructus* | 12 | *Angelicae dahuricae Radix* | 1 |
| *Galli gigerii Endothelium co* | 11 | *Siraitiae fructus* | 1 |
| *Lonicerae japonicae* | 9 | *Houttuyniae herba* | 1 |
| *Hordei fructus germinatus* | 8 | *Sesami semen nigrum* | 1 |
| *Taraxaci herba* | 8 | *Flos sophorae immaturus* | 1 |
| *Mume fructus* | 7 | *Sophorae flos* | 1 |
| *Gardeniae fructus* | 7 | *Phragmitis rhizoma* | 1 |
| *Lablab semen album* | 6 | *Cistanche deserticola ma* | 1 |
| *Menthae haplocalycis herba* | 6 | *Ganoderma* | 1 |
| *Rubi fructus* | 6 | *Caryophylli flos* | 1 |

Table S3 Triterpenoids from food and medicine homologyfoods and their glucose-regulating mechanisms

| NO | Name | Source | Experimental model | Potential mechanism | Ref. |
| --- | --- | --- | --- | --- | --- |
| 1 | 11-epi-Mogroside V | *Siraitia grosvenorii* (Swingle) C. Jeffrey ex Lu et Z. Y. Zhang | HepG2 cell | Improve glucose uptake | (1) |
| 2 | 11-O-Mogroside VI | *Siraitia grosvenorii* (Swingle) C. Jeffrey ex Lu et Z. Y. Zhang | HepG2 cell | Improve glucose uptake | (1) |
| 3 | 11-O-Siamenoside I | *Siraitia grosvenorii* (Swingle) C. Jeffrey ex Lu et Z. Y. Zhang | HepG2 cell | Improve glucose uptake | (1) |
| 4 | 24-Methylenecycloartanol | *Codonopsis pilosula* (Franch.) Nannf. | PNPG | Inhibition of α - glucosidase | (2) |
| 5 | 2β-Hydroxybetulinic acid 3β-oleiate | *Euryale ferox* salisb. | STZ induced diabetes rats | Protect pancreatic beta cells | (3) |
| 6 | 3-Epidehydrotumulosic acid | *Poria cocos (Schw.)* Wolf. | PNPG | Inhibition of alpha glucosidase | (4) |
| 7 | 3-O-Acetyl-16alpha-hydroxytrametenolic acid | *Poria cocos (Schw.)* Wolf. | PNPG | Inhibition of alpha glucosidase | (4) |
| 8 | 3α-Hydroxy-25-dehydroxy -24-oxomogrol | *Siraitia grosvenorii* (Swingle) C. Jeffrey ex Lu et Z. Y. Zhang | HepG2 cell | Activate AMPK | (5) |
| 9 | 3α-Hydroxymogrol | *Siraitia grosvenorii* (Swingle) C. Jeffrey ex Lu et Z. Y. Zhang | HepG2 cell | Activate AMPK | (5) |
| 10 | Astragaloside IV | *A. membranaceus* (Fisch.) Bge. | insulin resistance-HepG2 cells  STZ and HFD induced diabetes rats | Regulated intestinal flora  Activate AMPK and PI3K/AKT signaling pathways, and inhibit gluconeogenesis | (6) |
|  |  |  | STZ and HFD induced diabetes mice | Inhibition of GP and G6Pase | (7) |
|  |  |  | STZ and ALX induced diabetic ketoacidosis mice | Activation of JNK/Nrf2 signaling pathway and antioxidant stress | (8) |
|  |  |  | gestational diabetes mellitus db/+ mice | Activated Akt/PDE4B signaling pathway and promote lipolysis  Inhibition of PEPCK and G6Pase,inhibition of gluconeogenesis | (9) |
|  |  |  | C2C12 cells | Activated IRS1/Akt signaling pathway, up regulate GLUT4 expression and stimulate glucose uptake  Inhibited IKK/I κ B α/NF - κ B signaling pathway, reducing inflammatory factors and protecting islet cells | (10) |
| 11 | Chikusetsusaponin IVa | *Dolichos lablab* L. | Diabetes db/db mice | Inhibition of α - glucosidase  Protect islet beta cells | (11) |
| 12 | Daedaleanic Acid F | *Poria cocos (Schw.)* Wolf. | 3T3-L1 cell | Glucose uptake stimulating activity and insulin sensitizing activity | (12) |
| 13 | Dehydropachymic acid | *Poria cocos (Schw.)* Wolf. | 3T3-L1 cell | Glucose uptake stimulating activity and insulin sensitizing activity | (12) |
|  |  |  | PNPG | Inhibition of alpha glucosidase | (4) |
| 14 | Dehydrotrametenolic acid | *Poria cocos (Schw.)* Wolf. | 3T3-L1 cell、  *db*/*db*  mice | Activate PPAR γ | (13) |
|  |  |  | db/db mice(noninsulin-dependent diabetes mellitus) | Activate PPAR γ | (14) |
| 15 | Dehydrotumulosic acid | *Poria cocos (Schw.)* Wolf. | PNPG | Inhibition of alpha glucosidase | (4) |
| 16 | Eburicoic acid | *Poria cocos (Schw.)* Wolf. | 3T3-L1 cell | Glucose uptake stimulating activity and insulin sensitizing activity | (12) |
| 17 | Eburicoic acid acetate | *Poria cocos (Schw.)* Wolf. | 3T3-L1 cell | Glucose uptake stimulating activity and insulin sensitizing activity | (12) |
| 18 | Ganoderic acid | *Ganoderma lucidum* (Leyss.ex Fr.) Karst. | HepG2 cell  3T3-L1 cell  STZ and HFD induced diabetes mice | Inhibition of α - glucosidase  Inhibition of α - amylase  Reduce inflammatory factors and protect islet cells  Down regulation of PPAR - α and regulation of lipid metabolism  Regulating intestinal flora | (15) |
| 19 | Ginsenoside CK | *Panax ginseng* C.A. Mey | NCI-H716 cell | Inhibit RhoA/Rock/Yap signaling pathway and stimulate GLP-1 secretion | (16) |
|  |  |  | HEPG2  STZ and HFD induced diabetes mice | It can increase AMPK signaling pathway, inhibit the expression of PGC-1 α, HNF-4 α and FoxO1, inhibit PEPCK and G6Pase, and inhibit gluconeogenesis | (17) |
|  |  |  | MIN6 pancreatic β cells | Upregulation of GLUT2 expression promotes glucose uptake | (18) |
|  |  |  | STZ and HFD induced diabetes mice | Inhibition of PEPCK and G6Pase, inhibition of gluconeogenesis | (19) |
|  |  |  | HFD induced diabetes mice | Activation of PPAR γ/NF - κ B/I κ B α signaling pathway protects islet cells  Activate IRS1/PI3K signaling pathway, up regulate GLUT4 and promote glucose uptake | (20) |
|  |  |  | STZ and HFD induced diabetes rats | Activate PI3K/Akt signaling pathway, up regulate GLUT4 and promote glucose uptake | (21) |
| 20 | Ginsenoside Rb1 | *Panax ginseng* C.A. Mey | STZ and HFD induced diabetes mice | Activate PI3K/Akt/STAT3 signaling pathway, promote glycolysis | (22) |
|  |  |  | LO2  cell  STZ and HFD induced diabetes mice | Regulate 15-PGDH/PGE2/EP4 pathway and promote hepatic glycogen synthesis | (23) |
|  |  |  | *db/db mice*  3T3-L1 cell | Activate Akt pathway, up regulate GLUT4 and promote glucose uptake | (24) |
|  |  | *Panax quinquefolium* L. | PNPG | Inhibition of α - glucosidase | *(25)* |
| 21 | Ginsenoside Rb2 | *Panax ginseng* C.A. Mey | 3T3-L1 cell  [DIO mice](https://www.sciencedirect.com/topics/biochemistry-genetics-and-molecular-biology/mouse-mutant) | Activate IRS-1/PI3K/- Akt/PKB pathway, up regulate GLUT4 and promote glucose uptake  Inhibiting JNK/NF - κ B pathway, inhibiting inflammatory factors and protecting islet cells | (26) |
|  |  | *Panax quinquefolium* L. | PNPG | Inhibition of α - glucosidase | (25) |
| 22 | Ginsenoside Rc | *Panax ginseng* C.A. Mey | HUVEC cell  db/db mice | Activate IRS-1/PI3K/Akt/eNOS pathway to produce no and stimulate GLUT4 expression,  Inhibiting JNK/IKK β/NF - κ B signaling pathway and protecting islet cells | (27) |
|  |  | *Panax quinquefolium* L. | PNPG | Inhibition of α - glucosidase | (25) |
| 23 | Ginsenoside Rd | *Panax ginseng* C.A. Mey | db/db mice | Inhibition of FoxO1 activity and reduction of gluconeogenesis gene expression | (28) |
|  |  | *Panax quinquefolium* L. | PNPG | Inhibition of α - glucosidase | (25) |
| 24 | Ginsenoside Re | *Panax quinquefolium* L*.* | PNPG | Inhibition of α - glucosidase | (25) |
|  |  |  | MIN-6 cell | Protect islet beta cells | (29) |
| 25 | Ginsenoside Rg1 | *Panax ginseng* C.A. Mey | STZ induced diabetes mice | Activation of AMPK and inhibition of mTOR reduce apoptosis of islet β cells | (30) |
|  |  | *Panax quinquefolium* L*.* | PNPG | Inhibition of α - glucosidase | *(25)* |
| 26 | Ginsenoside Rg2 | *Panax ginseng* C.A. Mey | HepG2 | Activate ampk/GSK3 β/shp  Inhibition of hepatic gluconeogenesis | (31) |
|  |  | *Panax quinquefolium* L. | PNPG | Inhibition of α - glucosidase | *(25)* |
| 27 | Ginsenoside Rg3 | *Panax ginseng* C.A. Mey | Balb/c cells | Activate AMPK signaling pathway and stimulate insulin secretion | (32) |
|  |  |  | NCI-H716 cells  HD induced diabetes mice | Activate sweet receptors (T1R2/T1R3) and promote GLP-1 secretion | (33) |
|  |  |  | Balb/c mice cells | Reduce inflammatory factors and protect islet cells | (34) |
|  |  |  | STZ induced diabetes mice | Activate PPAR - γ, up regulate GLUT4 and promote glucose uptake  Activating adiponectin pathway to regulate lipid metabolism | (35) |
|  |  |  | STZ and HFD induced diabetes rats | Activate PI3K/MAPK signaling pathway to protect islet cells | (36) |
|  |  |  | L6 cells  STZ and HFD induced diabetes rats | IRS-1/Akt signaling pathway up regulates GLUT4 and promotes glucose uptake | (37) |
|  |  |  | C2C12 cells | Up regulation of PGC-1 α expression and improvement of mitochondrial function | (38) |
|  |  |  | 3T3-L1 cells | Stimulate PI3K/IRS-1 signaling pathway, up regulate GLUT4 and stimulate glucose uptake | (39) |
|  |  |  | STZ and HFD induced diabetes mice | Activate Nrf2/HO-1 signaling pathway and inhibit cell apoptosis | (40) |
| 28 | Ginsenoside Rg5 | *Panax ginseng* C.A. Mey | HFD induced diabetes mice  Primary mouse hepatocytes | Blocking HIF-1 α signaling pathway, inhibiting the expression of PEPCK and G6Pase, and inhibiting gluconeogenesis | (41) |
|  |  |  | db/db mice(leptin receptor point mutation) | Regulate intestinal flora, inhibit JNK/IRS-1 signaling pathway and reduce the production of inflammatory factors | (42) |
|  |  |  | db/db mice(leptin receptor point mutation) | Activate IRS1/PI3K/Akt/GSK3 β - GS signaling pathway and promote glycogen synthesis | (43) |
|  |  |  | db/db mice(leptin receptor point mutation) | Activate AMPK/SIRT1/PGC-1 α signaling pathway and protect mitochondria | (44) |
| 29 | Ginsenoside Rg6 | *Panax quinquefolium* L*.* | PNPG | Inhibition of α - glucosidase | (25) |
| 30 | Ginsenoside Rh1 | *Codonopsis pilosula* (Franch.) Nannf. | fluorescence based deacetylase assay | Inhibition of SIRT1 activity | (45) |
| 31 | Ginsenoside Rh2 | *Panax ginseng* C.A. Mey | pNPP  PNPG | Inhibition of PIPTB  Inhibition of α - glucosidase | (46) |
| 32 | Glycyrrhetinic Acid | *Glycyrrhiza uralensis* Fisch | insulin resistance-HepG2 cells | Activating PI3K/Akt/GSK-3 β signaling pathway and promoting glycogen synthesis | (47) |
|  |  |  | HG induced THP-1 cells | Up regulation of sRAGE and inhibition of age activity | (48) |
|  |  |  | HEK293T cells  HFD induced NAFLD mice | Inhibition of HNF4 α expression  Inhibition of G6gase and PEPCK, inhibition of gluconeogenesis | (49) |
| 33 | Glycyrrhizic acid | *Glycyrrhiza uralensis* Fisch | STZ induced diabetes mice | Inhibition of α - glucosidase  enhancing the insulin sensitivity. | (50) |
|  |  |  | HFD induced diabetes mice | Inhibit hmgb1-rage signaling pathway and age activity | (51) |
|  |  |  | 3T3-L1 cells  STZ induced diabetes mice | Activate PPAR γ and enhance glucose uptake | (52) |
| 34 | Isomogroside IVa | *Siraitia grosvenorii* (Swingle) C. Jeffrey ex Lu et Z. Y. Zhang | HepG2 cell | Improve glucose uptake | (1) |
| 35 | Isomogroside IVe | *Siraitia grosvenorii* (Swingle) C. Jeffrey ex Lu et Z. Y. Zhang | HepG2 cell | Improve glucose uptake | (1) |
| 36 | Jujuboside A | *Ziziphus jujuba* Mill. var. spinosa (Bunge) Hu ex H. F. Chou | Sprague Dawley rats | Activate AMPK/mTOR signaling pathway and protect mitochondrial function  Inhibition of Bax/caspase 9 signaling pathway and anti apoptosis | (53) |
| 37 | [Malonylginsenoside Rb1](https://www.chembk.com/en/chem/malonylginsenoside%20Rb1) | *Panax quinquefolium* L*.* | STZ induced diabetes mice | Activate IRS1/PI3K/Akt signaling pathway, activate PPAR γ, up regulate GLUT4, and promote glucose uptake | (54) |
| 38 | Maslinic acid | *Crataegus pinnatifida* Bge. | STZ induced diabetes mice | Activate AMPK/SIRT1 signaling pathway and enhance insulin sensitivity | (55) |
| 39 | Mogroside I A1 | *Siraitia grosvenorii* (Swingle) C. Jeffrey ex Lu et Z. Y. Zhang | STZ induced diabetes mice | Promote GLP-1 secretion | (56) |
| 40 | Mogroside II A1 | *Siraitia grosvenorii* (Swingle) C. Jeffrey ex Lu et Z. Y. Zhang | STZ induced diabetes mice | Promote GLP-1 secretion | (56) |
| 41 | mogroside V | *Siraitia grosvenorii* (Swingle) C. Jeffrey ex Lu et Z. Y. Zhang | Wistar rats | Promoting insulin secretion | (57) |
| 42 | Mogroside VIa | *Siraitia grosvenorii* (Swingle) C. Jeffrey ex Lu et Z. Y. Zhang | HepG2 cell | Improve glucose uptake | (1) |
| 43 | Pachymic acid | *Poria cocos (Schw.)* Wolf. | 3T3-L1 cell | Up regulation of IRS1/PI3K/Akt pathway and increase of GLUT4 expression | (58) |
|  |  |  | 3T3-L1 cell | Glucose uptake stimulating activity and insulin sensitizing activity | (12) |
|  |  |  | PNPG | Inhibition of alpha glucosidase | (4) |
| 44 | Pinicolic acid E | *Poria cocos (Schw.)* Wolf. | 3T3-L1 cell | Glucose uptake stimulating activity and insulin sensitizing activity | (12) |
| 45 | Platycodin D | *Platycodon grandiflorum*（Jacq.）A.DC. | RAW264.7 cell  [HK2](https://www.sciencedirect.com/topics/biochemistry-genetics-and-molecular-biology/hk2) cell | Activating PI3K/Akt signaling pathway and inhibiting apoptosis  Inhibit NF - κ B signaling pathway and reduce inflammation | (59) |
| 46 | polyporenic acid C | *Poria cocos (Schw.)* Wolf. | 3T3-L1 cell | Glucose uptake stimulating activity and insulin sensitizing activity | (12) |
| 47 | Poricoic acid A | *Poria cocos (Schw.)* Wolf. | PNPG | Inhibition of alpha glucosidase | (4) |
| 48 | Poricoic acid B | Poria cocos (Schw.) Wolf. | PNPG | Inhibition of alpha glucosidase | (4) |
| 49 | Poricoic acid C | *Poria cocos (Schw.)* Wolf. | PNPG | Inhibition of alpha glucosidase | (4) |
| 50 | Poricoic acid D | *Poria cocos (Schw.)* Wolf. | 3T3-L1 cell | Glucose uptake stimulating activity and insulin sensitizing activity | (12) |
| 51 | Poricoic Acid I | *Poria cocos (Schw.)* Wolf. | 3T3-L1 cell | Glucose uptake stimulating activity and insulin sensitizing activity | (12) |
| 52 | Poricoic Acid JM | *Poria cocos (Schw.)* Wolf. | 3T3-L1 cell | Glucose uptake stimulating activity and insulin sensitizing activity | (12) |
| 53 | Poricoic Acid K | *Poria cocos (Schw.)* Wolf. | 3T3-L1 cell | Glucose uptake stimulating activity and insulin sensitizing activity | (12) |
| 54 | Poricoic Acid M | *Poria cocos (Schw.)* Wolf. | 3T3-L1 cell | Glucose uptake stimulating activity and insulin sensitizing activity | (12) |
| 55 | Protodioscin | *Dioscorea opposita* Thunb. | STZ and HFD induced diabetes mice | Up regulation of GLUT4 expression and stimulation of glucose uptake  Increase adiponectin concentration and regulate lipid metabolism | (60) |
| 56 | Pseudoginsenoside F11 | *Panax quinquefolium* L*.* | 3T3-L1 cell | Activate PPAR γ, increase adiponectin secretion and regulate lipid metabolism | (61) |
| 57 | Ursolic acid | *Cornus officinalis* Sieb. et Zucc. | PNPG  HepG2 cells  STZ induced diabetes mice | Scavenging reactive oxygen species and alleviating oxidative stress  Inhibition of α - glucosidase | (62) |
|  |  |  | DNS  PNPG | Inhibition of α - amylase  Inhibition of α - glucosidase | (63) |
|  |  |  | STZ and HFD induced diabetes rats | Inhibiting JNK signaling pathway, alleviating oxidative stress and protecting islet β cells | (64) |
|  |  |  | STZ induced gestational diabetes rats | Inhibition of ages rage signaling pathway  Alleviate oxidative stress | (65) |
|  |  |  | STZ induced diabetes rats  NCI-H716 cells  CHO-K1 cells | Activate TGR5 and promote GLP-1 secretion | (66) |
|  |  |  | STZ and HFD induced diabetes mice | Inhibit the production of inflammatory factors and protect islet cells | (67) |
|  |  |  | STZ/NA induced diabetes mice | Inhibit the production of inflammatory factors | (68) |
| 58 | Versisponic acid E | *Poria cocos (Schw.)* Wolf. | 3T3-L1 cell | Glucose uptake stimulating activity and insulin sensitizing activity | (12) |

Table S4 Flavonoids from food and medicine homologyfoods and their blood glucose-regulating mechanisms

| NO | Name | Source | Experimental model | Potential mechanism | Ref. |
| --- | --- | --- | --- | --- | --- |
| 59 | (−)-Epicatechin-3-O-gallate | *Cornus officinalis* Sieb.et Zucc. | BRIN-BD11 cell  H4IIE cell | antiinflammatory | (69) |
| 60 | (3R)-5,7-Dihydroxyl-3-(2′,4′-dihydroxylbenzyl)-chroman-4-one | *Polygonatum odoratum* (Mill.) Druce | PNPG | Inhibition of alpha glucosidase | (70) |
| 61 | (3R)-5,7-Dihydroxyl-6-methyl-3-(4′-hydroxylbenzyl)-chroman-4-one | *Polygonatum odoratum* (Mill.) Druce | PNPG | Inhibition of alpha glucosidase | (70) |
| 62 | (3R)-5,7-Dihydroxyl-6-methyl-8-methoxyl-3-(4′-hydroxylbenzyl)-chroman-4-one | *Polygonatum odoratum* (Mill.) Druce | PNPG | Inhibition of alpha glucosidase | (70) |
| 63 | 26-Hydroxydaidzein | *Glycine max* (L.) Merr. | 33T3-L1 | Activation of PPAR γ promotes translocation of GLUT4 | (71) |
| 64 | 3'-Hydroxy-4',5,7-trimethoxyflavone | *Cirsium setosum* (Willd.) MB. | pNPP | Inhibit PTP1B | (72) |
| 65 | 4',5-Dihydroxy-2',3',7,8-tetramethoxylflavone | *Cirsium setosum* (Willd.) MB. | pNPP | Inhibit PTP1B | (72) |
| 66 | 4',5-Dihydroxy-7,8-dimethoxyflavone | *Cirsium setosum* (Willd.) MB. | pNPP | Inhibit PTP1B | (72) |
| 67 | 5,7-Dihydroxyl-6,8-dimethyl-3-(4’-hydroxylbenzyl)-chroman-4-one | *Polygonatum odoratum* (Mill.) Druce | 3T3-L1 cell | Activate AMPK signaling pathway | (73) |
|  |  |  | Caco-2 cell | Inhibit SGLT1 and GLUT2 | (74) |
|  |  |  | PNPG | Inhibition of alpha glucosidase | (70) |
| 68 | 5,7-Dihydroxyl-6-methyl-3-(4’-hydroxylbenzyl)-chroman-4-one | *Polygonatum odoratum* (Mill.) Druce | 3T3-L1 cell | Activate AMPK signaling pathway | (73) |
|  |  |  | Caco-2 cell | Inhibit SGLT1 and GLUT2 | (74) |
| 69 | 5,7-Dihydroxyl-6-methyl-8-methoxyl-3-(4’-hydroxylbenzyl)-chroman-4-one | *Polygonatum odoratum* (Mill.) Druce | 3T3-L1 cell  Caco-2 cell | Activate AMPK signaling pathway | (73) |
|  |  |  |  | Inhibit SGLT1 and GLUT2 | (74) |
| 70 | 6'''-Vanilloylspinosin | *Ziziphus jujuba* Mill. var. spinosa (Bunge) Hu ex H. F. Chou | DNS | Inhibition of α - amylase | (75) |
| 71 | 8-Geranyl-7, 2′, 4′-trihydroxy-isoflavone | *Pueraria lobata*（Willd.）Ohwi | PNPG | Inhibition of alpha glucosidase | (76) |
| 72 | 8-Geranyl-7, 3′, 4′-trihydroxy-isoflavone | *Pueraria lobata*（Willd.）Ohwi | PNPG | Inhibition of alpha glucosidase | (76) |
| 73 | 8-Geranyl-7, 3′-dihydroxy-4′-methoxyisoflavone | *Pueraria lobata*（Willd.）Ohwi | PNPG | Inhibition of alpha glucosidase | (76) |
| 74 | 8-Geranyl-7, 4′-dihydroxy-isOflavone | *Pueraria lobata*（Willd.）Ohwi | PNPG | Inhibition of alpha glucosidase | (76) |
| 75 | Biochanin A | *A. membranaceus* (Fisch.) Bge. | PNPG | Inhibition of α - glucosidase | (77) |
| 76 | Cajanin | *Sophora japonica* L. | HepG2 | Promote glucose absorption | (78) |
| 77 | Calycosin | *A. membranaceus* (Fisch.) Bge. | PNPG | Inhibition of α - glucosidase | (77) |
|  |  |  | GDM diabetes mice | Enhance pancreatic cell function | (79) |
| 78 | Calycosin-7-O-β-D-glucoside | *A. membranaceus* (Fisch.) Bge. | PNPG | Inhibition of α - glucosidase | (77) |
| 79 | Calycosin-7-O-β-D-glucoside-6′′-O-malonate | *A. membranaceus* (Fisch.) Bge. | PNPG | Inhibition of α - glucosidase | (77) |
| 80 | Catechin | *Nelumbo nucifera* Gaertn. | PNPG | Inhibition of α - glucosidase | (80) |
|  |  |  | HIT-T15 cell  HFD induced diabetes mice | ERK1/2 signaling pathway regulated by Ca 2+- activated PKC enhances insulin secretion in β cells | (81) |
|  |  | *Amomum tsao-ko* Crevost et Lemaire | PNPG | Inhibition of α - glucosidase | *(82)* |
| 81 | Cynaroside | *Hordeum vulgare* L. | HepG2 | Activate AMPK signaling pathway | (83) |
| 82 | Daidzein | *Pueraria lobata*（Willd.）Ohwi | PNPG | Inhibition of alpha glucosidase | (84) |
|  |  | *Glycine max* (L.) Merr. | 3T3-L1 cell | Enhance PPAR - γ activity and promote glucose uptake | (85) |
| 83 | Dehydroglyasperin D | *Glycyrrhiza uralensis* Fisch | KK-A^y^ mice | Activate ppar- γ | (86) |
| 84 | Didymin | *Citrus reticulata* Blanco | PNPG | Inhibition of α - glucosidase | (87) |
| 85 | Diosmetin | *Citrus reticulata* Blanco | HFD-fed SD rats | Inhibition of NF - κ B pathway enhances insulin sensitivity | (88) |
| 86 | Epicatechin | *Amomum tsao-ko* Crevost et Lemaire | PNPG | Inhibition of α - glucosidase | *(82)* |
|  |  |  | PNPGDiabetes mice | Inhibition of alpha glucosidase  Antioxidant stress | (89) |
| 87 | Formononetin | *A. membranaceus* (Fisch.) Bge. | PNPG | Inhibition of α - glucosidase | (77) |
| 88 | Formononetin-7-O-β-D-glucoside-6′′-O-malonate | *A. membranaceus* (Fisch.) Bge. | PNPG | Inhibition of α - glucosidase | (77) |
| 89 | Genistein | *Glycine max* (L.) Merr. | ALX induced diabetic ketoacidosis mice 1.1.3HEPG2 | Activating AMPK regulates PEPCK-C and inhibits gluconeogenesis | (90) |
|  |  |  | INS1 cell | Activation of ERK1/2 signaling pathway protects islet β cells | (91) |
|  |  |  | pNPG | Inhibition of α - glucosidase | (92) |
|  |  |  | NCI-H716 cell | Insulin stimulating effect and GLP-1 secretion in | (85) |
| 90 | Glabridin | *Glycyrrhiza uralensis* Fisch | HepG2 | Activating PI3K/Akt pathway and regulating gluconeogenesis | (93) |
|  |  |  | STZ induced diabetes mice | Protecting pancreatic cells | (94) |
| 91 | Glycitin | *Glycyrrhiza uralensis* Fisch | PNPG | Inhibition of alpha glucosidase | (95) |
| 92 | HM-chromanone | *Portulaca oleracea* L. | HepG2 cell  pNPP | Increase the phosphorylation of FoxO1 and PGC1 α and inhibit the expression of PEPCK and G6Pase  Inhibit PTP1B | (96) |
| 93 | Homoorientin | *Hordeum vulgare* L. | HepG2 | Activate AMPK signaling pathway | (83) |
| 94 | Hyperoside | *Crataegus pinnatifida* Bge. | PNPG | Inhibition of α - glucosidase | (97) |
|  |  | *Sophora japonica*L. | PNPG | Inhibition of α - glucosidase | (98) |
|  |  | *Zanthoxylum bungeanum* Maxim. | HFD and alloxan in mice | Downregulation of p65/NF - κ B and ERK/MAPK signaling pathways inhibits islet cell injury | *(99)* |
| 95 | Isoliquiritigenin | *Glycyrrhiza uralensis* Fisch | PNPG | Inhibition of alpha glucosidase | (100) |
| 96 | Isoquercitrin | *Morus alba* L. | 3T3-L1 | Suppress ages/rage  P38 MAPK/NF - κ B pathway inhibits cell injury and apoptosis | (101) |
| 97 | Isovitexin-2“-O-β-D-glucopyranoside | *Ziziphus jujuba* Mill. var. spinosa (Bunge) Hu ex H. F. Chou | DNS | Inhibition of α - amylase | (75) |
| 98 | Kaempferol | *Sophora japonica* L. | PNPG | Inhibition of α - glucosidase | (98) |
|  |  |  | HepG2 | Promote glucose absorption | (78) |
|  |  | *Morus alba* L. | pNPP | Inhibition PTP1B | (102) |
| 99 | Kaempferol-3-O-(2″-O-β-d glucopyranosyl)-β-d-glucopyranoside | *Polygonatum kingianum* Coll. et Hemsl. | 3T3-cell | Inhibit AGEs | (103) |
| 100 | Kaempferol-3-O-  α-(6′″-p-coumaroylglucosyl-β-l,2-rhamnoside) | *Polygonatum kingianum* Coll. et Hemsl. | 3T3-cell | Inhibit AGEs | (103) |
| 101 | Licochalcone-A | *Glycyrrhiza uralensis* Fisch | HFD induced diabetes mice | Activate IRS-2/PI3K/Akt pathway to protect islet β cells | (104) |
| 102 | Linarin | *Chrysanthemum indicum* L. | HepG2  MSG mice | Activate AMPK pathway | (105) |
| 103 | Liquiritigenin | *Glycyrrhiza uralensis* Fisch | Hyperglycemic Adult Zebrafish | Inhibition AGEs | (106) |
| 104 | Lobatflavate | *Pueraria lobata*（Willd.）Ohwi | PNPG | Inhibition of alpha glucosidase | (84) |
| 105 | Luteoforol | *Morus alba* L. | Caco-2 cell  Hepg2 cell | Inhibition of sucrase activity and maltase activity  Inhibit GLUT2 and SGLT1  Affect insulin resistance gene expression | (107) |
| 106 | Luteolin | *Perilla frutescens* (L.) Britt. | STZ and HFD induced diabetes mice | Activate IRS-1/PI3K/Akt pathway and up regulate GLUT-4 | (108) |
| 107 | Methylophiopogonanone A | *Polygonatum odoratum* (Mill.) Druce | Caco-2 cell | Inhibit SGLT1 and GLUT2 | (74) |
| 108 | Methylophiopogonanone B | *Polygonatum odoratum* (Mill.) Druce | Caco-2 cell | Inhibit SGLT1 and GLUT2 | (74) |
| 109 | Narcissoside | *Polygonatum kingianum* Coll. et Hemsl. | 3T3-cell | Inhibit AGEs | (103) |
| 110 | Naringenin | *Citrus reticulata* Blanco | STZ induced diabetes rats | Up regulation of GLUT4 expression | (109) |
| 111 | Naringin | *Citrus reticulata* Blanco | STZ induced diabetes rats | Up regulation of GLUT4 expression | (109) |
|  |  |  | HFD and STZ induced diabetes rats | Inhibit GSK-3 and regulate glycogen  Activation of Nrf2 signaling pathway and antioxidation | (110) |
| 112 | Neobavaisoflavone | *Pueraria lobata*（Willd.）Ohwi | PNPG | Inhibition of alpha glucosidase | (76) |
| 113 | Nicotiflorin | *Polygonatum odoratum* (Mill.) Druce | 3T3-cell | Inhibit AGEs | (103) |
| 114 | Nobiletin | *Citrus reticulata* Blanco | NIT-1 cell  STZ induced diabetes mice | Regulate Bcl-2/Bax/caspase-3 pathway to protect islet β cells.  Regulating intestinal flora | (111) |
| 115 | Ononin | *A. membranaceus* (Fisch.) Bge. | PNPG | Inhibition of α - glucosidase | (77) |
| 116 | Orobol | *Sophora japonica* L. | HepG2 | Promote glucose absorption | (78) |
| 117 | Phaseollidin | *Pueraria lobata*（Willd.）Ohwi | PNPG | Inhibition of alpha glucosidase | (76) |
| 118 | Polygonatone D | *Polygonatum odoratum* (Mill.) Druce | 3T3-L1 cell | Activate AMPK signaling pathway | (73) |
| 119 | Pratensein | *Sophora japonica* L. | HepG2 | Promote glucose absorption | (78) |
| 120 | Puerarin | *Pueraria lobata*（Willd.）Ohwi | STZ and HFD induced diabetes mice | Activating GLP-1R/Wnt/STAT3 signaling cascade to promote β - cell regeneration | (112) |
| 121 | Puerarol | *Pueraria lobata*（Willd.）Ohwi | PNPG | Inhibition of alpha glucosidase | (76) |
| 122 | Quercetin | *Crataegus pinnatifida* Bge. | STZ induced diabetes rats | Activation of SIRT1/AMPK/NF - κ B signaling pathway, anti-inflammatory | (113) |
|  |  | *Sophora japonica*L. | PNPG | Inhibition of α - glucosidase | (98) |
| 123 | Rutin | *Morus alba* L. | STZ induced diabetes rats | Hypoglycemia | (114) |
|  |  | *Sophora japonica* L. | HepG2 | Promote glucose absorption | (78) |
|  |  |  | PNPG | Inhibition of alpha glucosidase | (98) |
| 124 | Saponarin | *Hordeum vulgare* L. | HepG2 | Activate AMPK signaling pathway | (83) |
| 125 | Sinensetin | *Citrus reticulata* Blanco | BSA-fructose | Inhibition AGEs | (87) |
| 126 | Sudachitin | *Citrus reticulata* Blanco | Hepg2 cell | Promote AMPK activity and PPAR α transcription | (115) |
|  |  |  | db/db mice | Promote mitochondrial biogenesis and function, promote upregulation of Sirt1 and PGC1 α | (116) |
| 127 | Tamarixetin | *Sophora japonica* L. | HepG2 | Promote glucose absorption | (78) |
| 128 | Tangeretin | *Citrus reticulata* Blanco | Primary mouse liver cells  Diabetes db/db mice | Inhibiting mek-erk1/2 pathway in hepatocytes and enhancing insulin sensitivity | (117) |
|  |  |  | Diabetes rats | Regulation of hexokinase, pyruvate kinase, lactate dehydrogenase, glucose-6-phosphatase  Inhibition of gluconeogenesis and promote glycolysis | (118) |
| 129 | Tuberosin | *Pueraria lobata*（Willd.）Ohwi | PNPG | Inhibition of alpha glucosidase | (76) |
| 130 | Vitexcarpan | *Pueraria lobata*（Willd.）Ohwi | PNPG | Inhibition of alpha glucosidase | (76) |
| 131 | Vitexin | *Hordeum vulgare* L. | HepG2 | Activate AMPK signaling pathway | (83) |
| 132 | Xanthoangelol | *Pueraria lobata*（Willd.）Ohwi | PNPG | Inhibition of alpha glucosidase | (76) |
| 133 | Zivulgarin | *Ziziphus jujuba* Mill. var. spinosa (Bunge) Hu ex H. F. Chou | DNS | Inhibition of α - amylase | (75) |

Table S5 Alkaloid from food and medicine homology foods and their blood glucose-regulating mechanisms

| NO | Name | Source | Experimental model | Potential mechanism | Ref. |
| --- | --- | --- | --- | --- | --- |
| 134 | 1-Deoxynojirimycin | *Morus alba* L. | STZ induced diabetes rats | Improve insulin sensitivity | (119) |
| 135 | 2-Hydroxy-1-methoxyaporphine | *Nelumbo nucifera* Gaertn. | 3T3-L1 cell | Promote glucose consumption | (120) |
| 136 | 3-Hydroxy-1,2-dimethoxy-5-methyl-5H-dibenzoindol-4-one | *Houttuynia cordata* Thunb. | pNPP | Inhibits PTP1B | (121) |
| 137 | 4-Hydroxy-1,2,3-trimethoxy-  7H-dibenzo-quinolin-7-one | *Houttuynia cordata* Thunb. | pNPP | Inhibits PTP1B | (121) |
| 138 | 7-Oxodehydroasimilobine | *Houttuynia cordata* Thunb. | pNPP | Inhibits PTP1B | (121) |
| 139 | Amygdalin | *Prunus persica* (L.) Batsch | PNPG  DNS | Inhibit alpha amylase and alpha glucosidase | (122) |
| 140 | Aporphine | *Nelumbo nucifera* Gaertn. | 3T3-L1 cell | Promote glucose consumption | (120) |
| 141 | Asimilobine | *Ziziphus jujuba* Mill. var. spinosa (Bunge) Hu ex H. F. Chou | DNS | Inhibition of α - amylase | (75) |
| 142 | Caaverine | *Ziziphus jujuba* Mill. var. spinosa (Bunge) Hu ex H. F. Chou | DNS | Inhibition of α - amylase | (75) |
| 143 | Cepharadione B | *Houttuynia cordata* Thunb. | pNPP | Inhibits PTP1B | (121) |
| 144 | Fagomine | *Polygonatum odoratum* (Mill.) Druce | PNPG | Inhibition of alpha glucosidase | (123) |
| 145 | Higenamine 4′-O-β-d-glucoside | *Nelumbo nucifera* Gaertn. | L6 cell | Activate PI3K/Akt signaling pathway and up regulate GLUT4 | (124) |
| 146 | Hydroxyl-β-sanshool | *Zanthoxylum bungeanum* Maxim. | HFD and STZ induced diabetes rats | Activate AMPK/PPAR γ pathway and promote GLUT4 expression | (125) |
|  |  |  | PNPG | Inhibits α-glucosidase | (126) |
| 147 | Hydroxyl-γ-sanshool | *Zanthoxylum bungeanum* Maxim. | HFD and STZ induced diabetes rats | Activate AMPK/PPAR γ pathway and promote GLUT4 expression | (125) |
| 148 | Hydroxy-α-sanshool | *Zanthoxylum bungeanum* Maxim. | HepG2  HFD and STZ induced diabetes mice | Activate PI3K/Akt/GSK-3 β/GS signaling pathway and increase glycogen synthesis | (127) |
|  |  |  | HFD and STZ induced diabetes rats | Activate AMPK/PPAR γ pathway and promote GLUT4 expression | (125) |
|  |  |  | PNPG | Inhibits α-glucosidase | (126) |
| 149 | Kukoamine B | *Lycium barbarum* L. | db/db mice | Inhibit NF - κ B pathway and inflammation | (128) |
| 150 | Lotusine B | *Ziziphus jujuba* Mill. var. spinosa (Bunge) Hu ex H. F. Chou | DNS | Inhibition of α - amylase | (75) |
| 151 | N-cis Feruloyltyramine | *Lycium barbarum* L. | PNPG | Inhibits α-glucosidase | (129) |
| 152 | N-cis-Feruloyloctopamine | *Polygonatum odoratum* (Mill.) Druce | PNPG | Inhibits α-glucosidase | (70) |
| 153 | Neferine | *Nelumbo nucifera* Gaertn. | STZ induced diabetes rats | Activate nrf-2 pathway | (130) |
|  |  |  | HepG2 cell | Inhibition of α - glucosidase | (131) |
|  |  |  | STZ and HFD induced diabetes rats | Increase insulin sensitivity | (132) |
|  |  |  | HUVECs  cell | Blocking ROS/Akt/NF - κ B pathway inhibits high glucose induced apoptosis of endothelial cells | (133) |
|  |  |  | STZ-induced diabetic mice | Improve the expression of LxA4 and lxb4 | (134) |
| 154 | N-Nornuciferine | *Nelumbo nucifera* Gaertn. | HepG2 cell | Inhibition of α - glucosidase | (131) |
| 155 | Nornuciferine | *Ziziphus jujuba* Mill. var. spinosa (Bunge) Hu ex H. F. Chou | DNS | Inhibition of α - amylase | (75) |
| 156 | N-trans-FeruloYloctopamine | *Polygonatum odoratum* (Mill.) Druce | PNPG | Inhibits α-glucosidase | (70) |
| 157 | N-trans-Feruloyltyramine | *Polygonatum odoratum* (Mill.) Druce | PNPG | Inhibits α-glucosidase | (70) |
| 158 | N-trans-p-Coumaroyloctopamine | *Polygonatum odoratum* (Mill.) Druce | PNPG | Inhibits α-glucosidase | (70) |
| 159 | N-trans-p-Coumaroyltyramine | *Polygonatum odoratum* (Mill.) Druce | PNPG | Inhibits α-glucosidase | (70) |
| 160 | Nuciferine | *Nelumbo nucifera* Gaertn. | 3T3-L1 cell | Upregulates GLUT4 expression | (135) |
| 161 | Portulaca Neo-Oleracein A | *Portulaca oleracea* L. | STC-1 cell | Promote GLP-1 secretion | (136) |
| 162 | Portulaca Neo-Oleracein B | *Portulaca oleracea* L. | STC-1 cell | Promote GLP-1 secretion | (136) |
| 163 | Portulaca Neo-Oleracein C | *Portulaca oleracea* L. | STC-1 cell | Promote GLP-1 secretion | (136) |
| 164 | Portulaca Neo-Oleracein D | *Portulaca oleracea* L. | STC-1 cell | Promote GLP-1 secretion | (136) |
| 165 | Portulaca Neo-Oleracein E | *Portulaca oleracea* L. | STC-1 cell | Promote GLP-1 secretion | (136) |
| 166 | Portulaca Neo-Oleracein F | *Portulaca oleracea* L. | STC-1 cell | Promote GLP-1 secretion | (136) |
| 167 | Pronuciferine | *Nelumbo nucifera* Gaertn. | 3T3-L1 cell | Promote glucose consumption | (120) |
|  |  |  | 3T3-L1 cell | Upregulates GLUT4 expression | (135) |
| 168 | Roemerine | *Nelumbo nucifera* Gaertn. | 3T3-L1 cell | Promote glucose consumption | (120) |

Table S6 Phenylpropanoids from food and medicine homologyfoods and their blood glucose-regulating mechanisms

| NO | Name | Source | Experimental model | Potential mechanism | Ref. |
| --- | --- | --- | --- | --- | --- |
| 169 | 2-Oxopropyl 3-hydroxy-4-methxybenzoate | *Codonopsis pilosula* (Franch.) Nannf. | PNPG | Inhibition of α - glucosidase | (137) |
| 170 | 3-(4′-Hydroxyphenyl)-2-propenoic acid (4′′-carboxyl)-phenyl ester | *Houttuynia cordata* Thunb. | PNPG  pNPP | Inhibition of α - glucosidase  Inhibit PTP1B | (138) |
| 171 | 4(+)-Pinoresinol | *Sesamum indicum* L. | PNPG | Inhibition of α - glucosidase | (139) |
| 172 | 4-Hydroxycinnamic acid | *Cannabis sativa* L. | Wistar rat | Inhibit alpha amylase and alpha glucosidase | (140) |
| 173 | 6-Gingerol | *Zingiber officinale* (Willd.) Rosc. | STZ and HFD induced diabetic mice | Activate glp-1/camp/pka pathway,  Up regulation of GLUT4 | (141) |
| 174 | Amovillosumin A | *Amomum villosum* Lour. | db/db mice | Promote insulin secretion  Stimulate GLP-1 secretion | (142) |
| 175 | Amovillosumin B | *Amomum villosum* Lour. | db/db mice | Promote insulin secretion  Stimulate GLP-1 secretion | (142) |
| 176 | Caftaric Acid Monomethyl Ester | *Cornus officinalis* Sieb.et Zucc. | BRIN-BD11 cell  H4IIE cell | Inhibition of PEPCK expression | (69) |
| 177 | Cinnamaldehyde | *Cinnamomum cassia* (L.) D. Don | STZ induced diabetes mice | Reduce inflammatory factors | (143) |
| 178 | Cinnamic acid | *Cinnamomum cassia* (L.) D. Don | STZ induced diabetes rats | Promote GLP-1 secretion | (144) |
| 179 | Cryptochlorogenic acid | *Morus alba* L. | STZ and HFD induced diabetic mice | Activate Nrf2, antioxidant | (145) |
| 180 | Eugenol | *Eugenia caryophyllata* Thunb. | HFD induced diabetes mice | Regulating SHP/pfoxo1/pCREB/PEPCK/G6Pase signal transduction pathway to regulate liver glucose and lipid metabolism and inhibit insulin resistance | (146) |
|  |  |  | STZ induced diabetes rats | Regulate hexokinase, pyruvate kinase  Glucose 6-phosphate dehydrogenase | *(147)* |
| 181 | Licarin B | *Myristica fragrans* Houtt. | L6 cell  ICR mice | Activate AMPK/Akt signaling pathway and up regulate GLUT4 | (148) |
| 182 | Macelignan | *Myristica fragrans* Houtt. | db/db mice | Improves insulin sensitivity | (149) |
| 183 | *meso*-Dihydroguaiaretic acid | *Myristica fragrans* Houtt. | pNPP | Inhibits PTP1B | (150) |
| 184 | Methyl caffeate | *Prunus persica* (L.) Batsch | INS-1 cell  Ez-Cytox cell | Activation of IRS-2/PI3K/Akt signaling pathway  Activate PPAR - γ and promote insulin secretion | (151) |
| 185 | Myristicin | *Myristica fragrans* Houtt. | L6 cell  ICR mice | Activate AMPK/Akt signaling pathway and up regulate GLUT4 | (148) |
|  |  |  | db/db mice | Activate AMPK signaling pathway  Activate PPAR α  Activate PPAR γ | (149) |
| 186 | Nectandrin A | *Myristica fragrans* Houtt. | C2C12 cell | Activate AMPK | (152) |
| 187 | Nectandrin B | *Myristica fragrans* Houtt. | ECV 304 cell  HUVEC cell | Activate AMPK/akt signaling pathway | (153) |
| 188 | Otobaphenol | *Myristica fragrans* Houtt. | pNPP | Inhibits PTP1B | (150) |
| 189 | p-coumaric acid | *Morus alba* L. | Caco-2 cell  Hepg2 cell | Inhibition of sucrase activity and maltase activity  Inhibit GLUT2 and SGLT1  Affect insulin resistance gene expression | (107) |
| 190 | Perillaldehyde | *Perilla frutescens* (L.) Britt. | H9c2 cell | Activate Bax/Bcl-2 signaling pathway and protect islet β cells | (154) |
| 191 | Phellopterin | *Angelica dahurica* Bentham et Hooker f. | db/db mice | Activating gpr119 stimulates GLP-1 secretion | (155) |
| 192 | P-Hydroxyphenyl butanone | *Rubus chingii* Hu | Hepg2 cell | Activate IRS-1 and SHP-1, and activate ampk/akt signaling pathway | (156) |
| 193 | Rosmarinic acid | *Perilla frutescens* (L.) Britt. | STZ induced diabetic rats | Antioxidation and inflammation | (157) |
|  |  |  | STZ and HFD induced diabetic rats | Decrease the expression of PEPCK in liver  Increase GLUT4 expression in muscle | (158) |
| 194 | Safrole | *Myristica fragrans* Houtt. | Wistar Rats | Activate PPAR α,PPAR γ | (159) |
| 195 | Sesamin | *Sesamum indicum* L. | STZ induced diabetic rats | Inhibition of fatty acid synthesis | (160) |
| 196 | Sesamol | *Sesamum indicum* L. | HFD induced diabetic mice  HepG2-IR cell | Activating AMPK/Akt signaling pathway  Inhibition of PEPCK expression | (161) |
| 197 | Tetrahydrofuroguaiacin B | *Myristica fragrans* Houtt. | C2C12 cell | Activate AMPK | (152) |

Table S7 Iridoids from food and medicine homologyfoods and their blood glucose-regulating mechanisms

| NO | Name | Source | Experimental model | Potential mechanism | Ref. |
| --- | --- | --- | --- | --- | --- |
| 198 | (7R)-n-Butyl Morroniside | *Cornus officinalis* Sieb.et Zucc. | IR HepG2 | Stimulating glucose uptake by IR HepG2 cells | (162) |
| 199 | (E)-Aldosecologanin | *Lonicera japonica* Thunb. | Griess  PNPG | Inhibition of NO production  Inhibition of alpha glucosidase | (163) |
| 200 | (Z)-Aldosecologanin | *Lonicera japonica* Thunb. | Griess  PNPG | Inhibition of NO production  Inhibition of alpha glucosidase | (163) |
| 201 | 7-Epiloganin | *Cornus officinalis* Sieb.et Zucc. | IR HepG2 | Stimulating glucose uptake by IR HepG2 cells | (164) |
| 202 | Cornuofficinaliside F | *Cornus officinalis* Sieb.et Zucc. | IR HepG2 | Stimulating glucose uptake by IR HepG2 cells | (165) |
| 203 | Cornuofficinaliside H | *Cornus officinalis* Sieb.et Zucc. | IR HepG2 | Stimulating glucose uptake by IR HepG2 cells | (165) |
| 204 | Cornuofficinaliside L | *Cornus officinalis* Sieb.et Zucc. | IR HepG2 | Stimulating glucose uptake by IR HepG2 cells | (165) |
| 205 | Cornuofficinaliside O | *Cornus officinalis* Sieb.et Zucc. | IR HepG2 | Stimulating glucose uptake by IR HepG2 cells | (165) |
| 206 | Cornusdiridoid A | *Cornus officinalis* Sieb.et Zucc. | IR HepG2 | Stimulating glucose uptake by IR HepG2 cells | (162) |
| 207 | Cornuside K | *Cornus officinalis* Sieb.et Zucc. | IR HepG2 | Stimulating glucose uptake by IR HepG2 cells | (162) |
| 208 | Gardenoside | *Gardenia jasminoides* ellis | PNPG | Inhibition of α - glucosidase | (166) |
| 209 | Genipin | *Gardenia jasminoides* ellis | STZ induced diabetic rat | Down regulation of TNF - α and IL-6 gene expression, insulin resistance in DIO rats | (167) |
|  |  |  | C2C12 myoblasts | Activate IRS-1, PI3-K and up regulate GLUT4 | (168) |
|  |  |  | STZ induced diabetic mice | Regulating JNK/Akt signaling pathway and inhibiting liver oxidative stress and mitochondrial dysfunction | (169) |
| 210 | Geniposide | *Gardenia jasminoides* ellis | STZ and HFD induced diabetic mice | Activate RBP4 | (170) |
|  |  |  | HepG2 cell | Regulation of ampk-foxo1 signaling pathway inhibits hepatic gluconeogenesis | (171) |
|  |  |  | STZ and HFD induced diabetic mice | Inhibition of alpha glucosidase | (172) |
| 211 | Loganin | *Cornus officinalis* Sieb.et Zucc. | PNPG  HepG2 cell  STZ induced diabetic mice | Inhibition of alpha glucosidase  anti-oxidative stress | (173) |
| 212 | Morroniside | *Cornus officinalis* Sieb.et Zucc. | STZ induced diabetic rat | Inhibited AGEs | (174) |
| 213 | Neocornuside A | *Cornus officinalis* Sieb.et Zucc. | IR HepG2 | Stimulating glucose uptake by IR HepG2 cells | (164) |
| 214 | Neocornuside C | *Cornus officinalis* Sieb.et Zucc. | IR HepG2 | Stimulating glucose uptake by IR HepG2 cells | (164) |
| 215 | Neocornuside F | *Cornus officinalis* Sieb.et Zucc. | IR HepG2 | Stimulating glucose uptake by IR HepG2 cells | (162) |
| 216 | Neocornuside H | *Cornus officinalis* Sieb.et Zucc. | IR HepG2 | Stimulating glucose uptake by IR HepG2 cells | (162) |

Table S8 Polysaccharides from food and medicine homology foods and their glucose-regulating mechanisms

| Name | Source | Experimental model | Potential mechanism | Ref. |
| --- | --- | --- | --- | --- |
| APS | *A. membranaceus* (Fisch.) Bge. | STZ induced diabetes rats | Inhibition of alpha amylase activity | (175) |
|  |  | STZ induced diabetes rats | Inhibit ed PTP1B | (176, 177) |
|  |  | L6  cells | Activating AMPK signaling pathway to stimulate glucose uptake | (178) |
| AMP |  | db/db mice | Regulate intestinal flora and stimulate GLP-1 secretion | (179) |
|  |  | PNPG  HFD induced diabetes mice | Inhibition of alpha amylase activity.  Alleviate oxidative stress | (180) |
| APP |  | STZ and HFD induced diabetes mice | Inhibit intestinal inflammation and oxidative stress, and regulate intestinal flora | (181) |
| AERP |  | db/db mice(leptin receptor point mutation) | Regulating intestinal flora | (182) |
| ACGDP | *Allium chinense* G. Don | PNPG | Inhibition of alpha glucosidase | (183) |
| [ASP](https://www.sciencedirect.com/topics/medicine-and-dentistry/angelica-sinensis) | Angelica sinensis (Oliv.) Diels | HFD induced diabetes mice | It can restore the balance of Bcl-2/Bax and inhibit the apoptosis of pancreatic β cells.  Promote insulin secretion | (184) |
|  |  | STZ induced diabetes mice | PI3K/Akt signaling pathway is activated to induce GLUT2 translocation to the cell membrane, thus promoting the synthesis of liver glycogen.  Upregulation of PPAR γ | (185) |
| FCP40 | *Citrus medica* L. var. sarcodactylis Swingle | DNS | Inhibition of α - amylase | (186) |
| FCP-2-1 | *Citrus medica* L. var. sarcodactylis Swingle | PNPG  DNS  BSA-fructose  3T3-L1 cell | Inhibition of α - glucosidase  Inhibition of α - amylase  Inhibited AGEs  Activate pi3k/akt pathway | (187) |
| FCP-2 | *Citrus medica* L. var. sarcodactylis Swingle | PNPG  DNS  BSA-fructose  3T3-L1 cell | Inhibition of α - glucosidase  Inhibition of α - amylase  Inhibited AGEs  Activate pi3k/akt pathway | (187) |
| PCRPs-1 | *Citrus reticulata* Blanco | PNPG | Inhibition of α - glucosidase | (188) |
| CERP1 | *Codonopsis pilosula* (Franch.) Nannf. | INS-1 cell  STZ and HFD induced diabetes mice | Enhanced HK and PK, promote glycolysis | (189) |
| WCP5 | *Codonopsis pilosula* (Franch.) Nannf. | PNPG  DNS | Inhibition of α - glucosidase  Inhibition of α - amylase | (190) |
| CSP | *Coix lacryma-jobi* L.var. ma-yuen (Roman.) Stapf | STZ and HFD  induced diabetes mice | Regulating intestinal flora to increase the level of SCFA, thereby activating IGF1/PI3K/Akt signaling pathway and regulating glucose metabolism | (191) |
| PFC-3 | *Cornus officinalis* Sieb.et Zucc. | In vitro experiments  HepG2 cell  STZ induced diabetes rats | Inhibition of alpha amylase activity；  Protected the liver | (192) |
| Hawthorn polysaccharide | *Crataegus pinnatifida* Bge. | PNPG | Inhibition of alpha glucosidase activity | (193) |
| CYP | *Dioscorea opposita* Thunb. | HFD induced diabetes mice | Inhibited NF - κ B/p65 signaling pathway, protecting islet cells and improving insulin resistance | (194) |
| DTP | *Dioscorea opposita* Thunb. | STZ induced diabetes rats | Enhance SOD and CAT activity, antioxidant | (195) |
| DBP1 | *Dioscorea opposita* Thunb. | PNPG  DNS | Inhibition of α - glucosidase  Inhibition of α - amylase | (196) |
| DBP2 | *Dioscorea opposita* Thunb. | PNPG  DNS | Inhibition of α - glucosidase  Inhibition of α - amylase | (196) |
| WHBP | *Dolichos lablab* L. | Wistar rats | Inhibit the expression of SGLT1 to reduce the absorption of glucose into the blood and improve insulin resistance | (197) |
|  |  |  | Regulating intestinal flora composition and its metabolite SCFA | (198) |
|  |  |  | Increase GLP-1 secretion | (199) |
| EFSP-1 | *Euryale ferox* salisb. | 3T3-L1 cell  HepG2 cell | Activate PI3K/Akt signaling pathway and up regulate GLUT-4 | (200) |
| PECG | *Gallus gallus domesticus* Brisson | STZ and HFD  induced diabetes mice | Regulating serum levels of TC and TG | (201) |
| PSG-1 | *Ganoderma lucidum* (Leyss.ex Fr.) Karst. | HFD induced diabetes rats | Inhibition of Bax expression and increase of Bcl-2 protein  Protect beta cells | (202) |
| F31 | *Ganoderma lucidum* (Leyss.ex Fr.) Karst. | db/db mice | Activate AMPK signaling pathway and up regulate GLUT4 expression | (203) |
| GLP | *Ganoderma lucidum* (Leyss.ex Fr.) Karst. | STZ and HFD  induced diabetes mice | Regulating intestinal flora | (204) |
|  |  | HFD  induced diabetes mice | Inhibit inflammation and improve systemic insulin sensitivity | (205) |
| GaE-R | *Gastrodia elata* Bl. | PNPG | Inhibition of α - glucosidase | (206) |
| Glycyrrhiza polysaccharide | *Glycyrrhiza uralensis* Fisch | STZ induced diabetes mice | anti-oxidative stress | (207) |
| Lily polysaccharideIII | *Lonicera japonica* Thunb. | STZ induced diabetes mice | Antioxidant, protect islet cellsPromote HK and glycogen synthesis  Regulating the tricarboxylic acid cycle | (208) |
| Blue honeysuckle polysaccharides | *Lonicera japonica* Thunb. | PNPG  DNS | Inhibition of α - glucosidase  Inhibition of α - amylase | (209) |
| LMPB | *Lonicera japonica* Thunb. | PNPG  DNS  STZ induced diabetes rats | Inhibition of α - glucosidase  Inhibition of α - amylase  The levels of pyruvate kinase and hexokinase were significantly increased, and promote glycolysis | (210) |
| LBP | *Lycium barbarum* L. | HFD induced diabetes mice | Regulating intestinal flora | (211) |
|  |  | STZ and HFD  induced diabetes rats | Enhance GLP-1 secretion  Activate irs/pi3k/akt signaling pathway, promote GLUT2, promote glucose uptake, and inhibit gluconeogenesis  Regulating intestinal flora | (212) |
|  |  | STZ and HFD induced diabetes mice | Restore the composition of intestinal microbiota  Enhancing intestinal barrier function in mice | (213) |
|  |  | STZ and HFD induced diabetes mice | Inhibition of NF - κ B signaling pathway inhibits inflammation | (214) |
|  |  | Caco2 cell | Down regulation of SGLT-1 expression | (215) |
| MHPs | *Mentha haplocalyx* Briq. | PNPG  DNS | Inhibition of α - glucosidase  Inhibition of α - amylase | (216) |
| MLPII | *Morus alba* L. | STZ and HFD  induced diabetes rats | Activate Bcl-2/Bax signaling pathway and protect islet cells  Restore the expression level of PDX-1, improve insulin secretion ability and promote the expression of GLUT2 | (217) |
| MFP4P | *Morus alba* L. | L02 cell  RIN-m5f cell  db/db mice | Promote β - cell proliferation and insulin secretion, and relieve insulin resistance in hepatocytes | (218) |
| MFP50 | *Morus alba* L. | STZ and HFD  induced diabetes rats | Activation of PI3K/Akt pathway | (219) |
| MFP | *Morus alba* L. | RIN-m5f cell  STZ induced diabetes mice | Protect islet beta cells | (220) |
|  |  | STZ and HFD induced diabetes mice | Regulation of intestinal flora  Added short chain fatty acids (SCFAs) | (221) |
| FMP | *Morus alba* L. | STZ and HFD induced diabetes mice | Inhibiting the activation of TLR4/MyD88/NF - κ B pathway to inhibit intestinal inflammation and oxidative stress | (222) |
| MFP-III | *Morus alba* L. | PNPG  DNS | Inhibition of α - glucosidase  Inhibition of α - amylase | (223) |
| LLPs | *Nelumbo nucifera* Gaertn. | PNPG | Inhibition of α - glucosidase | (224) |
| LLP-D | *Nelumbo nucifera* Gaertn. | PNPG  DNS | Inhibition of α - glucosidase  Inhibition of α - amylase | (225) |
| LLP-M | *Nelumbo nucifera* Gaertn. | PNPG | Inhibition of α - glucosidase | (226) |
| LSRS | *Nelumbo nucifera* Gaertn. | STZ and HFD induced diabetes mice | Regulating insulin secretion | (227) |
| American ginseng polysaccharide | *Panacis quinquefolii* radix | Alloxan induced diabetes mice | anti-oxidative stress  To reduce the damage of islet β cells | (228) |
| GPS | *Panax ginseng* C.A. Mey | Wistar rats | Activate PGC-1 α pathway and improve antioxidant defense system  Promote glycolysis | (229) |
| GRO-N | *Panax ginseng* C.A. Mey | PNPG  DNS | Inhibition of α - glucosidase  Inhibition of α - amylase | (230) |
| WGPA | *Panax ginseng* C.A. Mey | STZ induced diabetes mice | Inhibition of gluconeogenesis | (231) |
| PLP-2-1 | *Perilla frutescens*(L.) Britt. | PNPG  DNS | Inhibition of α - glucosidase  Inhibition of α - amylase | (232) |
| PPLPs | *Perilla frutescens*(L.) Britt. | STZ induced diabetes mice | Antioxidant stress  Activate pi3k/akt/glut4 signaling pathway | (233) |
| PGPs | *Platycodon grandiflorum*（Jacq.）A.DC. | PNPG  HepG2 cell  DNS | Inhibition of alpha glucosidase；  Promote glycogen synthesis  Inhibition of α - amylase | (234) |
| PSPK | *Polygonatum kingianum* Coll. et Hemsl. | STZ and HFD  induced diabetes rats | Regulating gut microbiota；  Increase SCFA concentration | (235) |
| PP | *Polygonatum kingianum* Coll. et Hemsl. | db/db mice | Promote glycogen synthesis；  Improve gut microbiota | (236) |
| PSPW | *Polygonatum kingianum* Coll. et Hemsl. | STZ and HFD  induced diabetes mice | Activation of PI3K/Akt signaling pathway regulates glucose metabolism | (237) |
| PKPs-1 | *Polygonatum kingianum* Coll. et Hemsl. | HepG2 cell | Up regulation of IRS1/PI3K/Akt pathway alleviates insulin resistance | (238) |
| YZ-2 | *Polygonatum odoratum* (Mill.) Druce | STZ and HFD induced diabetes mice | Reduce oxidative stress  Inhibition of α - glucosidase activity  Improve insulin resistance | (239) |
| POP | *Polygonatum odoratum* (Mill.) Druce | STZ  induced diabetes rats | Up regulation of Nrf2 mediated HO-1 involved in PI3K/Akt pathway regulating lipid metabolism | (240) |
|  |  | HFD  induced diabetes mice | Increase SCFA concentration | (241) |
| EA-PAOA | *Polygonatum odoratum* (Mill.) Druce | HFD  induced diabetes mice | Protect islet beta cells | (242) |
| Poria cocos polysaccharide | *Poria cocos* (Schw.) Wolf. | STZ and HFD induced diabetes rats | Up regulation of PI3K/Akt/FoxO1 pathway, down-regulation of PEPCK and G6Pase expression, and inhibition of hepatic gluconeogenesis | (243) |
| PCP | *Poria cocos* (Schw.) Wolf. | STZ and HFD induced diabetes mice | Activating GLUT2/PI3K/Akt signaling pathway | (244) |
| POP | *Portulaca oleracea* L. | STZ and HFD induced diabetes mice | Activate PPAR γ  Down regulation of PKC gene expression | (245) |
| PLP1 | *Pueraria lobata*（Willd.）Ohwi | DNS  PNPG | Inhibition of α - glucosidase  Inhibition of α - amylase | (246) |
| PLP | *Pueraria lobata*（Willd.）Ohwi | db/db mice | Activate pi3k/akt signaling pathway | (247) |
| FPZ | *Rubus chingii* Hu | STZ induced diabetes mice | Activating AMPK/akt signaling pathway | (248) |
| BBP-24-3 | *Rubus chingii* Hu | PNPG | Inhibition of α - glucosidase | (249) |
| RCPI | *Rubus chingii* Hu | DNS | Inhibition of α - amylase | (250) |
| Lobster Sauce  polysaccharide | *Sojae semen* praeparatum | PNPG | Inhibition of alpha glucosidase | (251) |
| TMP | *Taraxacum mongolicum* Hand. -Mazz. | sheep white adipocyte precursor cells | Regulating Akt/GSK3 β signaling pathway | (252) |
| PD | *Taraxacum mongolicum* Hand. -Mazz. | PNPG | Inhibition of α - glucosidase | (253) |
| Se-DRP | *Taraxacum mongolicum* Hand. -Mazz. | PNPG | Inhibition rate of α-amylase activity | (254) |
| GSLP | *Zingiber officinale* (Willd.) Rosc. | PNPG | Inhibition of alpha glucosidase activity | (255) |
| ZSP | *Ziziphus jujuba* Mill. | HFD induced diabetes mice | Reduce insulin resistance | (256) |

Table S9 Other compounds from food and medicine homology foods and their glucose-regulating mechanisms

| NO | Name | Type | Source | Experimental model | Potential mechanism | Ref. |
| --- | --- | --- | --- | --- | --- | --- |
| 217 | Almond peptide | Active peptides | *Semen armeniacae* Amarum. | PNPG | Inhibition of alpha glucosidase | (257) |
| 218 | FR | Active peptides | *Cannabis sativa* L | PNPG  ALX induced diabetic rats | Inhibition of α - glucosidase  Inhibit insulin resistance | (258) |
| 219 | FY | Active peptides | *Cannabis sativa* L | PNPG  ALX induced diabetic rats | Inhibition of α - glucosidase  Inhibit insulin resistance | (258) |
| 220 | Ginseng Polypeptide | Active peptides | *Panax ginseng* C.A. Mey | db/db mice | Activation of PI3K/Akt/GSK3 β signaling pathway  Inhibition of ERK 1/2 signaling pathway | (259) |
| 221 | IAF | Active peptides | *Cannabis sativa* L | PNPG | Inhibition of α - glucosidase | (258) |
| 222 | INPLL | Active peptides | *Cannabis sativa* L | PNPG | Inhibition of α - glucosidase | (258) |
| 223 | SPVI | Active peptides | *Cannabis sativa* L | PNPG  ALX induced diabetic rats | Inhibition of α - glucosidase  Inhibit insulin resistance | (258) |
| 224 | TGLGR | Active peptides | *Cannabis sativa* L | PNPG  ALX induced diabetic rats | Inhibition of α - glucosidase  Inhibit insulin resistance | (258) |
| 225 | Malic acid | Carboxylic acid | *Prunus mume* (Sieh.) Sieb. et Zucc. | ICR mice | Participate in the tricarboxylic acid cycle | (260) |
| 226 | Citric acid | Carboxylic acid | *Prunus mume* (Sieh.) Sieb. et Zucc. | ICR mice | Participate in the tricarboxylic acid cycle | (260) |
| 227 | Crocetin | Carotenoid | *Cornus officinalis* Sieb.et Zucc. | STZ induced diabetic rat | Inhibit the production of inflammatory factors | (261) |
| 228 | Curcumin | Diketone | *Curcuma longa* L. rhizomes | 2 diabetic KK-A(y) mice | Activate ppar- γ | (262) |
|  |  |  |  | PNPG | Inhibition of α - glucosidase | (263, 264) |
|  |  |  |  | STZ induced diabetic rat | Inhibiting NF - κ B signaling pathway and protecting islet cells | (265) |
| 229 | Palatinose | Disaccharide | *Hordeum vulgare* L. | Dog | Promote GLP-1 secretion | (266) |
| 230 | Crocin I | Diterpenoid | *Gardenia jasminoides* ellis | STZ and HFD induced diabetic mice | Inhibition of alpha glucosidase | (172) |
| 231 | Allantoin | Hydantoins | *Dioscorea opposita* Thunb. | STZ induced diabetic mice | anti-oxidative stress  Promote GLP-1 secretion | (267) |
| 232 | Gallic Acid | Phenol | *Cornus officinalis* Sieb.et Zucc. | BRIN-BD11 cell  H4IIE cell | antiinflammatory | (69) |
| 233 | Catechol | Phenol | *Polygonatum kingianum* Coll. et Hemsl. | 3T3-cell | Inhibit AGEs | (103) |
| 234 | Demethoxycurcumin | Phenol | *Curcuma longa* L. rhizomes | 2 diabetic KK-A(y) mice | Activate ppar- γ | (262) |
| 235 | Bisdemethoxycurcumin | Phenol | *Curcuma longa* L. rhizomes | 2 diabetic KK-A(y) mice | Activate ppar- γ | (262) |
| 236 | [Gastrodin](https://www.chemsrc.com/en/cas/62499-27-8_829861.html) | Phenol | *Gastrodia elata* bl. | HFD induced diabetic mice | Activating PI3K/Akt pathway promotes GATA1 phosphorylation and usp4 expression | (268) |
| 237 | Vanillic acid | Phenol | *Gastrodia elata* bl. | BRIN-BD11 cell | Activate ERK1/2 signaling pathway and protect islet β cells | (269) |
| 238 | Ginnalin A | Phenol | *Houttuynia cordata* Thunb. | PNPG  pNPP | Inhibition of α - glucosidase  Inhibit PTP1B | (138) |
| 239 | Neochlorogenic acid | Phenol | *Houttuynia cordata* Thunb. | PNPG  Zebrafish | Inhibition of α - glucosidase | (270) |
| 240 | Ellagic acid | Phenol | *Rubus chingii* Hu | HFD induced diabetic rats | Protect pancreatic β cells and stimulate insulin secretion | (271) |
| 241 | Emodin | Quinones | *Cannabis sativa* L. | PNPG  pNPP | Inhibition of PTP1B and α - glucosidase | (272) |
| 242 | Physcion | Quinones | *Cannabis sativa* L. | PNPG | Inhibition of α - glucosidase | (273) |
| 243 | Chrysootusin | Quinones | *Cannabis sativa* L. | PNPG | Inhibition of α - glucosidase |  |
| 244 | Aurantio-obtusin | Quinones | *Cannabis sativa* L. | PNPG | Inhibition of α - glucosidase |  |
| 245 | Obtusifolin | Quinones | *Cannabis sativa* L. | PNPG | Inhibition of α - glucosidase |  |
|  |  |  |  | STZ induced diabetic mice | anti-oxidative stress | (274) |
| 246 | Alaternin | Quinones | *Cassia obtusifolia* L. | pNPP  PNPG | Inhibition of PTP1B and α - glucosidase | (272) |
| 247 | Hypericin | Quinones | *Sophora japonica*L. | PNPG | Inhibition of α - glucosidase | (98) |
| 248 | Ar-turmerone | Sesquiterpenes | *Curcuma longa* L. rhizomes | 2 diabetic KK-A(y) mice | Activate ppar- γ | (262) |
|  |  |  |  | PNPG  DNS | Inhibition of α - glucosidase  Inhibition of α - amylase | (275) |
| 249 | Patchouli alcohol | Sesquiterpenes | *Pogostemon cablin* (Blanco) Benth. | STZ and HFD induced diabetic mice | Activate AMPK/Akt signaling pathway and enhance glucose uptake | (276) |
| 250 | 10α-Hydroperoxy-guaia-1,11-diene | Sesquiterpenes | *Pogostemon cablin* (Blanco) Benth. | C2C12 cell | Up regulation of GLUT4 expression | (277) |

Table S10 Potential side effects of food and medicine homology foods

| Name | Side effects | Specific manifestations | Ref. |
| --- | --- | --- | --- |
| 6-Gingerol | Drug metabolism | Inhibition of CYP3A4 and CYP2D6 | (278) |
| Astragaloside IV | Pregnant/breastfeeding risks | Intravenous administration of ≥ 1 mg/kg may result in maternal toxicity; >0.5   mg/kg may cause fetal toxicity | (279) |
| Astragaloside IV | Drug metabolism | Inhibition of CYP2D6 | (280) |
| Calycosin | Drug metabolism | Inhibition of CYP1A2, CYP2D6 and CYP2C9 | (281) |
| Curcumin | Drug metabolism | Inhibition of CYP1A1,CYP1A2,CYP2A6 and CYP3A4 | (282) |
| Daidzein | **Gastrointestinal discomfort** | Diarrhea occurs at high doses (safe dose>500 mg/day) | (283) |
| Ellagic acid | Drug metabolism | Inhibition of CYP3A4 and CYP2D6 | (284) |
| **Formononetin** | **High dose short-term response** | Transient vomiting and vascular irritation (which can recover on its own) may occur above 300 mg/kg · d, but it does not result in death or severe organ damage. | (285) |
| Ginsenoside CK | Hepatotoxicity risk | Dogs in the 36 mg/kg group experienced significant weight loss and reversible liver toxicity | (286) |
| Ginsenoside Rb1 | **Hepatotoxicity risk** | Occasional mild liver enzyme fluctuations at extremely high doses (≥ 300 mg/kg) | (287) |
| Ginsenoside Rb2 | **Gastrointestinal discomfort** | Mild nausea, vomiting, diarrhea, abdominal distension and other symptoms were observed in animal experiments with high doses (≥ 50 mg/kg) | (288) |
| Ginsenoside Rg1 | Neurological symptoms | Mild central inhibition may occur at higher doses | (289) |
| Glycyrrhetinic Acid | **Gastrointestinal discomfort** | When the daily intake exceeds 400mg, nausea, vomiting, abdominal distension, abdominal pain, and diarrhea may occur | (290) |
| Kaempferol | Risk of bleeding | Inhibiting platelet activity and combining anticoagulants or antiplatelet drugs may increase the risk of bleeding. | (291) |
| Linarin | Neurological symptoms | High concentrations (>20 µ M) in vitro may affect cellular metabolism | (105) |
| Naringin | Hepatotoxicity risk | There is a trend of abnormal liver function and impaired kidney function in high-dose (≥ 200 mg · kg ⁻¹) animal experiments | (292) |
| Neferine | **Neurological symptoms** | Calcium homeostasis disorder leading to decreased myocardial contractility in in vitro myocardial cell experiments | (293) |
| Nobiletin | Risk of bleeding | It can inhibit platelet activity and may increase the risk of bleeding with antiplatelet or anticoagulant drugs. | (294) |
| Oleanolic acid | Cardiovascular disease risk | Enhanced thrombin and promoted arterial plaque formation | (295) |
| **Puerarin** | **Gastrointestinal discomfort** | Temporary bloating, stomach discomfort, heartburn | (296) |
| Roemerine | Inducing oxidative stress | Accumulation within bacterial cells triggers oxidative stress and generates a large amount of reactive oxygen species (ROS), leading to disruption of the iron uptake pathway. | (297) |
| Sinensetin | Pregnant/breastfeeding risks | In zebrafish embryo experiments, only about 40% of embryos survived at 100 µ M (survival rate of 94.6%), indicating a risk of embryo death at higher concentrations. | (298) |
| Ursolic acid | Cardiovascular disease risk | Enhanced thrombin and promoted arterial plaque formation | (295) |

Table S11. food and medicine homology foods hypoglycemic products

| Classes | Name | Materials | Ref. |
| --- | --- | --- | --- |
| Snack Foods | Astragalus membranaceus and bagasse dietary fiber biscuit | *Astragalus membranaceus* | (299) |
|  | Bitter gourd mulberry leaf biscuit | *Morus alba L* | (300) |
|  | Black Gastrodia elata meal substitute powder | *Gastrodia elata Bl* | (301) |
|  | Coarse grain biscuit | *Sesamum indicum* | (302) |
|  | Compound hypoglycemic meal substitute powder | *Puerariae Lobatae Radix, Siraitia grosvenorii, Chrysanthemum morifolium* | (303) |
|  | Food and medicine homologous compound meal substitute powder | *Puerariae Lobatae Radix, Poria cocos, Polygonatum sibiricum, Lycium chinense Miller* | (304) |
|  | Hypoglycemic chocolate | *Crataegus pinnatifida, Prunus mume* | (305) |
|  | Low GI wolfberry cereal meal substitute | *Lycium chinense Miller* | (306) |
|  | Low glycemic index milk based meal replacer | *Dioscoreae Rhizoma,Puerariae Lobatae Radix, Coicis Semen, Morus alba L* | (307) |
|  | Sugar control cake | *Semen Armeniacae Amarum, Siraitia grosvenorii* | (308) |
| Functional Beverages | Astragalus health wine | *Astragalus membranaceus* | (309) |
|  | Coix seed milk | *Coicis Semen* | (310) |
|  | Dandelion health drink | *Taraxacum mongolicum* | (311) |
|  | Ganoderma lucidum polysaccharide beverage | *Ganoderma lucidum* | (312) |
|  | Hawthorn lotus leaf buckwheat plant beverage | *Crataegus pinnatifida,Nelumbinis Folium* | (313) |
|  | Lycium barbarum vinegar beverage fermented by Kombucha | *Lycium chinense Miller* | (314) |
|  | Mulberry fermented wine | *Morus alba L* | (315) |


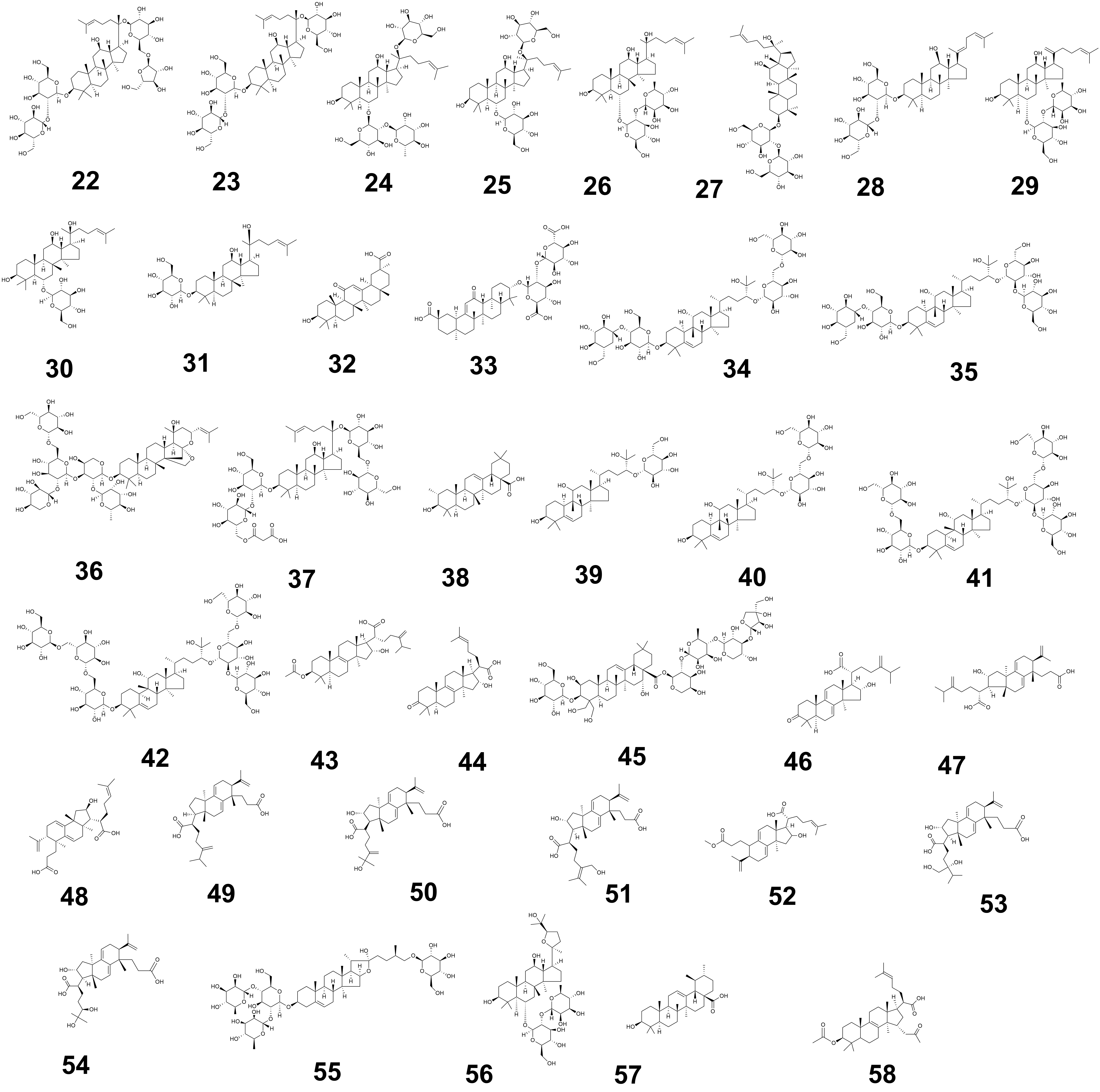


Figure.S1.The structure of triterpenoids(22-58)with blood glucose-regulating effects


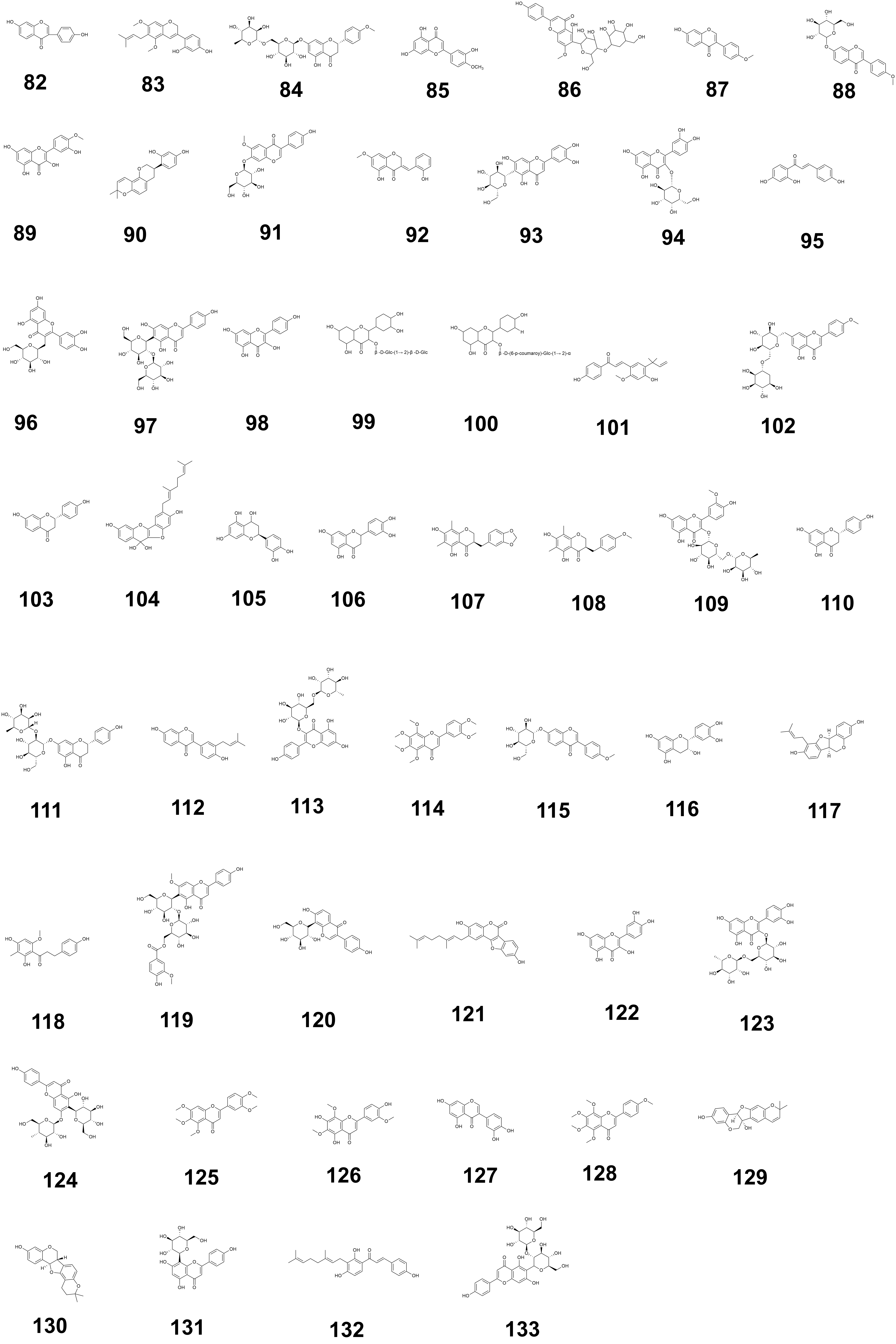


Figure.S2. The structure of flavonoids(59-133) with blood glucose-regulating effects

1. Li F, Yang F, Liu X, Wang L, Chen B, Li L, et al. Cucurbitane glycosides from the fruit of Siraitia grosvenori and their effects on glucose uptake in human HepG2 cells in vitro. Food Chemistry. 2017;228:567-73.（doi:<https://doi.org/10.1016/j.foodchem.2017.02.018>）

2. Bing L, Yue WR, Ye Z, Fan YY, Xin ZZ, Di HF, et al. Triterpenoids with α-glucosidase inhibitory activities from the roots of Codonopsis pilosula var. modesta. Journal of Chemical Research. 2021;45(5-6):635-8

3. Ahmed D, Khan MI, Sharma M, Khan MF. Novel pentacyclic triterpene isolated from seeds of Euryale Ferox Salisb. ameliorates diabetes in streptozotocin induced diabetic rats. Interdiscip Toxicol. 2018;11(4):275-88.（doi:10.2478/intox-2018-0027）

4. Ma C, Lu J, Ren M, Wang Q, Li C, Xi X, et al. Rapid identification of α-glucosidase inhibitors from Poria using spectrum-effect, component knock-out, and molecular docking technique. Front Nutr. 2023;10:1089829.（doi:10.3389/fnut.2023.1089829）

5. Chen X-b, Zhuang J-j, Liu J-h, Lei M, Ma L, Chen J, et al. Potential AMPK activators of cucurbitane triterpenoids from Siraitia grosvenorii Swingle. Bioorganic & Medicinal Chemistry. 2011;19(19):5776-81.（doi:<https://doi.org/10.1016/j.bmc.2011.08.030>）

6. Gong P, Xiao X, Wang S, Shi F, Liu N, Chen X, et al. Hypoglycemic effect of astragaloside IV via modulating gut microbiota and regulating AMPK/SIRT1 and PI3K/AKT pathway. J Ethnopharmacol. 2021;281:114558.（doi:10.1016/j.jep.2021.114558）

7. Lv L, Wu SY, Wang GF, Zhang JJ, Pang JX, Liu ZQ, et al. Effect of astragaloside IV on hepatic glucose-regulating enzymes in diabetic mice induced by a high-fat diet and streptozotocin. Phytother Res. 2010;24(2):219-24.（doi:10.1002/ptr.2915）

8. Deng LL. Astragaloside IV as Potential Antioxidant Against Diabetic Ketoacidosis in Juvenile Mice Through Activating JNK/Nrf2 Signaling Pathway. Arch Med Res. 2020;51(7):654-63.（doi:10.1016/j.arcmed.2020.06.013）

9. Zhang R, Xing B, Zhao J, Zhang X, Zhou L, Yang S, et al. Astragaloside IV relieves gestational diabetes mellitus in genetic mice through reducing hepatic gluconeogenesis. Can J Physiol Pharmacol. 2020;98(7):466-72.（doi:10.1139/cjpp-2019-0548）

10. Zhu R, Zheng J, Chen L, Gu B, Huang S. Astragaloside IV facilitates glucose transport in C2C12 myotubes through the IRS1/AKT pathway and suppresses the palmitate-induced activation of the IKK/IκBα pathway. Int J Mol Med. 2016;37(6):1697-705.（doi:10.3892/ijmm.2016.2555）

11. Jun HAN, Qinfang Z, Liangzi F, Xiaolong H. Screening and functional evaluation of the glucose-lowering active compounds of total saponins of Baibiandou (Lablab Semen Album). Digital Chinese Medicine. 2021;4(3):229-40.（doi:<https://doi.org/10.1016/j.dcmed.2021.09.007>）

12. Chen B, Zhang J, Han J, Zhao R, Bao L, Huang Y, et al. Lanostane Triterpenoids with Glucose-Uptake-Stimulatory Activity from Peels of the Cultivated Edible Mushroom Wolfiporia cocos. Journal of Agricultural and Food Chemistry. 2019;67(26):7348-64.（doi:10.1021/acs.jafc.9b02606）

13. Sato M, Tai T, Nunoura Y, Yajima Y, Kawashima S, Tanaka K. Dehydrotrametenolic Acid Induces Preadipocyte Differentiation and Sensitizes Animal Models of Noninsulin-Dependent Diabetes Mellitus to Insulin. Biological and Pharmaceutical Bulletin. 2002;25(1):81-6.（doi:10.1248/bpb.25.81）

14. Sato M, Tai T, Nunoura Y, Yajima Y, Kawashima S, Tanaka K. Dehydrotrametenolic acid induces preadipocyte differentiation and sensitizes animal models of noninsulin-dependent diabetes mellitus to insulin. Biol Pharm Bull. 2002;25(1):81-6.（doi:10.1248/bpb.25.81）

15. Ren L. Protective effect of ganoderic acid against the streptozotocin induced diabetes, inflammation, hyperlipidemia and microbiota imbalance in diabetic rats. Saudi J Biol Sci. 2019;26(8):1961-72.（doi:10.1016/j.sjbs.2019.07.005）

16. Tian F, Wang X, Ni H, Feng X, Yuan X, Huang Q. The ginsenoside metabolite compound K stimulates glucagon-like peptide-1 secretion in NCI-H716 cells by regulating the RhoA/ROCKs/YAP signaling pathway and cytoskeleton formation. J Pharmacol Sci. 2021;145(1):88-96.（doi:10.1016/j.jphs.2020.11.005）

17. Wei S, Li W, Yu Y, Yao F, A L, Lan X, et al. Ginsenoside Compound K suppresses the hepatic gluconeogenesis via activating adenosine-5′monophosphate kinase: A study in vitro and in vivo. Life Sciences. 2015;139:8-15.（doi:<https://doi.org/10.1016/j.lfs.2015.07.032>）

18. Gu J, Li W, Xiao D, Wei S, Cui W, Chen W, et al. Compound K, a final intestinal metabolite of ginsenosides, enhances insulin secretion in MIN6 pancreatic β-cells by upregulation of GLUT2. Fitoterapia. 2013;87:84-8.（doi:10.1016/j.fitote.2013.03.020）

19. Li W, Zhang M, Gu J, Meng Z-j, Zhao L-C, Zheng Y-n, et al. Hypoglycemic effect of protopanaxadiol-type ginsenosides and compound K on Type 2 Diabetes mice induced by High-Fat Diet combining with Streptozotocin via suppression of hepatic gluconeogenesis. Fitoterapia. 2012;83(1):192-8.（doi:<https://doi.org/10.1016/j.fitote.2011.10.011>）

20. Xu J, Dong J, Ding H, Wang B, Wang Y, Qiu Z, et al. Ginsenoside compound K inhibits obesity-induced insulin resistance by regulation of macrophage recruitment and polarization via activating PPARγ. Food Funct. 2022;13(6):3561-71.（doi:10.1039/d1fo04273d）

21. Jiang S, Ren D, Li J, Yuan G, Li H, Xu G, et al. Effects of compound K on hyperglycemia and insulin resistance in rats with type 2 diabetes mellitus. Fitoterapia. 2014;95:58-64.（doi:<https://doi.org/10.1016/j.fitote.2014.02.017>）

22. Wang W, Zhan W, Liang M, Huang Y, Liu Y, Wang L, et al. Ginsenoside Rb1 ameliorates the abnormal hepatic glucose metabolism by activating STAT3 in T2DM mice. Journal of Functional Foods. 2023;104:105534.（doi:<https://doi.org/10.1016/j.jff.2023.105534>）

23. Liang M, Zhan W, Wang L, Bei W, Wang W. Ginsenoside Rb1 Promotes Hepatic Glycogen Synthesis to Ameliorate T2DM Through 15-PGDH/PGE(2)/EP4 Signaling Pathway. Diabetes Metab Syndr Obes. 2023;16:3223-34.（doi:10.2147/dmso.S431423）

24. Shang W-b, Guo C, Zhao J, Yu X-z, Zhang H. [Ginsenoside Rb1 upregulates expressions of GLUTs to promote glucose consumption in adiopcytes]. Zhongguo Zhong Yao Za Zhi. 2014;39(22):4448-52

25. Liang L, Liu X, Shao J, Shen J, Yao Y, Huang X, et al. Identification of Potential α-Glucosidase Inhibitors from American Ginseng Processed Products by UHPLC-Q-Orbitrap/MS and Molecular Docking. Food Biophysics. 2024;19(3):688-700.（doi:10.1007/s11483-024-09860-8）

26. Dai S, Hong Y, Xu J, Lin Y, Si Q, Gu X. Ginsenoside Rb2 promotes glucose metabolism and attenuates fat accumulation via AKT-dependent mechanisms. Biomedicine & Pharmacotherapy. 2018;100:93-100.（doi:<https://doi.org/10.1016/j.biopha.2018.01.111>）

27. Wang Y, Fu W, Xue Y, Lu Z, Li Y, Yu P, et al. Ginsenoside Rc Ameliorates Endothelial Insulin Resistance via Upregulation of Angiotensin-Converting Enzyme 2. Front Pharmacol. 2021;12:620524.（doi:10.3389/fphar.2021.620524）

28. Han Z, Yu H, Liu Z, Zhong Y, Chang S, Sun X, et al. The hypoglycemic effect of ginsenoside Rd in db/db mice is mediated by increased insulin sensitivity and suppressed hepatic gluconeogenesis. Journal of Functional Foods. 2024;122:106475.（doi:<https://doi.org/10.1016/j.jff.2024.106475>）

29. Lin E, Wang Y, Mehendale S, Sun S, Wang CZ, Xie JT, et al. Antioxidant protection by American ginseng in pancreatic beta-cells. Am J Chin Med. 2008;36(5):981-8.（doi:10.1142/s0192415x08006399）

30. Chen J, Zhu G, Xiao W, Huang X, Wang K, Zong Y. Ginsenoside Rg1 Ameliorates Pancreatic Injuries via the AMPK/mTOR Pathway in vivo and in vitro. Diabetes Metab Syndr Obes. 2023;16:779-94.（doi:10.2147/dmso.S401642）

31. Yuan H-D, Kim DY, Quan H-Y, Kim SJ, Jung MS, Chung SH. Ginsenoside Rg2 induces orphan nuclear receptor SHP gene expression and inactivates GSK3β via AMP-activated protein kinase to inhibit hepatic glucose production in HepG2 cells. Chemico-Biological Interactions. 2012;195(1):35-42.（doi:<https://doi.org/10.1016/j.cbi.2011.10.006>）

32. Park MW, Ha J, Chung SH. 20(S)-ginsenoside Rg3 enhances glucose-stimulated insulin secretion and activates AMPK. Biol Pharm Bull. 2008;31(4):748-51.（doi:10.1248/bpb.31.748）

33. Kim KS, Jung Yang H, Lee IS, Kim KH, Park J, Jeong HS, et al. The aglycone of ginsenoside Rg3 enables glucagon-like peptide-1 secretion in enteroendocrine cells and alleviates hyperglycemia in type 2 diabetic mice. Sci Rep. 2015;5:18325.（doi:10.1038/srep18325）

34. Kim SS, Jang HJ, Oh MY, Eom DW, Kang KS, Kim YJ, et al. Ginsenoside Rg3 Enhances Islet Cell Function and Attenuates Apoptosis in Mouse Islets. Transplantation Proceedings. 2014;46(4):1150-5.（doi:<https://doi.org/10.1016/j.transproceed.2013.12.028>）

35. Zhang C, Yu H, Ye J, Tong H, Wang M, Sun G. Ginsenoside Rg3 Protects against Diabetic Cardiomyopathy and Promotes Adiponectin Signaling via Activation of PPAR-γ. Int J Mol Sci. 2023;24(23).（doi:10.3390/ijms242316736）

36. Wang H, Wu W, Wang G, Xu W, Zhang F, Wu B, et al. Protective effect of ginsenoside Rg3 on lung injury in diabetic rats. J Cell Biochem. 2019;120(3):3323-30.（doi:10.1002/jcb.27601）

37. Kim M, Ahn BY, Lee JS, Chung SS, Lim S, Park SG, et al. The ginsenoside Rg3 has a stimulatory effect on insulin signaling in L6 myotubes. Biochemical and Biophysical Research Communications. 2009;389(1):70-3.（doi:<https://doi.org/10.1016/j.bbrc.2009.08.088>）

38. Kim MJ, Koo YD, Kim M, Lim S, Park YJ, Chung SS, et al. Rg3 Improves Mitochondrial Function and the Expression of Key Genes Involved in Mitochondrial Biogenesis in C2C12 Myotubes. Diabetes Metab J. 2016;40(5):406-13.（doi:10.4093/dmj.2016.40.5.406）

39. Lee OH, Lee HH, Kim JH, Lee BY. Effect of ginsenosides Rg3 and Re on glucose transport in mature 3T3-L1 adipocytes. Phytother Res. 2011;25(5):768-73.（doi:10.1002/ptr.3322）

40. Li W-l, Li K, Chang W-g, Shi H, Zhang W-x, Wang Z, et al. 20(R)-ginsenoside Rg3 alleviates diabetic retinal injury in T2DM mice by attenuating ROS-mediated ER stress through the activation of the Nrf2/HO-1 axis. Phytomedicine. 2024;135:156202.（doi:<https://doi.org/10.1016/j.phymed.2024.156202>）

41. Xiao N, Lou MD, Lu YT, Yang LL, Liu Q, Liu B, et al. Ginsenoside Rg5 attenuates hepatic glucagon response via suppression of succinate-associated HIF-1α induction in HFD-fed mice. Diabetologia. 2017;60(6):1084-93.（doi:10.1007/s00125-017-4238-y）

42. Wei Y, Yang H, Zhu C, Deng J, Fan D. Hypoglycemic Effect of Ginsenoside Rg5 Mediated Partly by Modulating Gut Microbiota Dysbiosis in Diabetic db/db Mice. J Agric Food Chem. 2020;68(18):5107-17.（doi:10.1021/acs.jafc.0c00605）

43. Wei Y, Yang H, Zhu C, Deng J, Fan D. Ginsenoside Rg5 relieves type 2 diabetes by improving hepatic insulin resistance in db/db mice. Journal of Functional Foods. 2020;71:104014.（doi:<https://doi.org/10.1016/j.jff.2020.104014>）

44. Zhu Y, Yang H, Deng J, Fan D. Ginsenoside Rg5 Improves Insulin Resistance and Mitochondrial Biogenesis of Liver via Regulation of the Sirt1/PGC-1α Signaling Pathway in db/db Mice. J Agric Food Chem. 2021;69(30):8428-39.（doi:10.1021/acs.jafc.1c02476）

45. Li XZ, Cheng LZ, Yan YM, Liu BH, Cheng YX. SIRT1 inhibitory compounds from the roots of Codonopsis pilosula. J Asian Nat Prod Res. 2019;21(1):25-32.（doi:10.1080/10286020.2017.1422491）

46. Yang N, Zhang S, Yang S, Guo Z, Zhang X, Zhao Y. The inhibition of α-glycosidase and protein tyrosine phosphatase 1B (PTP1B) activities by ginsenosides from Panax ginseng C.A. Meyer and simultaneous determination by HPLC-ELSD. Journal of Functional Foods. 2016;23:188-97.（doi:<https://doi.org/10.1016/j.jff.2015.12.018>）

47. Wang L-Y, Sun H-Q, Zhang X-W, Zhang W, Zhu Z-Y. Derivatives of glycyrrhetinic acid with amino acid: Modifications, structural characterization, hypoglycemic activity and mechanism. Process Biochemistry. 2023;134:186-98.（doi:<https://doi.org/10.1016/j.procbio.2023.10.005>）

48. Li Z-Y, Tung Y-T, Chen S-Y, Yen G-C. Novel findings of 18β-glycyrrhetinic acid on sRAGE secretion through inhibition of transient receptor potential canonical channels in high-glucose environment. BioFactors. 2019;45(4):607-15.（doi:<https://doi.org/10.1002/biof.1517>）

49. Yang M, Zhang M, Liu Q, Xu T, Huang T, Yao D, et al. 18β-Glycyrrhetinic acid acts through hepatocyte nuclear factor 4 alpha to modulate lipid and carbohydrate metabolism. Pharmacol Res. 2020;157:104840.（doi:10.1016/j.phrs.2020.104840）

50. Zhang W, Li T, Zhang XJ, Zhu ZY. Hypoglycemic effect of glycyrrhizic acid, a natural non-carbohydrate sweetener, on streptozotocin-induced diabetic mice. Food Funct. 2020;11(5):4160-70.（doi:10.1039/c9fo02114k）

51. Akutagawa K, Fujita T, Ouhara K, Takemura T, Tari M, Kajiya M, et al. Glycyrrhizic acid suppresses inflammation and reduces the increased glucose levels induced by the combination of Porphyromonas gulae and ligature placement in diabetic model mice. International Immunopharmacology. 2019;68:30-8.（doi:<https://doi.org/10.1016/j.intimp.2018.12.045>）

52. KO B-S, JANG JS, HONG SM, SUNG SR, LEE JE, LEE MY, et al. Changes in Components, Glycyrrhizin and Glycyrrhetinic Acid, in Raw Glycyrrhiza uralensis Fisch, Modify Insulin Sensitizing and Insulinotropic Actions. Bioscience, Biotechnology, and Biochemistry. 2007;71(6):1452-61.（doi:10.1271/bbb.60533）

53. Zhong Y, Luo R, Liu Q, Zhu J, Lei M, Liang X, et al. Jujuboside A ameliorates high fat diet and streptozotocin induced diabetic nephropathy via suppressing oxidative stress, apoptosis, and enhancing autophagy. Food and Chemical Toxicology. 2022;159:112697.（doi:<https://doi.org/10.1016/j.fct.2021.112697>）

54. Liu Z, Qu CY, Li JX, Wang YF, Li W, Wang CZ, et al. Hypoglycemic and Hypolipidemic Effects of Malonyl Ginsenosides from American Ginseng (Panax quinquefolius L.) on Type 2 Diabetic Mice. ACS Omega. 2021;6(49):33652-64.（doi:10.1021/acsomega.1c04656）

55. Gao H, Wu H. Maslinic acid activates renal AMPK/SIRT1 signaling pathway and protects against diabetic nephropathy in mice. BMC Endocrine Disorders. 2022;22(1):25.（doi:10.1186/s12902-022-00935-6）

56. Zhang Y, Zhou G, Peng Y, Wang M, Li X. Anti-hyperglycemic and anti-hyperlipidemic effects of a special fraction of Luohanguo extract on obese T2DM rats. J Ethnopharmacol. 2020;247:112273.（doi:10.1016/j.jep.2019.112273）

57. Suzuki YA, Murata Y, Inui H, Sugiura M, Nakano Y. Triterpene Glycosides of Siraitia grosvenori Inhibit Rat Intestinal Maltase and Suppress the Rise in Blood Glucose Level after a Single Oral Administration of Maltose in Rats. Journal of Agricultural and Food Chemistry. 2005;53(8):2941-6.（doi:10.1021/jf0478105）

58. Huang Y-C, Chang W-L, Huang S-F, Lin C-Y, Lin H-C, Chang T-C. Pachymic acid stimulates glucose uptake through enhanced GLUT4 expression and translocation. European Journal of Pharmacology. 2010;648(1):39-49.（doi:<https://doi.org/10.1016/j.ejphar.2010.08.021>）

59. Shen Q, Qi S-m, Zhang J-t, Li M-h, Wang Y-p, Wang Z, et al. Platycodin D inhibits HFD/STZ-induced diabetic nephropathy via inflammatory and apoptotic signaling pathways in C57BL/6 mice. Journal of Ethnopharmacology. 2023;314:116596.（doi:<https://doi.org/10.1016/j.jep.2023.116596>）

60. Guo C, Li C, Yu Y, Chen W, Ma T, Zhou Z. Antihyperglycemic and antihyperlipidemic activities of protodioscin in a high-fat diet and streptozotocin-induced diabetic rats. RSC Advances. 2016;6(91):88640-6.（doi:10.1039/C6RA18448K）

61. Wu G, Yi J, Liu L, Wang P, Zhang Z, Li Z. Pseudoginsenoside F11, a Novel Partial PPAR γ Agonist, Promotes Adiponectin Oligomerization and Secretion in 3T3-L1 Adipocytes. PPAR Res. 2013;2013:701017.（doi:10.1155/2013/701017）

62. He K, Song S, Zou Z, Feng M, Wang D, Wang Y, et al. The Hypoglycemic and Synergistic Effect of Loganin, Morroniside, and Ursolic Acid Isolated from the Fruits of Cornus officinalis. Phytotherapy Research. 2016;30(2):283-91.（doi:<https://doi.org/10.1002/ptr.5529>）

63. Wang J, Zhao J, Yan Y, Liu D, Wang C, Wang H. Inhibition of glycosidase by ursolic acid: in vitro, in vivo and in silico study. Journal of the Science of Food and Agriculture. 2020;100(3):986-94.（doi:<https://doi.org/10.1002/jsfa.10098>）

64. Tang S, Fang C, Liu Y, Tang L, Xu Y. Anti-obesity and Anti-diabetic Effect of Ursolic Acid against Streptozotocin/High Fat Induced Obese in Diabetic Rats. J Oleo Sci. 2022;71(2):289-300.（doi:10.5650/jos.ess21258）

65. Dai S, Meng X, Cai X, Yuan C, Zhao Z, Zhong L, et al. Therapeutic effect of ursolic acid on fetal development in pregnant rats with gestational diabetes mellitus via AGEs-RAGE signaling pathway. Journal of Food Biochemistry. 2021;45(4):e13651.（doi:<https://doi.org/10.1111/jfbc.13651>）

66. Lo SH, Li Y, Cheng KC, Niu CS, Cheng JT, Niu HS. Ursolic acid activates the TGR5 receptor to enhance GLP-1 secretion in type 1-like diabetic rats. Naunyn Schmiedebergs Arch Pharmacol. 2017;390(11):1097-104.（doi:10.1007/s00210-017-1409-9）

67. Jang S-M, Yee S-T, Choi J, Choi M-S, Do G-M, Jeon S-M, et al. Ursolic acid enhances the cellular immune system and pancreatic β-cell function in streptozotocin-induced diabetic mice fed a high-fat diet. International Immunopharmacology. 2009;9(1):113-9.（doi:<https://doi.org/10.1016/j.intimp.2008.10.013>）

68. Lee J, Yee S-T, Kim J-J, Choi M-S, Kwon E-Y, Seo K-I, et al. Ursolic acid ameliorates thymic atrophy and hyperglycemia in streptozotocin–nicotinamide-induced diabetic mice. Chemico-Biological Interactions. 2010;188(3):635-42.（doi:<https://doi.org/10.1016/j.cbi.2010.09.019>）

69. Lin M-H, Liu H-K, Huang W-J, Huang C-C, Wu T-H, Hsu F-L. Evaluation of the Potential Hypoglycemic and Beta-Cell Protective Constituents Isolated from Corni Fructus To Tackle Insulin-Dependent Diabetes Mellitus. Journal of Agricultural and Food Chemistry. 2011;59(14):7743-51.（doi:10.1021/jf201189r）

70. Zhou X, Liang J, Zhang Y, Zhao H, Guo Y, Shi S. Separation and purification of α-glucosidase inhibitors from Polygonatum odoratum by stepwise high-speed counter-current chromatography combined with Sephadex LH-20 chromatography target-guided by ultrafiltration-HPLC screening. J Chromatogr B Analyt Technol Biomed Life Sci. 2015;985:149-54.（doi:10.1016/j.jchromb.2015.01.030）

71. Chen L, Li QY, Shi XJ, Mao SL, Du YL. 6-Hydroxydaidzein enhances adipocyte differentiation and glucose uptake in 3T3-L1 cells. J Agric Food Chem. 2013;61(45):10714-9.（doi:10.1021/jf402694m）

72. Ma Q, Wei R, Liu W. China Journal of Chinese Materia Medica. China Journal of Chinese Materia Medica. 2016;41(05):868-73.（doi:10.4268/cjcmm20160518）

73. Guo H, Zhao H, Kanno Y, Li W, Mu Y, Kuang X, et al. A dihydrochalcone and several homoisoflavonoids from Polygonatum odoratum are activators of adenosine monophosphate-activated protein kinase. Bioorganic & Medicinal Chemistry Letters. 2013;23(11):3137-9.（doi:<https://doi.org/10.1016/j.bmcl.2013.04.027>）

74. Wang H, Fowler MI, Messenger DJ, Terry LA, Gu X, Zhou L, et al. Homoisoflavonoids Are Potent Glucose Transporter 2 (GLUT2) Inhibitors: A Potential Mechanism for the Glucose-Lowering Properties of Polygonatum odoratum. J Agric Food Chem. 2018;66(12):3137-45.（doi:10.1021/acs.jafc.8b00107）

75. Zhu Y, Yang Y, Tang Z, Xue T, Chen S, Yang H, et al. UHPLC–PDA–Q-TOF–MS–α-amylase–FLD activity detection system and molecular docking. Phytochemical Analysis. 2024;35(6):1457-71.（doi:<https://doi.org/10.1002/pca.3377>）

76. Xiang H, Xu P, Wen W, Qiu H, Chu C, Shao Q, et al. Screening, characterization of trace α-glucosidase inhibitors from the root of Pueraria lobata and evaluation of their hypoglycemic activity. Food Bioscience. 2023;53:102641.（doi:<https://doi.org/10.1016/j.fbio.2023.102641>）

77. Jiang W, Kan H, Li P, Liu S, Liu Z. Screening and structural characterization of potential α-glucosidase inhibitors from Radix Astragali flavonoids extract by ultrafiltration LC-DAD-ESI-MSn. Analytical Methods. 2015;7(1):123-8.（doi:10.1039/C4AY02081B）

78. Chen QC, Zhang WY, Jin W, Lee IS, Min BS, Jung HJ, et al. Flavonoids and isoflavonoids from Sophorae Flos improve glucose uptake in vitro. Planta Med. 2010;76(1):79-81.（doi:10.1055/s-0029-1185944）

79. Li Y, Duan B, Li Y, Yu S, Wang Y. The isoflavonoid calycosin inhibits inflammation and enhances beta cell function in gestational diabetes mellitus by suppressing RNF38 expression. Immunopharmacol Immunotoxicol. 2020;42(4):366-72.（doi:10.1080/08923973.2020.1782426）

80. Xu H, Wang C, Gong L. Hypoglycemic activity in vivo and in vitro of the Lotus (Nelumbo nucifera Gaertn.) seed skin (testa) phenolic-rich extracts. Food Chemistry: X. 2024;22:101282.（doi:<https://doi.org/10.1016/j.fochx.2024.101282>）

81. Huang CF, Chen YW, Yang CY, Lin HY, Way TD, Chiang W, et al. Extract of Lotus Leaf (Nelumbo nucifera) and Its Active Constituent Catechin with Insulin Secretagogue Activity. Journal of Agricultural and Food Chemistry. 2011;59(4):1087-94.（doi:10.1021/jf103382h）

82. Fan H, Chen M, Dai T, Deng L, Liu C, Zhou W, et al. Phenolic compounds profile of Amomum tsaoko Crevost et Lemaire and their antioxidant and hypoglycemic potential. Food Bioscience. 2023;52:102508.（doi:<https://doi.org/10.1016/j.fbio.2023.102508>）

83. Lee J, Lee S, Kim B, Seo WD, Jia Y, Wu C, et al. Barley sprout extract containing policosanols and polyphenols regulate AMPK, SREBP2 and ACAT2 activity and cholesterol and glucose metabolism in vitro and in vivo. Food Research International. 2015;72:174-83.（doi:10.1016/j.foodres.2015.03.041）

84. Wang X-L, Jiao F-R, Yu M, Lin L-B, Xiao J, Zhang Q, et al. Constituents with potent α-glucosidase inhibitory activity from Pueraria lobata (Willd.) ohwi. Bioorganic & Medicinal Chemistry Letters. 2017;27(9):1993-8.（doi:<https://doi.org/10.1016/j.bmcl.2017.03.013>）

85. Kwon DY, Hong SM, Ahn IS, Kim MJ, Yang HJ, Park S. Isoflavonoids and peptides from meju, long-term fermented soybeans, increase insulin sensitivity and exert insulinotropic effects in vitro. Nutrition. 2011;27(2):244-52.（doi:<https://doi.org/10.1016/j.nut.2010.02.004>）

86. Kuroda M, Mimaki Y, Sashida Y, Mae T, Kishida H, Nishiyama T, et al. Phenolics with PPAR-γ ligand-Binding activity obtained from licorice (Glycyrrhiza uralensis Roots) and ameliorative effects of glycyrin on genetically diabetic KK-Ay mice. Bioorganic & Medicinal Chemistry Letters. 2003;13(24):4267-72.（doi:<https://doi.org/10.1016/j.bmcl.2003.09.052>）

87. Zhang J, Zhang L, Lai C, Liang Y, Gao L, Kaliaperumal K, et al. Nutraceutical potential of navel orange peel in diabetes management: The chemical profile, antioxidant, α-glucosidase inhibitory and antiglycation effects of its flavonoids. Food Bioscience. 2022;49:101943.（doi:<https://doi.org/10.1016/j.fbio.2022.101943>）

88. Meephat S, Prasatthong P, Rattanakanokchai S, Bunbupha S, Maneesai P, Pakdeechote P. Diosmetin attenuates metabolic syndrome and left ventricular alterations via the suppression of angiotensin II/AT1 receptor/gp(91phox)/p-NF-κB protein expression in high-fat diet fed rats. Food Funct. 2021;12(4):1469-81.（doi:10.1039/d0fo02744h）

89. Yu L, Shirai N, Suzuki H, Sugane N, Hosono T, Nakajima Y, et al. The effect of methanol extracts of tsao-ko (Amomum tsao-ko Crevost et Lemaire) on digestive enzyme and antioxidant activity in vitro, and plasma lipids and glucose and liver lipids in mice. J Nutr Sci Vitaminol (Tokyo). 2010;56(3):171-6.（doi:10.3177/jnsv.56.171）

90. Dkhar B, Khongsti K, Thabah D, Syiem D, Satyamoorthy K, Das B. Genistein represses PEPCK-C expression in an insulin-independent manner in HepG2 cells and in alloxan-induced diabetic mice. J Cell Biochem. 2018;119(2):1953-70.（doi:10.1002/jcb.26356）

91. Fu Z, Zhang W, Zhen W, Lum H, Nadler J, Bassaganya-Riera J, et al. Genistein Induces Pancreatic β-Cell Proliferation through Activation of Multiple Signaling Pathways and Prevents Insulin-Deficient Diabetes in Mice. Endocrinology. 2010;151(7):3026-37.（doi:10.1210/en.2009-1294）

92. Nga VT, Hao HM. Inhibition kinetics and mechanism of genistein against α-glucosidase. Vietnam Journal of Chemistry. 2024;62(4):493-9.（doi:<https://doi.org/10.1002/vjch.202200173>）

93. Li D, Fan J, Du L, Ren G. Prenylated flavonoid fractions from Glycyrrhiza glabra alleviate insulin resistance in HepG2 cells by regulating the ERK/IRS-1 and PI3K/Akt signaling pathways. Archives of Pharmacal Research. 2024;47(2):127-45.（doi:10.1007/s12272-024-01485-2）

94. Wu F, Jin Z, Jin J. Hypoglycemic effects of glabridin, a polyphenolic flavonoid from licorice, in an animal model of diabetes mellitus. Mol Med Rep. 2013;7(4):1278-82.（doi:10.3892/mmr.2013.1330）

95. Feng C, Yu W, Gang Q. Research on Structure Identification of Glycitin from Glycyrrhiza and Its Inhibitory Activity Against α-Glucosidase. Science & Technology Review. 2014;32(16):29-33.（doi:10.7666/d.Y917502）

96. Lim HJ, Park JE, Han JS. HM-chromanone from Portulaca oleracea L. inhibits protein tyrosine phosphatase 1B and mitigates glucose production in insulin-resistant HepG2 cells. Fitoterapia. 2023;167:105512.（doi:<https://doi.org/10.1016/j.fitote.2023.105512>）

97. Lin YT, Lin HR, Yang CS, Liaw CC, Sung PJ, Kuo YH, et al. Antioxidant and Anti-α-Glucosidase Activities of Various Solvent Extracts and Major Bioactive Components from the Fruits of Crataegus pinnatifida. Antioxidants (Basel). 2022;11(2).（doi:10.3390/antiox11020320）

98. Gong Y, Li J, Li J, Wang L, Fan L. In Vitro Inhibitory Effects of Polyphenols from Flos sophorae immaturus on α-Glucosidase: Action Mechanism, Isothermal Titration Calorimetry and Molecular Docking Analysis. Foods. 2023;12(4):715

99. Zhang Y, Wang M, Dong H, Yu X, Zhang J. Anti-hypoglycemic and hepatocyte-protective effects of hyperoside from Zanthoxylum bungeanum leaves in mice with high-carbohydrate/high-fat diet and alloxan-induced diabetes. Int J Mol Med. 2018;41(1):77-86.（doi:10.3892/ijmm.2017.3211）

100. Han，Fenxia., Fang J, Geng S. Inhibitory Mechanism of Isoliquiritigenin against α-Glucosidase. Food Science. 2019;40(15):37-42

101. Li J-s, Ji T, Su S-l, Zhu Y, Chen X-l, Shang E-x, et al. Mulberry leaves ameliorate diabetes via regulating metabolic profiling and AGEs/RAGE and p38 MAPK/NF-κB pathway. Journal of Ethnopharmacology. 2022;283:114713.（doi:<https://doi.org/10.1016/j.jep.2021.114713>）

102. Bai X, Zhao X, Liu K, Yang X, He Q, Gao Y, et al. Mulberry Leaf Compounds and Gut Microbiota in Alzheimer's Disease and Diabetes: A Study Using Network Pharmacology, Molecular Dynamics Simulation, and Cellular Assays. Int J Mol Sci. 2024;25(7).（doi:10.3390/ijms25074062）

103. Wang J, Lu CS, Liu DY, Xu YT, Zhu Y, Wu HH. Constituents from Polygonatum sibiricum and their inhibitions on the formation of advanced glycosylation end products. J Asian Nat Prod Res. 2016;18(7):697-704.（doi:10.1080/10286020.2015.1135905）

104. Luo Z, Fu C, Li T, Gao Q, Miao D, Xu J, et al. Hypoglycemic Effects of Licochalcone A on the Streptozotocin-Induced Diabetic Mice and Its Mechanism Study. J Agric Food Chem. 2021;69(8):2444-56.（doi:10.1021/acs.jafc.0c07630）

105. Wang Z, Bai Z, Yan J, Liu T, Li Y, Xu J, et al. Anti-diabetic effects of linarin from Chrysanthemi Indici Flos via AMPK activation. Chinese Herbal Medicines. 2022;14(1):97-103.（doi:<https://doi.org/10.1016/j.chmed.2021.11.002>）

106. Carnovali M, Luzi L, Terruzzi I, Banfi G, Mariotti M. Liquiritigenin Reduces Blood Glucose Level and Bone Adverse Effects in Hyperglycemic Adult Zebrafish. Nutrients. 2019;11(5):1042

107. Zhao Q, Yang J, Li J, Zhang L, Yan X, Yue T, et al. Hypoglycemic effect and intestinal transport of phenolics-rich extract from digested mulberry leaves in Caco-2/insulin-resistant HepG2 co-culture model. Food Research International. 2024;175:113689.（doi:<https://doi.org/10.1016/j.foodres.2023.113689>）

108. Chen L, Tian G, Tang W, Luo W, Liu P, Ma Z. Protective effect of luteolin on streptozotocin-induced diabetic renal damage in mice via the regulation of RIP140/NF-кB pathway and insulin signalling pathway. Journal of Functional Foods. 2016;22:93-100.（doi:<https://doi.org/10.1016/j.jff.2016.01.023>）

109. Ahmed OM, Hassan MA, Abdel-Twab SM, Abdel Azeem MN. Navel orange peel hydroethanolic extract, naringin and naringenin have anti-diabetic potentials in type 2 diabetic rats. Biomedicine & Pharmacotherapy. 2017;94:197-205.（doi:<https://doi.org/10.1016/j.biopha.2017.07.094>）

110. Okesina KB, Odetayo AF, Adeyemi WJ, Okesina AA, Bassey GE, Olayaki LA. Naringin Prevents Diabetic-Induced Dysmetabolism in Male Wistar Rats by Modulating GSK-3 Activities and Oxidative Stress-Dependent Pathways. Cell Biochemistry and Biophysics. 2024;82(4):3559-71.（doi:10.1007/s12013-024-01444-0）

111. Yuan S, Ye Z, Li Y, Zou J, Wu M, Wang K, et al. Hypoglycemic Effect of Nobiletin via Regulation of Islet β-Cell Mitophagy and Gut Microbiota Homeostasis in Streptozocin-Challenged Mice. J Agric Food Chem. 2022;70(19):5805-18.（doi:10.1021/acs.jafc.2c00148）

112. Wang C, Yao J, Ju L, Wen X, Shu L. Puerarin ameliorates hyperglycemia in HFD diabetic mice by promoting β-cell neogenesis via GLP-1R signaling activation. Phytomedicine. 2020;70:153222.（doi:<https://doi.org/10.1016/j.phymed.2020.153222>）

113. Liu S, Yu J, Fu M, Wang X, Chang X. Regulatory effects of hawthorn polyphenols on hyperglycemic, inflammatory, insulin resistance responses, and alleviation of aortic injury in type 2 diabetic rats. Food Research International. 2021;142:110239.（doi:<https://doi.org/10.1016/j.foodres.2021.110239>）

114. Hunyadi A, Martins A, Hsieh TJ, Seres A, Zupkó I. Chlorogenic acid and rutin play a major role in the in vivo anti-diabetic activity of Morus alba leaf extract on type II diabetic rats. PLoS One. 2012;7(11):e50619.（doi:10.1371/journal.pone.0050619）

115. Xu W, Miyamoto L, Aihara H, Yamaoka T, Tanaka N, Tsuchihashi Y, et al. Methanol extraction fraction from Citrus Sudachi peel exerts lipid reducing effects in cultured cells. J Med Invest. 2018;65(3.4):225-30.（doi:10.2152/jmi.65.225）

116. Tsutsumi R, Yoshida T, Nii Y, Okahisa N, Iwata S, Tsukayama M, et al. Sudachitin, a polymethoxylated flavone, improves glucose and lipid metabolism by increasing mitochondrial biogenesis in skeletal muscle. Nutr Metab (Lond). 2014;11:32.（doi:10.1186/1743-7075-11-32）

117. Guo J, Chen J, Ren W, Zhu Y, Zhao Q, Zhang K, et al. Citrus flavone tangeretin is a potential insulin sensitizer targeting hepatocytes through suppressing MEK-ERK1/2 pathway. Biochem Biophys Res Commun. 2020;529(2):277-82.（doi:10.1016/j.bbrc.2020.05.212）

118. Sundaram R, Shanthi P, Sachdanandam P. Effect of tangeretin, a polymethoxylated flavone on glucose metabolism in streptozotocin-induced diabetic rats. Phytomedicine. 2014;21(6):793-9.（doi:<https://doi.org/10.1016/j.phymed.2014.01.007>）

119. Huang S-S, Yan Y-H, Ko C-H, Chen K-M, Lee S-C, Liu C-T. A Comparison of Food-grade Folium mori Extract and 1-Deoxynojirimycin for Glycemic Control and Renal Function in Streptozotocin-induced Diabetic Rats. Journal of Traditional and Complementary Medicine. 2014;4(3):162-70.（doi:<https://doi.org/10.4103/2225-4110.131639>）

120. Ma C, Wang J, Chu H, Zhang X, Wang Z, Wang H, et al. Purification and Characterization of Aporphine Alkaloids from Leaves of Nelumbo nucifera Gaertn and Their Effects on Glucose Consumption in 3T3-L1 Adipocytes. International Journal of Molecular Sciences. 2014;15(3):3481-94

121. Ma Q, Wei R, Wang Z, Liu W, Sang Z, Li Y, et al. Bioactive alkaloids from the aerial parts of Houttuynia cordata. J Ethnopharmacol. 2017;195:166-72.（doi:10.1016/j.jep.2016.11.013）

122. Huang X, Wu W, Cheng H. High-Efficiency Extraction and in Vitro Bioactivity of Amygdalin from Peach Kernel. Journal of Chinese Institute of Food Science and Technology. 2023;23(04):146-56.（doi:10.16429/j.1009-7848.2023.04.015）

123. LIU Z, Li C, Yang R. Separation of Water-soluble Constitituents in Polygonati OdoratiＲhizoma and Their Glucosidase Inhibitory Activity. Chinese Journal of Experimental Traditional Medical Formulae. 2016;22(18):51-5.（doi:10.13422/j.cnki.syfjx.2016180051）

124. Kato E, Inagaki Y, Kawabata J. Higenamine 4′-O-β-d-glucoside in the lotus plumule induces glucose uptake of L6 cells through β2-adrenergic receptor. Bioorganic & Medicinal Chemistry. 2015;23(13):3317-21.（doi:<https://doi.org/10.1016/j.bmc.2015.04.054>）

125. Wei X, Yang B, Chen X, Wen L, Kan J. Zanthoxylum alkylamides ameliorate protein metabolism in type 2 diabetes mellitus rats by regulating multiple signaling pathways. Food Funct. 2021;12(8):3740-53.（doi:10.1039/d0fo02695f）

126. Li W, Wu Y, Liu Y, Tang Y, Che Z, Wu T. Chemical profiles and screening of potential α-glucosidase inhibitors from Sichuan pepper using ultra-filtration combined with UHPLC-Q-TOF. Industrial Crops and Products. 2020;143:111874.（doi:<https://doi.org/10.1016/j.indcrop.2019.111874>）

127. Zhang Q, Li RL, Wang LY, Zhang T, Qian D, Tang DD, et al. Hydroxy-α-sanshool isolated from Zanthoxylum bungeanum Maxim. has antidiabetic effects on high-fat-fed and streptozotocin-treated mice via increasing glycogen synthesis by regulation of PI3K/Akt/GSK-3β/GS signaling. Front Pharmacol. 2022;13:1089558.（doi:10.3389/fphar.2022.1089558）

128. Li YY, Stewart DA, Ye XM, Yin LH, Pathmasiri WW, McRitchie SL, et al. A Metabolomics Approach to Investigate Kukoamine B-A Potent Natural Product With Anti-diabetic Properties. Front Pharmacol. 2018;9:1575.（doi:10.3389/fphar.2018.01575）

129. Hu，Mengya, FANG Y, Xu，Ruiwen, Sun，Yanjun, Dong，Chengming, Fen，Weishen, et al. Study on the Chemical Constituents of Lycium chinense var.potaninii and

α-Glucosidase Inhibitory Activity. Journal of Chinese Medicinal Materials. 2024;47(03):624-8.（doi:10.13863/j.issn1001-4454.2024.03.016）

130. Wang J, Dong Y, Li Q. Neferine induces mitochondrial dysfunction to exert anti-proliferative and anti-invasive activities on retinoblastoma. Exp Biol Med (Maywood). 2020;245(15):1385-94.（doi:10.1177/1535370220928933）

131. Zhang H, Chen G, Zhang Y, Yang M, Chen J, Guo M. Potential hypoglycemic, hypolipidemic, and anti-inflammatory bioactive components in Nelumbo nucifera leaves explored by bioaffinity ultrafiltration with multiple targets. Food Chemistry. 2022;375:131856.（doi:<https://doi.org/10.1016/j.foodchem.2021.131856>）

132. Pan Y, Cai B, Wang K, Wang S, Zhou S, Yu X, et al. Neferine enhances insulin sensitivity in insulin resistant rats. Journal of Ethnopharmacology. 2009;124(1):98-102.（doi:<https://doi.org/10.1016/j.jep.2009.04.008>）

133. Guan G, Han H, Yang Y, Jin Y, Wang X, Liu X. Neferine prevented hyperglycemia-induced endothelial cell apoptosis through suppressing ROS/Akt/NF-κB signal. Endocrine. 2014;47(3):764-71.（doi:10.1007/s12020-014-0186-1）

134. Liu S, Li G, Tang X. Effects of neferine on type 2 diabetic mellitus rats by metabonomics method. Chinese Pharmacological Bulletin. 2012;28(04):490-5

135. Ma C, Li G, He Y, Xu B, Mi X, Wang H, et al. Pronuciferine and nuciferine inhibit lipogenesis in 3T3-L1 adipocytes by activating the AMPK signaling pathway. Life Sci. 2015;136:120-5.（doi:10.1016/j.lfs.2015.07.001）

136. Su W, Li Y, Chang AK, Sheng T, Pei Y, Li J, et al. Identification of Novel Alkaloids from Portulaca oleracea L. and Characterization of Their Pharmacokinetics and GLP-1 Secretion-Promoting Activity in STC-1 Cells. Journal of Agricultural and Food Chemistry. 2023;71(49):19804-16.（doi:10.1021/acs.jafc.3c05191）

137. Wang RY, Su PJ, Li B, Zhan XQ, Qi FM, Lv CW, et al. Two new aromatic derivatives from Codonopsis pilosula and their α-glucosidase inhibitory activities. Nat Prod Res. 2022;36(19):4929-35.（doi:10.1080/14786419.2021.1912749）

138. Pham HG, Ha MT, Le TT, Kim JA, Min BS. Inhibitory Activity of PTP1B and α-Glucosidase by Compounds from Whole Plants of Houttuynia cordata Thunb. Natural Product Sciences. 2023

139. Wikul A, Damsud T, Kataoka K, Phuwapraisirisan P. (+)-Pinoresinol is a putative hypoglycemic agent in defatted sesame (Sesamum indicum) seeds though inhibiting α-glucosidase. Bioorganic & Medicinal Chemistry Letters. 2012;22(16):5215-7.（doi:<https://doi.org/10.1016/j.bmcl.2012.06.068>）

140. Haddou S, Elrherabi A, Loukili EH, Abdnim R, Hbika A, Bouhrim M, et al. Chemical Analysis of the Antihyperglycemic, and Pancreatic α-Amylase, Lipase, and Intestinal α-Glucosidase Inhibitory Activities of Cannabis sativa L. Seed Extracts. Molecules. 2023;29(1).（doi:10.3390/molecules29010093）

141. Samad MB, Mohsin M, Razu BA, Hossain MT, Mahzabeen S, Unnoor N, et al. [6]-Gingerol, from Zingiber officinale, potentiates GLP-1 mediated glucose-stimulated insulin secretion pathway in pancreatic β-cells and increases RAB8/RAB10-regulated membrane presentation of GLUT4 transporters in skeletal muscle to improve hyperglycemia in Lepr(db/db) type 2 diabetic mice. BMC Complement Altern Med. 2017;17(1):395.（doi:10.1186/s12906-017-1903-0）

142. Ding M, Wu S-L, Hu J, He X-F, Huang X-Y, Li T-Z, et al. Norlignans as potent GLP-1 secretagogues from the fruits of Amomum villosum. Phytochemistry. 2022;199:113204.（doi:<https://doi.org/10.1016/j.phytochem.2022.113204>）

143. Hao X, Jin Z, Gao R, Li S, Li C, Liu L, et al. The quality control and hypoglycemic effect of Cinnamomi Cortex aqueous extract. Journal of Future Foods. 2024;4(2):167-72.（doi:<https://doi.org/10.1016/j.jfutfo.2023.06.008>）

144. Xu Y, Li J, Cui M, Li X, Zhai H, Wu D, et al. Therapeutic effects of medicinal and food-based traditional herbal couples on type 2 diabetes mellitus based on pharmacodynamics and pharmacokinetics. Front Pharmacol. 2025;16:1560271.（doi:10.3389/fphar.2025.1560271）

145. Zhou Y. The Protective Effects of Cryptochlorogenic Acid on β-Cells Function in Diabetes in vivo and vitro via Inhibition of Ferroptosis. Diabetes Metab Syndr Obes. 2020;13:1921-31.（doi:10.2147/dmso.S249382）

146. Zhang，Z., Jing G, liu Y. The hypoglycemic effect of eugenol on type 2 diabetie mice and regulationof signaling transduction pathway of glucose and lipid metabolism in liver. Chinese Pharmacological Bulletin. 2022;38(12):1875-81.（doi:10.12360/CPB202202041）

147. Srinivasan S, Sathish G, Jayanthi M, Muthukumaran J, Muruganathan U, Ramachandran V. Ameliorating effect of eugenol on hyperglycemia by attenuating the key enzymes of glucose metabolism in streptozotocin-induced diabetic rats. Molecular and Cellular Biochemistry. 2014;385(1):159-68.（doi:10.1007/s11010-013-1824-2）

148. Yoshioka Y, Kono R, Kuse M, Yamashita Y, Ashida H. Phenylpropanoids and neolignans isolated from Myristica fragrans enhance glucose uptake in myotubes. Food Funct. 2022;13(7):3879-93.（doi:10.1039/d1fo04408g）

149. Han KL, Choi JS, Lee JY, Song J, Joe MK, Jung MH, et al. Therapeutic potential of peroxisome proliferators--activated receptor-alpha/gamma dual agonist with alleviation of endoplasmic reticulum stress for the treatment of diabetes. Diabetes. 2008;57(3):737-45.（doi:10.2337/db07-0972）

150. Yang S, Min Kyun N, Jang JP, Kim KA, Kim BY, Sung NJ, et al. Inhibition of protein tyrosine phosphatase 1B by lignans from Myristica fragrans. Phytotherapy Research. 2006;20(8):680-2.（doi:<https://doi.org/10.1002/ptr.1935>）

151. Lee D, Qi Y, Kim R, Song J, Kim H, Kim HY, et al. Methyl Caffeate Isolated from the Flowers of Prunus persica (L.) Batsch Enhances Glucose-Stimulated Insulin Secretion. Biomolecules. 2021;11(2).（doi:10.3390/biom11020279）

152. Nguyen PH, Le TVT, Kang HW, Chae J, Kim SK, Kwon K-i, et al. AMP-activated protein kinase (AMPK) activators from Myristica fragrans (nutmeg) and their anti-obesity effect. Bioorganic & Medicinal Chemistry Letters. 2010;20(14):4128-31.（doi:<https://doi.org/10.1016/j.bmcl.2010.05.067>）

153. Hien TT, Oh WK, Nguyen PH, Oh SJ, Lee MY, Kang KW. Nectandrin B Activates Endothelial Nitric-Oxide Synthase Phosphorylation in Endothelial Cells: Role of the AMP-Activated Protein Kinase/Estrogen Receptor α/Phosphatidylinositol 3-kinase/Akt Pathway. Molecular Pharmacology. 2011;80(6):1166-78.（doi:<https://doi.org/10.1124/mol.111.073502>）

154. Niu Q-q, Fu Z-z, Mao B-y, Zhang X, Wang H-d, Li P, et al. Perillaldehyde targeting PARP1 to inhibit TRPM2-CaMKII/CaN signal transduction in diabetic cardiomyopathy. International Immunopharmacology. 2025;150:114291.（doi:<https://doi.org/10.1016/j.intimp.2025.114291>）

155. Park EY, Kim EH, Kim CY, Kim MH, Choung JS, Oh YS, et al. Angelica dahurica Extracts Improve Glucose Tolerance through the Activation of GPR119. PLoS One. 2016;11(7):e0158796.（doi:10.1371/journal.pone.0158796）

156. Yang，H. Effect of raspberry ketone on expression of SHP-1 and IRS- in insulin signalingpathway of HepG2 cells. Chinese Traditional Patent Medicine. 2014;36(08):1579-83

157. Sotnikova R, Okruhlicova L, Vlkovicova J, Navarova J, Gajdacova B, Pivackova L, et al. Rosmarinic acid administration attenuates diabetes-induced vascular dysfunction of the rat aorta. Journal of Pharmacy and Pharmacology. 2013;65(5):713-23.（doi:10.1111/jphp.12037）

158. Runtuwene J, Cheng KC, Asakawa A, Amitani H, Amitani M, Morinaga A, et al. Rosmarinic acid ameliorates hyperglycemia and insulin sensitivity in diabetic rats, potentially by modulating the expression of PEPCK and GLUT4. Drug Des Devel Ther. 2016;10:2193-202.（doi:10.2147/dddt.S108539）

159. Keri L, Ajeng D, Melisa IB, Tri HA, Anas S, Mutakin, et al. Potential Natural Dual Agonist PPAR&#945;/&#947;-induced Antidiabetic and Antidyslipidemic Properties of Safrole-Free Nutmeg Seed (Myristica fragrans Houtt) Extract. The Natural Products Journal. 2019;9(3):248-53.（doi:<http://dx.doi.org/10.2174/2210315509666190206122849>）

160. Huang S-M, Chuang C-H, Rejano CJF, Tayo LL, Hsieh C-Y, Huang SK-H, et al. Sesamin: A Promising Therapeutic Agent for Ameliorating Symptoms of Diabetes. Molecules. 2023;28(21):7255

161. Zheng W, Yang J, Zhang Q, Cheng M, Shaukat H, Qin H. Sesamol Alleviates High-Fat Diet-Induced Hepatic Insulin Resistance in C57BL/6 J Mice Through AMPK Activation Mediated by Adipose Adiponectin. Plant Foods for Human Nutrition. 2023;78(4):720-7.（doi:10.1007/s11130-023-01108-z）

162. Yang M, Hao Z, Wang X, Zhou S, Xiao C, Zhu D, et al. Four undescribed iridoid glycosides with antidiabetic activity from fruits of Cornus officinalis Sieb. Et Zucc. Fitoterapia. 2023;165:105393.（doi:<https://doi.org/10.1016/j.fitote.2022.105393>）

163. Liu Z-X, Liu C-T, Liu Q-B, Ren J, Li L-Z, Huang X-X, et al. Iridoid glycosides from the flower buds of Lonicera japonica and their nitric oxide production and α-glucosidase inhibitory activities. Journal of Functional Foods. 2015;18:512-9.（doi:<https://doi.org/10.1016/j.jff.2015.08.017>）

164. Yang M, Hao Z, Wang X, Zhou S, Zhu D, Yang Y, et al. Neocornuside A-D, Four Novel Iridoid Glycosides from Fruits of Cornus officinalis and Their Antidiabetic Activity. Molecules. 2022;27(15).（doi:10.3390/molecules27154732）

165. Hao Z-Y, Wang X-L, Yang M, Cao B, Zeng M-N, Zhou S-Q, et al. Minor iridoid glycosides from the fruits of Cornus officinalis Sieb. et Zucc. and their anti-diabetic bioactivities. Phytochemistry. 2023;205:113505.（doi:<https://doi.org/10.1016/j.phytochem.2022.113505>）

166. Hua D, Luo W, Duan J, Jin D, Zhou X, Sun C, et al. Screening and identification of potent α-glycosidase inhibitors from Gardenia jasminoides Ellis. South African Journal of Botany. 2018;119:377-82.（doi:<https://doi.org/10.1016/j.sajb.2018.10.004>）

167. Guan L, Gong D, Yang S, Shen N, Zhang S, Li Y, et al. Genipin ameliorates diet-induced obesity via promoting lipid mobilization and browning of white adipose tissue in rats. Phytotherapy Research. 2018;32(4):723-32.（doi:<https://doi.org/10.1002/ptr.6022>）

168. Ma CJ, Nie AF, Zhang ZJ, Zhang ZG, Du L, Li XY, et al. Genipin stimulates glucose transport in C2C12 myotubes via an IRS-1 and calcium-dependent mechanism. J Endocrinol. 2013;216(3):353-62.（doi:10.1530/joe-11-0473）

169. Guan L, Feng H, Gong D, Zhao X, Cai L, Wu Q, et al. Genipin ameliorates age-related insulin resistance through inhibiting hepatic oxidative stress and mitochondrial dysfunction. Experimental Gerontology. 2013;48(12):1387-94.（doi:<https://doi.org/10.1016/j.exger.2013.09.001>）

170. Liu J, Song C, Nie C, Sun Y, Wang Y, Xue L, et al. A novel regulatory mechanism of geniposide for improving glucose homeostasis mediated by circulating RBP4. Phytomedicine. 2022;95:153862.（doi:<https://doi.org/10.1016/j.phymed.2021.153862>）

171. Guo L, Zheng X, Liu J, Yin Z. Geniposide Suppresses Hepatic Glucose Production via AMPK in HepG2 Cells. Biol Pharm Bull. 2016;39(4):484-91.（doi:10.1248/bpb.b15-00591）

172. Zhou H, Zhang S, Chen L, Liu Y, Shen L, Zhang J. Effective Therapeutic Verification of Crocin I, Geniposide, and Gardenia (Gardenia jasminoides Ellis) on Type 2 Diabetes Mellitus In Vivo and In Vitro. Foods. 2023;12(8).（doi:10.3390/foods12081668）

173. He K, Song S, Zou Z, Feng M, Wang D, Wang Y, et al. The Hypoglycemic and Synergistic Effect of Loganin, Morroniside, and Ursolic Acid Isolated from the Fruits of Cornus officinalis. Phytother Res. 2016;30(2):283-91.（doi:10.1002/ptr.5529）

174. Yokozawa T, Yamabe N, Kim HY, Kang KS, Hur JM, Park CH, et al. Protective Effects of Morroniside Isolated from Corni Fructus against Renal Damage in Streptozotocin-Induced Diabetic Rats. Biological and Pharmaceutical Bulletin. 2008;31(7):1422-8.（doi:10.1248/bpb.31.1422）

175. Cheng S, Tang M, Wang Y. Effect of Astragalus Polysaccharides on Postprandial 1 Hour Blood Glucose in Type 2 Diabetic Rats. Traditional Chinese Drug Research and Clinical Pharmacology. 2020;31(04):396-401.（doi:10.19378/j.issn.1003-9783.2020.04.005）

176. Wu Y, Ou-Yang JP, Wu K, Wang Y, Zhou YF, Wen CY. Hypoglycemic effect of Astragalus polysaccharide and its effect on PTP1B. Acta Pharmacol Sin. 2005;26(3):345-52.（doi:10.1111/j.1745-7254.2005.00062.x）

177. Mao XQ, Yu F, Wang N, Wu Y, Zou F, Wu K, et al. Hypoglycemic effect of polysaccharide enriched extract of Astragalus membranaceus in diet induced insulin resistant C57BL/6J mice and its potential mechanism. Phytomedicine. 2009;16(5):416-25.（doi:10.1016/j.phymed.2008.12.011）

178. Liu J, Zhang JF, Lu JZ, Zhang DL, Li K, Su K, et al. Astragalus polysaccharide stimulates glucose uptake in L6 myotubes through AMPK activation and AS160/TBC1D4 phosphorylation. Acta Pharmacol Sin. 2013;34(1):137-45.（doi:10.1038/aps.2012.133）

179. Song Q, Zou J, Li D, Cheng SW, Li KLS, Yang X, et al. Gastrointestinal metabolism of Astragalus membranaceus polysaccharides and its related hypoglycemic mechanism based on gut microbial transformation. International Journal of Biological Macromolecules. 2024;280:135847.（doi:<https://doi.org/10.1016/j.ijbiomac.2024.135847>）

180. Chen X, Chen C, Fu X. Hypoglycemic activity in vitro and vivo of a water-soluble polysaccharide from Astragalus membranaceus. Food Funct. 2022;13(21):11210-22.（doi:10.1039/d2fo02298b）

181. Chen X, Chen C, Fu X. Hypoglycemic effect of the polysaccharides from Astragalus membranaceus on type 2 diabetic mice based on the "gut microbiota-mucosal barrier". Food Funct. 2022;13(19):10121-33.（doi:10.1039/d2fo02300h）

182. Liu Y, Liu W, Li J, Tang S, Wang M, Huang W, et al. A polysaccharide extracted from Astragalus membranaceus residue improves cognitive dysfunction by altering gut microbiota in diabetic mice. Carbohydr Polym. 2019;205:500-12.（doi:10.1016/j.carbpol.2018.10.041）

183. Li J, Ye G, Zhou Z, Wang J, Wang J, Ju T, et al. Ultrasound-assisted extraction of polysaccharide from Allium chinense G. Don epidermal waste: Evaluation of extraction mechanism, physicochemical properties, and bioactivities. Ultrason Sonochem. 2025;112:107210.（doi:10.1016/j.ultsonch.2024.107210）

184. Zhang Y, He Z, Liu X, Chen Z, Sun J, Wu Z, et al. Oral administration of Angelica sinensis polysaccharide protects against pancreatic islets failure in type 2 diabetic mice: Pancreatic β-cell apoptosis inhibition. Journal of Functional Foods. 2019;54:361-70.（doi:<https://doi.org/10.1016/j.jff.2019.01.037>）

185. Wang K, Tang Z, Zheng Z, Cao P, Shui W, Li Q, et al. Protective effects of Angelica sinensis polysaccharide against hyperglycemia and liver injury in multiple low-dose streptozotocin-induced type 2 diabetic BALB/c mice. Food Funct. 2016;7(12):4889-97.（doi:10.1039/c6fo01196a）

186. Wang F, Hu H, Li Y, Zhong J, Pan H, Sheng Y, et al. Characterization, antioxidant and α-amylase inhibition of polysaccharides and phosphorylated derivatives from finger citron (Citrus medica L. var. sarcodactylis Swingle). Food Bioscience. 2024;59:103985.（doi:<https://doi.org/10.1016/j.fbio.2024.103985>）

187. Yang，Y. Hypoglycemic Effect and Mechanism of Polysaccharides from Finger Citron from Guangdong Province Based on Enzyme Activities and Cell Model. Food Science. 2022;43(23):149-57

188. Wen Y, Chen J. Optimization of Ultrasound-Assisted Deep Eutectic Solvent Extraction, Characterization, and Bioactivities of Polysaccharide from Pericarpium Citri Reticulatae. Appl Biochem Biotechnol. 2024;196(12):8700-23.（doi:10.1007/s12010-024-04990-8）

189. Liu W, Lv X, Huang W, Yao W, Gao X. Characterization and hypoglycemic effect of a neutral polysaccharide extracted from the residue of Codonopsis Pilosula. Carbohydrate Polymers. 2018;197:215-26.（doi:<https://doi.org/10.1016/j.carbpol.2018.05.067>）

190. Yang M, Wang A, Tang X, Wang X, Leng F, Wang Y. Structure identification and activity evaluation of polysaccharide from Codonopsis pilosula (C. pilosula nannf. Var. modesta (nannf.) L. T. Shen). Food Bioscience. 2024;62:104973.（doi:<https://doi.org/10.1016/j.fbio.2024.104973>）

191. Xia T, Liu CS, Hu YN, Luo ZY, Chen FL, Yuan LX, et al. Coix seed polysaccharides alleviate type 2 diabetes mellitus via gut microbiota-derived short-chain fatty acids activation of IGF1/PI3K/AKT signaling. Food Res Int. 2021;150(Pt A):110717.（doi:10.1016/j.foodres.2021.110717）

192. Fu C-Y, Ren L, Liu W-J, Sui Y, Nong Q-N, Xiao Q-H, et al. Structural characteristics of a hypoglycemic polysaccharide from Fructus Corni. Carbohydrate Research. 2021;506:108358.（doi:<https://doi.org/10.1016/j.carres.2021.108358>）

193. Zhong L, Jiang Z, Wang J. Optimization of Extraction Technology of Hawthorn Polysaccharides and Its Hypoglycemic and Hypolipidemic Activity. Science and Technology of Food Industry. 2019;40(13):119-24+47.（doi:10.13386/j.issn1002-0306.2019.13.020）

194. Cheng Z, Hu M, Tao J, Yang H, Yan P, An G, et al. The protective effects of Chinese yam polysaccharide against obesity-induced insulin resistance. Journal of Functional Foods. 2019;55:238-47.（doi:<https://doi.org/10.1016/j.jff.2019.02.023>）

195. Zhi F, xing Q, Wang Y. Effect of Dioscorea opposita Thunb.Polysaccharide on Glycolipid Metabolism and Oxidative Stress in Type 2 Diabetic Rats. Food Science. 2017;38(05):262-6

196. Wang L, Yang Y-p, Tian Y, Huang S-c, Ruan Y, Wen C-n, et al. Purification and characterization of two non-starch polysaccharides from bulbils of Dioscorea opposita Thunb. ‘Tiegun’ and their antioxidant and hypoglycemic activity. Journal of the Science of Food and Agriculture. 2025;105(10):5470-80.（doi:<https://doi.org/10.1002/jsfa.14296>）

197. LI W, CHU Y, PANG X. Hypoglycemic Effects of Polysaccharide from Dolichos lablab L. via

Hypothalamic-Pituitary-Adrenal Axis. Science and Technology of Food Industry. 2022;43(07):361-7.（doi:10.13386/j.issn1002-0306.2021070363）

198. Chen S-M, Zeng F-S, Fu W-W, You H-T, Mu X-Y, Chen G-F, et al. White hyacinth bean polysaccharide ameliorates diabetes via microbiota-gut-brain axis in type 2 diabetes mellitus rats. International Journal of Biological Macromolecules. 2023;253:127307.（doi:<https://doi.org/10.1016/j.ijbiomac.2023.127307>）

199. Wang Y-X, Pi J-C, Yao Y-F, Peng X-P, Li W-J, Xie M-Y. Hypoglycemic effects of white hyacinth bean polysaccharide on type 2 diabetes mellitus rats involvement with entero-insular axis and GLP-1 via metabolomics study. International Journal of Biological Macromolecules. 2024;281:136489.（doi:<https://doi.org/10.1016/j.ijbiomac.2024.136489>）

200. Zhang W-N, Su R-N, Gong L-L, Yang W-W, Chen J, Yang R, et al. Structural characterization and in vitro hypoglycemic activity of a glucan from Euryale ferox Salisb. seeds. Carbohydrate Polymers. 2019;209:363-71.（doi:<https://doi.org/10.1016/j.carbpol.2019.01.044>）

201. Jiang C, Jiang D, Xiong Q. Effect of Polysaccharides from Galli Gigerii Endothelium Corneum on Blood Lipid，Blood Glucose and Cellular Immune Function in Diabetes Mellitus Rats with Hyperlipidemia. Chinese Journal of Experimental Traditional Medical Formulae. 2012;18(20):255-8.（doi:10.13422/j.cnki.syfjx.2012.20.074）

202. Zhu K, Nie S, Li C, Lin S, Xing M, Li W, et al. A newly identified polysaccharide from Ganoderma atrum attenuates hyperglycemia and hyperlipidemia. Int J Biol Macromol. 2013;57:142-50.（doi:10.1016/j.ijbiomac.2013.03.009）

203. Xiao C, Wu Q, Zhang J, Xie Y, Cai W, Tan J. Antidiabetic activity of Ganoderma lucidum polysaccharides F31 down-regulated hepatic glucose regulatory enzymes in diabetic mice. J Ethnopharmacol. 2017;196:47-57.（doi:10.1016/j.jep.2016.11.044）

204. Chen M, Xiao D, Liu W, Song Y, Zou B, Li L, et al. Intake of Ganoderma lucidum polysaccharides reverses the disturbed gut microbiota and metabolism in type 2 diabetic rats. Int J Biol Macromol. 2020;155:890-902.（doi:10.1016/j.ijbiomac.2019.11.047）

205. Xu S, Dou Y, Ye B, Wu Q, Wang Y, Hu M, et al. Ganoderma lucidum polysaccharides improve insulin sensitivity by regulating inflammatory cytokines and gut microbiota composition in mice. Journal of Functional Foods. 2017;38:545-52.（doi:<https://doi.org/10.1016/j.jff.2017.09.032>）

206. Ji N, Liu P, Zhang N, Yang S, Zhang M. Comparison on Bioactivities and Characteristics of Polysaccharides From Four Varieties of Gastrodia elata Blume. Front Chem. 2022;10:956724.（doi:10.3389/fchem.2022.956724）

207. WANG J, HONG Y, LIU Z. Study on the Antioxidant and Hypoglycemic Effects of Glycyrrhiza Polysaccharide. Science and Technology of Food Industry. 2023;44(01):398-404.（doi:10.13386/j.issn1002-0306.2022030175）

208. Xiao x, Wu x, He C. Hypoglycemic Effect of Lily Polysaccharides in Type I Diabetic Rats. Food Science. 2014;35(01):209-13

209. Ma C, Bai J, Shao C, Liu J, Zhang Y, Li X, et al. Degradation of blue honeysuckle polysaccharides, structural characteristics and antiglycation and hypoglycemic activities of degraded products. Food Res Int. 2021;143:110281.（doi:10.1016/j.foodres.2021.110281）

210. Zhao X, Wang D, Qin L, Yang X, Gao C. Comparative investigation for hypoglycemic effects of polysaccharides from four substitutes of Lonicera japonica in Chinese medicine. Int J Biol Macromol. 2018;109:12-20.（doi:10.1016/j.ijbiomac.2017.12.073）

211. Li D, Zhang X, Fan Y, Zhang Y, Tao X, Yang J. Lycium barbarum Polysaccharides Improved Glucose Metabolism in Prediabetic Mice by Regulating Duodenal Contraction. Nutrients. 2023;15(20).（doi:10.3390/nu15204437）

212. Ma Q, Zhai R, Xie X, Chen T, Zhang Z, Liu H, et al. Hypoglycemic Effects of Lycium barbarum Polysaccharide in Type 2 Diabetes Mellitus Mice via Modulating Gut Microbiota. Front Nutr. 2022;9:916271.（doi:10.3389/fnut.2022.916271）

213. Zhou W, Yang T, Xu W, Huang Y, Ran L, Yan Y, et al. The polysaccharides from the fruits of Lycium barbarum L. confer anti-diabetic effect by regulating gut microbiota and intestinal barrier. Carbohydr Polym. 2022;291:119626.（doi:10.1016/j.carbpol.2022.119626）

214. Wan F, Ma F, Wu J, Qiao X, Chen M, Li W, et al. Effect of Lycium barbarum Polysaccharide on Decreasing Serum Amyloid A3 Expression through Inhibiting NF-κB Activation in a Mouse Model of Diabetic Nephropathy. Analytical Cellular Pathology. 2022;2022(1):7847135.（doi:<https://doi.org/10.1155/2022/7847135>）

215. Cai H, Yang X, Cai Q, Ren B, Qiu H, Yao Z. Lycium barbarum L. Polysaccharide (LBP) Reduces Glucose Uptake via Down-Regulation of SGLT-1 in Caco2 Cell. Molecules. 2017;22(2).（doi:10.3390/molecules22020341）

216. Fang C, Chen G, Kan J. Comparison on characterization and biological activities of Mentha haplocalyx polysaccharides at different solvent extractions. Int J Biol Macromol. 2020;154:916-28.（doi:10.1016/j.ijbiomac.2020.03.169）

217. Zhang Y, Ren C, Lu G, Mu Z, Cui W, Gao H, et al. Anti-diabetic effect of mulberry leaf polysaccharide by inhibiting pancreatic islet cell apoptosis and ameliorating insulin secretory capacity in diabetic rats. International Immunopharmacology. 2014;22(1):248-57.（doi:<https://doi.org/10.1016/j.intimp.2014.06.039>）

218. Wang P-P, Huang Q, Chen C, You L-J, Liu RH, Luo Z-G, et al. The chemical structure and biological activities of a novel polysaccharide obtained from Fructus Mori and its zinc derivative. Journal of Functional Foods. 2019;54:64-73

219. Jiao Y, Wang X, Jiang X, Kong F, Wang S, Yan C. Antidiabetic effects of Morus alba fruit polysaccharides on high-fat diet- and streptozotocin-induced type 2 diabetes in rats. Journal of Ethnopharmacology. 2017;199:119-27.（doi:<https://doi.org/10.1016/j.jep.2017.02.003>）

220. Chen C, Huang Q, Li C, Fu X. Hypoglycemic effects of a Fructus Mori polysaccharide in vitro and in vivo. Food Funct. 2017;8(7):2523-35.（doi:10.1039/c7fo00417f）

221. Pingping W, Changli H, Qiaoyun L, Kegang W, Xianghua C, Xiong F, et al. Fructus Mori polysaccharides modulate the axial distribution of gut microbiota and fecal metabolites to improve symptoms of hyperglycemia in type 2 diabetic mice. International Journal of Biological Macromolecules. 2025;307:141949.（doi:<https://doi.org/10.1016/j.ijbiomac.2025.141949>）

222. Chen X, Wu J, Fu X, Wang P, Chen C. Fructus mori polysaccharide alleviates diabetic symptoms by regulating intestinal microbiota and intestinal barrier against TLR4/NF-κB pathway. International Journal of Biological Macromolecules. 2023;249:126038.（doi:<https://doi.org/10.1016/j.ijbiomac.2023.126038>）

223. Huang Y, Xie W, Tang T, Chen H, Zhou X. Structural characteristics, antioxidant and hypoglycemic activities of polysaccharides from Mori Fructus based on different extraction methods. Front Nutr. 2023;10:1125831.（doi:10.3389/fnut.2023.1125831）

224. Feng K-L, Huang L, Wu D-T, Li F, Gan R-Y, Qin W, et al. Physicochemical properties and in vitro bioactivities of polysaccharides from lotus leaves extracted by different techniques and solvents. Journal of Food Measurement and Characterization. 2022;16(2):1583-94.（doi:10.1007/s11694-021-01256-3）

225. Wu D-T, Feng K-L, Huang L, Gan R-Y, Hu Y-C, Zou L. Deep Eutectic Solvent-Assisted Extraction, Partially Structural Characterization, and Bioactivities of Acidic Polysaccharides from Lotus Leaves. Foods. 2021;10(10):2330

226. Li W, Wu D-T, Li F, Gan R-Y, Hu Y-C, Zou L. Structural and Biological Properties of Water Soluble Polysaccharides from Lotus Leaves: Effects of Drying Techniques. Molecules. 2021;26(15):4395

227. Wang Q, Zheng Y, Zhuang W, Lu X, Luo X, Zheng B. Genome-wide transcriptional changes in type 2 diabetic mice supplemented with lotus seed resistant starch. Food Chemistry. 2018;264:427-34.（doi:<https://doi.org/10.1016/j.foodchem.2018.05.056>）

228. CHEN R, CHEM D, ZHANG J. Lowering Blood Glucose, Blood Lipids and Antioxidant Effects of Panax quinquefolius Polysaccharide Peptide on Diabetic Mice. Acta Agriculturae Boreali-occidentalis Sinica. 2013;22(11):195-201.（doi:10.7606/j.issn.1004-1389.2013.11.034）

229. Wang S, Zhao Y, Yang J, Liu S, Ni W, Bai X, et al. Ginseng polysaccharide attenuates red blood cells oxidative stress injury by regulating red blood cells glycolysis and liver gluconeogenesis. Journal of Ethnopharmacology. 2023;300:115716.（doi:<https://doi.org/10.1016/j.jep.2022.115716>）

230. Tao L, Zhang J, Lan W, Liu H, Wu Q, Yang S, et al. Neutral oligosaccharides from ginseng (Panax ginseng) residues vs. neutral ginseng polysaccharides: A comparative study of structure elucidation and biological activity. Food Chemistry. 2025;464:141674.（doi:<https://doi.org/10.1016/j.foodchem.2024.141674>）

231. Sun C, Chen Y, Li X, Tai G, Fan Y, Zhou Y. Anti-hyperglycemic and anti-oxidative activities of ginseng polysaccharides in STZ-induced diabetic mice. Food Funct. 2014;5(5):845-8.（doi:10.1039/c3fo60326a）

232. Guan J, Guo P, Chen M, Wang W, Chen X, Li Q, et al. Ultrasound-assisted extraction, purification, sulfation of Perilla leaves polysaccharide and hypoglycemic and hypolipidemic activities. Ultrasonics Sonochemistry. 2025;117:107269.（doi:<https://doi.org/10.1016/j.ultsonch.2025.107269>）

233. SUN G, YUAN L, FANG X. Effects of Purple frutescens Leaves Polysaccharides on Oxidative Stress and PI3K/AKT/GLUT4 Signaling Pathway of Pancreatic Tissues in Diabetes Mellitus Model Mice. China Pharmacy. 2020;31(15):1874-9.（doi:10.6039/j.issn.1001-0408.2020.15.14）

234. Li W, Zhang Y, Zhao X, Fang L, Yang T, Xie J. Optimization of ultrasonic-assisted extraction of Platycodon grandiflorum polysaccharides and evaluation of its structural, antioxidant and hypoglycemic activity. Ultrason Sonochem. 2023;100:106635.（doi:10.1016/j.ultsonch.2023.106635）

235. Yan H, Lu J, Wang Y, Gu W, Yang X, Yu J. Intake of total saponins and polysaccharides from Polygonatum kingianum affects the gut microbiota in diabetic rats. Phytomedicine. 2017;26:45-54.（doi:10.1016/j.phymed.2017.01.007）

236. Chen X, Tong YL, Ren ZM, Chen SS, Mei XY, Zhou QY, et al. Hypoglycemic mechanisms of Polygonatum sibiricum polysaccharide in db/db mice via regulation of glycolysis/gluconeogenesis pathway and alteration of gut microbiota. Heliyon. 2023;9(4):e15484.（doi:10.1016/j.heliyon.2023.e15484）

237. Xie S-Z, Zhang W-J, Liu W, Bai J-B, Xie S-L, Wang T, et al. Physicochemical characterization and hypoglycemic potential of a novel polysaccharide from Polygonatum sibiricum Red through PI3K/Akt mediated signaling pathway. Journal of Functional Foods. 2022;93:105080.（doi:<https://doi.org/10.1016/j.jff.2022.105080>）

238. Li R, Tao A, Yang R, Fan M, Zhang X, Du Z, et al. Structural characterization, hypoglycemic effects and antidiabetic mechanism of a novel polysaccharides from Polygonatum kingianum Coll. et Hemsl. Biomedicine & Pharmacotherapy. 2020;131:110687.（doi:<https://doi.org/10.1016/j.biopha.2020.110687>）

239. Jiang H, Xu Y, Sun C, Adu-Frimpong M, Yu J, Deng W, et al. Physicochemical properties and antidiabetic effects of a polysaccharide obtained from Polygonatum odoratum. International Journal of Food Science and Technology. 2018;53(12):2810-22.（doi:10.1111/ijfs.13896）

240. Zhou J, Hui X, Mao Y. Study on Effect of Polygonatum Polysaccharide on Glycolipid Metabolism in Type 2 Diabetic Rats and Its Mechanism. Chinese Journal of Modern Applied Pharmacy. 2021;38(10):1181-7.（doi:10.13748/j.cnki.issn1007-7693.2021.10.005）

241. Wang Y, Fei Y, Liu L, Xiao Y, Pang Y, Kang J, et al. Polygonatum odoratum Polysaccharides Modulate Gut Microbiota and Mitigate Experimentally Induced Obesity in Rats. Int J Mol Sci. 2018;19(11).（doi:10.3390/ijms19113587）

242. Liu D, HU Y. Extraction of polysaccharide from Polygonatum odoratum effect on mice with type 1 diabetes. Guangzhou Medical Journal. 2009;40(06):49-53.（doi:10.3969/j.issn.1000-8535.2009.06.023）

243. PANG S, PANG X, ZHU Q. Study on the mechanism of Poria cocos polysaccharides on the regulation of gluconeogenesis in liver of type 2 diabetic mellitus model rats. China Pharmacy. 2022;33(13):1581-7.（doi:10.6039/j.issn.1001-0408.2022.13.08）

244. ZHANG S, NING Z. Effect of Poria cocos polysaccharides on glucose and lipid metabolismand oxidative stress disorders in diabetic mice. Natural Product Research and Development. 2025;37(02):195-203.（doi:10.16333/j.1001-6880.2025.2.001）

245. LI F, LI Q, GENG G. Effects of polysaccharide of Portulaca oleracea L.on factorsrelated to glucose and lipid metabolism in diabetic mice. Journal of Northwest A & F University(Natural Science Edition). 2012;40(04):15-20.（doi:10.13207/j.cnki.jnwafu.2012.04.012）

246. Fang J, Yijun Z, Yuqing L, Wen C, Qiuyue L, Jiangping W, et al. Optimal extraction of a polysaccharide with antioxidant and hypoglycemic activities from the root of Radix Puerariae lobatae (Willd.) Ohwi. CyTA - Journal of Food. 2024;22(1):2440028.（doi:10.1080/19476337.2024.2440028）

247. Luo D, Xiaokang D, Jie H, Chengcheng H, Guowei F, and Huang Y. Pueraria lobata root polysaccharide alleviates glucose and lipid metabolic dysfunction in diabetic db/db mice. Pharmaceutical Biology. 2021;59(1):380-8.（doi:10.1080/13880209.2021.1898648）

248. Huo Y, Zhao X, Zhao J, Kong X, Li L, Yuan T, et al. Hypoglycemic effects of Fu-Pen-Zi (Rubus chingii Hu) fruit extracts in streptozotocin-induced type 1 diabetic mice. Journal of Functional Foods. 2021;87:104837.（doi:<https://doi.org/10.1016/j.jff.2021.104837>）

249. Dou Z-M, Chen C, Huang Q, Fu X. The structure, conformation, and hypoglycemic activity of a novel heteropolysaccharide from the blackberry fruit. Food & Function. 2021;12(12):5451-64.（doi:10.1039/D1FO00741F）

250. Xu Y, Liu N, Fu X, Wang L, Yang Y, Ren Y, et al. Structural characteristics, biological, rheological and thermal properties of the polysaccharide and the degraded polysaccharide from raspberry fruits. International Journal of Biological Macromolecules. 2019;132:109-18.（doi:<https://doi.org/10.1016/j.ijbiomac.2019.03.180>）

251. GUO R, ZHAI L, LIU Z. Study on Inhibition on α-glucosidase and Analysis of the Hypoglycemic Composition of Lobster Sauce. Journal of Chinese Medicinal Materials. 2005(01):38-40.（doi:10.13863/j.issn1001-4454.2005.01.022）

252. Yue X, Zhong L, Ye M, Luan Y, Zhang Q, Wang Q. Taraxacum mongolicum polysaccharide promotes white adipocyte browning by regulating miR-134-3p via Akt/GSK-3β signalling. International Journal of Biological Macromolecules. 2024;257:128296.（doi:<https://doi.org/10.1016/j.ijbiomac.2023.128296>）

253. GUO H, CHEN G, ZHAO Y. Ethanol Fractional Precipitation of Dandelion Polysaccharides and its Hypoglycemic Antioxidant Activity. Agricultural Products Processing. 2021(07):1-5+10.（doi:10.16693/j.cnki.1671-9646(X).2021.04.001）

254. WANG L, GAO J, LI T. Preparation, Structural Characterization and Probiotics Proliferation-Promoting Activity of Selenized Dandelion Polysaccharide. Food Science. 2021;42(07):169-75.（doi:10.7506/spkx1002-6630-20200506-043）

255. Chen X, Chen G, Wang Z, Kan J. A comparison of a polysaccharide extracted from ginger (Zingiber officinale) stems and leaves using different methods: preparation, structure characteristics, and biological activities. Int J Biol Macromol. 2020;151:635-49.（doi:10.1016/j.ijbiomac.2020.02.222）

256. Zhao Y, Yang X, Ren D, Wang D, Xuan Y. Preventive effects of jujube polysaccharides on fructose-induced insulin resistance and dyslipidemia in mice. Food Funct. 2014;5(8):1771-8.（doi:10.1039/c3fo60707k）

257. WANG S, CUI J, GU X. Optimization on preparation of hypoglycemic peptides from

apricot( Armeniaca sibirica) hydrolyzed by papain. Science and Technology of Food Industry. 2014;35(09):169-73.（doi:10.13386/j.issn1002-0306.2014.09.028）

258. Cai L, Wu S, Jia C, Cui C, Sun-Waterhouse D. Active peptides with hypoglycemic effect obtained from hemp (Cannabis sativa L) protein through identification, molecular docking, and virtual screening. Food Chemistry. 2023;429:136912.（doi:<https://doi.org/10.1016/j.foodchem.2023.136912>）

259. Liu T, Zhu C, Duan Z, Ma P, Ma X, Fan D. Network Pharmacological Analysis Combined with Experimental Verification to Explore the Effect of Ginseng Polypeptide on the Improvement of Diabetes Symptoms in db/db Mice. J Agric Food Chem. 2024;72(33):18537-51.（doi:10.1021/acs.jafc.4c04949）

260. WANG L, ZHANG H, WANG L. Comparison of Pham aco logical Effects of F ructus Mumei and Its Processed Products. Journal of Chinese Medicinal Materials. 2010;33(03):353-6.（doi:10.13863/j.issn1001-4454.2010.03.015）

261. Zheng Y, Zhu N, Wang J, Zhao N, Yuan C. Crocetin suppresses gestational diabetes in streptozotocin-induced diabetes mellitus rats via suppression of inflammatory reaction. J Food Biochem. 2021;45(9):e13857.（doi:10.1111/jfbc.13857）

262. Kuroda M, Mimaki Y, Nishiyama T, Mae T, Kishida H, Tsukagawa M, et al. Hypoglycemic effects of turmeric (Curcuma longa L. rhizomes) on genetically diabetic KK-Ay mice. Biol Pharm Bull. 2005;28(5):937-9.（doi:10.1248/bpb.28.937）

263. Kalaycıoğlu Z, Gazioğlu I, Erim FB. Comparison of antioxidant, anticholinesterase, and antidiabetic activities of three curcuminoids isolated from Curcuma longa L. Nat Prod Res. 2017;31(24):2914-7.（doi:10.1080/14786419.2017.1299727）

264. Tavaf Z, Dangolani SK, Yousefi R, Panahi F, Shahsavani MB, Khalafi-Nezhad A. Synthesis of new curcumin derivatives as influential antidiabetic α-glucosidase and α-amylase inhibitors with anti-oxidant activity. Carbohydrate Research. 2020;494:108069.（doi:<https://doi.org/10.1016/j.carres.2020.108069>）

265. Miao C, Chen H, Li Y, Guo Y, Xu F, Chen Q, et al. Curcumin and its analog alleviate diabetes-induced damages by regulating inflammation and oxidative stress in brain of diabetic rats. Diabetol Metab Syndr. 2021;13(1):21.（doi:10.1186/s13098-021-00638-3）

266. Corbee RJ, Mes JJ, de Jong GAH, van den Dool RTM, Neumer F, Theis S, et al. Brush border enzyme hydrolysis and glycaemic effects of isomaltulose compared to other saccharides in dogs. J Anim Physiol Anim Nutr (Berl). 2023;107(6):1456-64.（doi:10.1111/jpn.13860）

267. Go HK, Rahman MM, Kim GB, Na CS, Song CH, Kim JS, et al. Antidiabetic Effects of Yam (Dioscorea batatas) and Its Active Constituent, Allantoin, in a Rat Model of Streptozotocin-Induced Diabetes. Nutrients. 2015;7(10):8532-44.（doi:10.3390/nu7105411）

268. Bai Y, Mo K, Wang G, Chen W, Zhang W, Guo Y, et al. Intervention of Gastrodin in Type 2 Diabetes Mellitus and Its Mechanism. Front Pharmacol. 2021;12:710722.（doi:10.3389/fphar.2021.710722）

269. Naganna CM, Prasad KY, Mahendra VP, Ganesan P, Kumar R. Vanillic acid potentiates insulin secretion and prevents pancreatic β-cells cytotoxicity under H(2)O(2)-induced oxidative stress. Mol Biol Rep. 2023;50(2):1311-20.（doi:10.1007/s11033-022-08046-0）

270. Cao P, Xiang S, Liu S, Feng Y, Zhang X, Wu Q, et al. Isolation of an α-glucosidase Inhibitor from Houttuynia cordata Thunb. and Its In vitro and In vivo Hypoglycemic Bioactivity. Plant Foods for Human Nutrition. 2024;79(4):795-802.（doi:10.1007/s11130-024-01217-3）

271. Fatima N, Hafizur RM, Hameed A, Ahmed S, Nisar M, Kabir N. Ellagic acid in Emblica officinalis exerts anti-diabetic activity through the action on β-cells of pancreas. European Journal of Nutrition. 2017;56(2):591-601.（doi:10.1007/s00394-015-1103-y）

272. Jung HA, Ali MY, Choi JS. Promising Inhibitory Effects of Anthraquinones, Naphthopyrone, and Naphthalene Glycosides, from Cassia obtusifolia on α-Glucosidase and Human Protein Tyrosine Phosphatases 1B. Molecules. 2016;22(1).（doi:10.3390/molecules22010028）

273. Yang F, Zou Y, Li C, Li J, Zang Y, Peng X, et al. Discovery of potential hypoglycemic metabolites in Cassiae Semen by coupling UHPLC-QTOF-MS/MS combined plant metabolomics and spectrum-effect relationship analyses. Food Funct. 2022;13(19):10291-304.（doi:10.1039/d2fo00562j）

274. Tang Y, Zhong Z. Obtusifolin treatment improves hyperlipidemia and hyperglycemia: possible mechanism involving oxidative stress. Cell Biochem Biophys. 2014;70(3):1751-7.（doi:10.1007/s12013-014-0124-0）

275. Lekshmi PC, Arimboor R, Indulekha PS, Menon AN. Turmeric (Curcuma longa L.) volatile oil inhibits key enzymes linked to type 2 diabetes. Int J Food Sci Nutr. 2012;63(7):832-4.（doi:10.3109/09637486.2011.607156）

276. Lee J, Lee H-S, Lee S-H. Preventive Activity of Patchouli Alcohol Against Colorectal Cancer and Diabetes. Journal of Medicinal Food. 2023;26(4):255-61.（doi:10.1089/jmf.2022.0130）

277. Liang P, Peng X, Hu Ge, Wu R, Jin J, Ang S, et al. Four new sesquiterpenoids from the aerial parts of Pogostemon cablin (Blanco.) Benth. and their hypoglycemic activity. Fitoterapia. 2024;177:106054.（doi:<https://doi.org/10.1016/j.fitote.2024.106054>）

278. Harwansh RK, Mukherjee K, Bhadra S, Kar A, Bahadur S, Mitra A, et al. Cytochrome P450 inhibitory potential and RP-HPLC standardization of trikatu—A Rasayana from Indian Ayurveda. Journal of Ethnopharmacology. 2014;153(3):674-81.（doi:<https://doi.org/10.1016/j.jep.2014.03.023>）

279. Jiangbo Z, Xuying W, Yuping Z, Xili M, Yiwen Z, Tianbao Z. Effect of astragaloside IV on the embryo-fetal development of Sprague–Dawley rats and New Zealand White rabbits. Journal of Applied Toxicology. 2009;29(5):381-5.（doi:<https://doi.org/10.1002/jat.1422>）

280. Shi Z, Jiang J, Zhao D, Xie B, Li Y, Yu C. Effects of Astragaloside IV on the Pharmacokinetics of Metoprolol in Rats and its Mechanism. Curr Drug Metab. 2022;23(2):131-6.（doi:10.2174/1389200223666220128143133）

281. Wu ML, Lin YP, Wei YL, Du HJ, Ying XQ, Tan WZ, et al. Calycosin Influences the Metabolism of Five Probe Drugs in Rats. Drug Des Devel Ther. 2020;14:429-34.（doi:10.2147/dddt.S236221）

282. Zhang N-Y, Qi M, Zhao L, Zhu M-K, Guo J, Liu J, et al. Curcumin Prevents Aflatoxin B1 Hepatoxicity by Inhibition of Cytochrome P450 Isozymes in Chick Liver. Toxins [Internet]. 2016; 8(11).

283. Ubaid M, Salauddin, Shadani MA, Kawish SM, Albratty M, Makeen HA, et al. Daidzein from Dietary Supplement to a Drug Candidate: An Evaluation of Potential. ACS Omega. 2023;8(36):32271-93.（doi:10.1021/acsomega.3c03741）

284. Vijayakumar TM, Kumar RM, Agrawal A, Dubey GP, Ilango K. Comparative inhibitory potential of selected dietary bioactive polyphenols, phytosterols on CYP3A4 and CYP2D6 with fluorometric high-throughput screening. J Food Sci Technol. 2015;52(7):4537-43.（doi:10.1007/s13197-014-1472-x）

285. Tian J, Wang XQ, Tian Z. Focusing on Formononetin: Recent Perspectives for its Neuroprotective Potentials. Front Pharmacol. 2022;13:905898.（doi:10.3389/fphar.2022.905898）

286. Morshed MN, Akter R, Karim MR, Iqbal S, Kang SC, Yang DC. Bioconversion, Pharmacokinetics, and Therapeutic Mechanisms of Ginsenoside Compound K and Its Analogues for Treating Metabolic Diseases. Curr Issues Mol Biol. 2024;46(3):2320-42.（doi:10.3390/cimb46030148）

287. Xiong Y, Shen L, Liu KJ, Tso P, Xiong Y, Wang G, et al. Antiobesity and antihyperglycemic effects of ginsenoside Rb1 in rats. Diabetes. 2010;59(10):2505-12.（doi:10.2337/db10-0315）

288. Miao L, Yang Y, Li Z, Fang Z, Zhang Y, Han CC. Ginsenoside Rb2: A review of pharmacokinetics and pharmacological effects. J Ginseng Res. 2022;46(2):206-13.（doi:10.1016/j.jgr.2021.11.007）

289. Ghaeminia M, Rajkumar R, Koh HL, Dawe GS, Tan CH. Ginsenoside Rg1 modulates medial prefrontal cortical firing and suppresses the hippocampo-medial prefrontal cortical long-term potentiation. J Ginseng Res. 2018;42(3):298-303.（doi:10.1016/j.jgr.2017.03.010）

290. Størmer FC, Reistad R, Alexander J. Glycyrrhizic acid in liquorice—Evaluation of health hazard. Food and Chemical Toxicology. 1993;31(4):303-12.（doi:<https://doi.org/10.1016/0278-6915(93)90080-I>）

291. Yang L, Gao Y, Bajpai VK, El-Kammar HA, Simal-Gandara J, Cao H, et al. Advance toward isolation, extraction, metabolism and health benefits of kaempferol, a major dietary flavonoid with future perspectives. Crit Rev Food Sci Nutr. 2023;63(16):2773-89.（doi:10.1080/10408398.2021.1980762）

292. Shin JH, Shin SH. A Comprehensive Review of Naringenin, a Promising Phytochemical with Therapeutic Potential. J Microbiol Biotechnol. 2024;34(12):2425-38.（doi:10.4014/jmb.2410.10006）

293. Zhang Q, Zhou Q, Li H. Action and mechanisms of neferine in inflammatory diseases (Review). Mol Med Rep. 2025;32(1).（doi:10.3892/mmr.2025.13539）

294. Vaiyapuri S, Roweth H, Ali MS, Unsworth AJ, Stainer AR, Flora GD, et al. Pharmacological actions of nobiletin in the modulation of platelet function. Br J Pharmacol. 2015;172(16):4133-45.（doi:10.1111/bph.13191）

295. Kim M, Han CH, Lee MY. Enhancement of platelet aggregation by ursolic Acid and oleanolic Acid. Biomol Ther (Seoul). 2014;22(3):254-9.（doi:10.4062/biomolther.2014.008）

296. Fan H, Yang J, Wang H, Zong S, Qu Y, Wang K, et al. Efficacy and safety of puerarin injection as an adjunctive therapy for chronic heart failure: a systematic review and meta-analysis. Front Pharmacol. 2025;16:1516059.（doi:10.3389/fphar.2025.1516059）

297. Avci FG, Sayar NA, Sariyar Akbulut B. An OMIC approach to elaborate the antibacterial mechanisms of different alkaloids. Phytochemistry. 2018;149:123-31.（doi:10.1016/j.phytochem.2017.12.023）

298. Han Jie L, Jantan I, Yusoff SD, Jalil J, Husain K. Sinensetin: An Insight on Its Pharmacological Activities, Mechanisms of Action and Toxicity. Front Pharmacol. 2020;11:553404.（doi:10.3389/fphar.2020.553404）

299. Zhang Z, Wu Y, Wu T. Effect of Astragalus membranaceus and Bagasse Dietary Fiber Biscuits on Glycemic Index. Journal of Clinical and Experimental Medicine. 2007(07):50-1.（doi:10.3969/j.issn.1671-4695.2007.07.027）

300. Geng Z, Jing Y, Ma X. The Develop of Bitter Gourd- Mulberry Leaf Biscuit. Agricultural Products Processing. 2015(17):8-11.（doi:10.16693/j.cnki.1671-9646(X).2015.09.003）

301. Wang Y, Huang L, Zhang H. Preparation of meal replacement powder with Gastrodia elata BL． f． glauca and its ameliorative effect on glycolipid metabolic disorder in type 2 diabetes mice. Food and Fermentation Industries. 2024;50(23):36-44.（doi:10.13995/j.cnki.11-1802/ts.038109）

302. Bai Y, Yang H. Preparation of cake coarse grain biscuit and its auxiliary hypoglycemie test. Cereal & Feed Industry. 2023(05):32-5+40.（doi:10.7633/j.issn.1003-6202.2023.05.008）

303. Cheng Z, Zhang Y, Chen L. Research on Formulation Optimization and Glycemic Index of Compound Hypoglycemic Meal Replacement Powder. Agricultural Products Processing. 2023(24):20-5.（doi:10.16693/j.cnki.1671-9646(X).2023.12.035）

304. Huo，Yangmei., Yao J. Development of Medicine-Food Homologous Compound Meal Replacement Powder and Evaluation of Glycemic Index. Storage and Process. 2022;22(12):61-7

305. Pu X. Preparation of hypoglycemic chocolate and optimization of its 3D printing process. Journal of Food Safety & Quality. 2024;15(14):161-9.（doi:10.19812/j.cnki.jfsq11-5956/ts.20240306009）

306. Ma J, Zhang Y, Ma Q. Formula optimization of low GI barbary wolfberry multigrain meal replacement powder and its hypoglycemic effect. Food and Fermentation Industries. 2024;50(11):233-40.（doi:10.13995/j.cnki.11-1802/ts.035665）

307. Song F, Pu T. Development of meal replacement powder based on dairy with low glycemic index and its hypoglycemic ability in vivo. Food and Fermentation Industries. 2025;51(10):198-205.（doi:10.13995/j.cnki.11-1802/ts.039847）

308. Sheng H, Yan B, Jia Y. Clinical trial of sugar-control ling cakes on blood glucose fluctuation in diabetic patients. Food & Machinery. 2020;36(03):19-23+33.（doi:10.13652/j.issn.1003-5788.2020.03.004）

309. Zhang J, Liang C, Lv W. Development of Astragalus Health Wine. Liquor Making. 2023;50(06):122-4.（doi:10.3969/j.issn.1002-8110.2023.06.029）

310. Yang Z, Zhu X, Wen A, Ran J, Qin L, Zhu Y. Coix Seed-Based Milk Fermented With Limosilactobacillus reuteri Improves Lipid Metabolism and Gut Microbiota in Mice Fed With a High-Fat Diet. Front Nutr. 2022;9:921255.（doi:10.3389/fnut.2022.921255）

311. ZHAO J, Quan W. Development of hypolipemic and hpyerglycemic of dandelion health beverage. Food Science and Technology. 2010;35(11):123-7.（doi:10.13684/j.cnki.spkj.2010.11.063）

312. Li J, Li C, Song B. Improvement in the Glycolipid Metabolism of Diabetic Mice Induced by High-fat Diet with Streptozotocin Supplemented with Ganoderma lucidum Polysaccharide Beverage. Science and Technology of Food Industry. 2024;45(19):9-16.（doi:10.13386/j.issn1002-0306.2023110302）

313. Liu L. Study on the preparation and efficacy of hawthorn-lotus leaf-buckwheat plant beverage. Pharmacy and Clinics of Chinese Materia Medica. 2023;14(01):36-41.（doi:10.3969/j.issn.1674-926X.2023.01.008）

314. Ji Q, Luo Q, Mian H. Optimize the Process of Fermented Goji Vinegar with Kombucha and Conduct Quality Analysis. Beverage Industry. 2023;26(05):41-8.（doi:10.3969/j.issn.1007-7871.2023.05.011）

315. HUANG Y. Study on in vitro antioxidant, hypoglycemic, and hypolipidemic activities of mulberry fermented wine brewed with different varieties. Food and Fermentation Industries. 2024;50(22):150-8.（doi:10.13995/j.cnki.11-1802/ts.038352）
